# Genes with high penetrance for syndromic and non-syndromic autism typically function within the nucleus and regulate gene expression

**DOI:** 10.1186/s13229-016-0082-z

**Published:** 2016-03-15

**Authors:** Emily L. Casanova, Julia L. Sharp, Hrishikesh Chakraborty, Nahid Sultana Sumi, Manuel F. Casanova

**Affiliations:** Department of Biomedical Sciences, University of South Carolina, South Carolina, USA; Department of Pediatrics, Greenville Health System, Patewood Medical Campus, 200A Patewood Dr, Greenville, SC 29615 USA; Department of Mathematical Sciences, Clemson University, Clemson, USA; Department of Biostatistics and Epidemiology, University of South Carolina, South Carolina, USA

**Keywords:** Mental retardation, Epilepsy, Epigenomics, Body patterning, Regulation of gene expression, Chromatin assembly and disassembly

## Abstract

**Background:**

Intellectual disability (ID), autism, and epilepsy share frequent yet variable comorbidities with one another. In order to better understand potential genetic divergence underlying this variable risk, we studied genes responsible for monogenic IDs, grouped according to their autism and epilepsy comorbidities.

**Methods:**

Utilizing 465 different forms of ID with known molecular origins, we accessed available genetic databases in conjunction with gene ontology (GO) to determine whether the genetics underlying ID diverge according to its comorbidities with autism and epilepsy and if genes highly penetrant for autism or epilepsy share distinctive features that set them apart from genes that confer comparatively variable or no apparent risk.

**Results:**

The genetics of ID with autism are relatively enriched in terms associated with nervous system-specific processes and structural morphogenesis. In contrast, we find that ID with highly comorbid epilepsy (HCE) is modestly associated with lipid metabolic processes while ID without autism or epilepsy comorbidity (ID only) is enriched at the Golgi membrane. Highly comorbid autism (HCA) genes, on the other hand, are strongly enriched within the nucleus, are typically involved in regulation of gene expression, and, along with IDs with more variable autism, share strong ties with a core protein-protein interaction (PPI) network integral to basic patterning of the CNS.

**Conclusions:**

According to GO terminology, autism-related gene products are integral to neural development. While it is difficult to draw firm conclusions regarding IDs unassociated with autism, it is clear that the majority of HCA genes are tightly linked with general dysregulation of gene expression, suggesting that disturbances to the chronology of neural maturation and patterning may be key in conferring susceptibility to autism spectrum conditions.

**Electronic supplementary material:**

The online version of this article (doi:10.1186/s13229-016-0082-z) contains supplementary material, which is available to authorized users.

## Background

Intellectual disability (ID), epilepsy, and autism are highly comorbid with one another, suggesting shared etiologies in at least some forms of these conditions. In both autism and ID, epilepsy occurs in approximately one-third of cases, respectively [1, 2]. In those individuals with epilepsy eligible for surgery who have seizure onset prior to 24 months of age, approximately 46 % also have comorbid ID [3]. Meanwhile, about 31 % of autistic children aged 8 have IQs within the ID range, and an additional 23 % fall within the borderline region [4]. These high comorbidity rates stress an etiological commonality among some forms of the conditions, though it leaves unanswered the question of which forms are more susceptible to co-occurrence.

In recent years, there has been increased interest in both the genetic and phenotypic overlap of ID, epilepsy, and autism. Popular foci of study include monogenic syndromes such as tuberous sclerosis (TSC), fragile X (FXS), and Angelman syndromes (AS), whose respective gene mutations can to lead to disturbed neurogenesis and various perturbations in neuroblast and neuronal maturation. This can be inferred by the different yet often overlapping malformations of cortical development (MCD) found in these syndromes. TSC, for instance, is defined in part by the characteristic tubers for which the condition is so named, a form of multifocal cortical and subcortical dysgenesis [5]. In FXS, features of macrocephaly and abnormalities of neuronal migration are also sometimes noted [6, 7]. In addition, mouse models of the syndrome have revealed alterations in neurogenesis and early neuroblast differentiation, particularly affecting the glutamatergic population [8]. Secondary microcephaly is likewise a feature in the majority of those with AS, potentially resulting from disturbances in early neuroblast maturation and subsequent downstream effects on neuronal differentiation and overall cortical structure [9, 10]. In short, commonalities exist between these conditions not necessarily in the precise malformations encountered but in the general presence of MCD, though they may sometimes require microscopic investigation in order to identify. And in fact, numerous types of MCD are commonly found across many forms of ID, epilepsy, and autism, suggesting that these malformations may be indicative of similar physiologies, e.g., excitatory-inhibitory imbalance [11–14].

Because these three conditions overlap so frequently and share phenotypic features such as MCD, we questioned whether the genetics of different forms of ID might segregate according to their comorbidities with either autism or epilepsy, indicating differences in their etiological underpinnings. In particular, we find that ID with high rates of autism comorbidity present with a particularly homogenous genetic profile that has been previously unreported.

## Methods

### Curation

A comprehensive list of forms of ID were accessed from the Mendelian Inheritance in Man (MIM) database [15]. Only conditions with known molecular basis were collected. Keywords for initial search comprised “intellectual disability”, “mental retardation”, “mentally retarded”, “global developmental delay”, “severe developmental delay”, and “profound developmental delay” (for a full listing of OMIM numbers, gene/locus numbers, and associated data, see Additional file 1). Conditions were then curated and removed if: (1) the ID was not a primary feature but was variably expressed; (2) the ID displayed onset later than three years of age; (3) the condition tended to be lethal in infancy or early childhood; (4) the condition had a known yet complex genetic etiology, e.g., large recombination events that include two or more genes (with the exception of chromosome 2p16.3 deletion syndrome which has been directly linked to *NRXN1* mutations); (5) autism was a defining symptom for diagnosis, as in the cases of certain “susceptibility” genes; (6) only one or two cases were noted in the literature; (7) mutations occurred in only a single family; (8) the condition was a chromosome instability syndrome, leading to variable features due to the accumulation of different mutations; or (9) a condition contained an unconfirmed or potentially spurious mapping as indicated by a “?” before the disease name. This led to a final list of 465 different forms of ID and 434 unique genes (some genes whose functions are unknown were also removed from analyses although are still included in the main list. In addition, a small selection of genes was not recognized by gene ontology and therefore was not included within those analyses. Therefore, gene lists sometimes varied minimally between analyses).

**Fig. 1:**
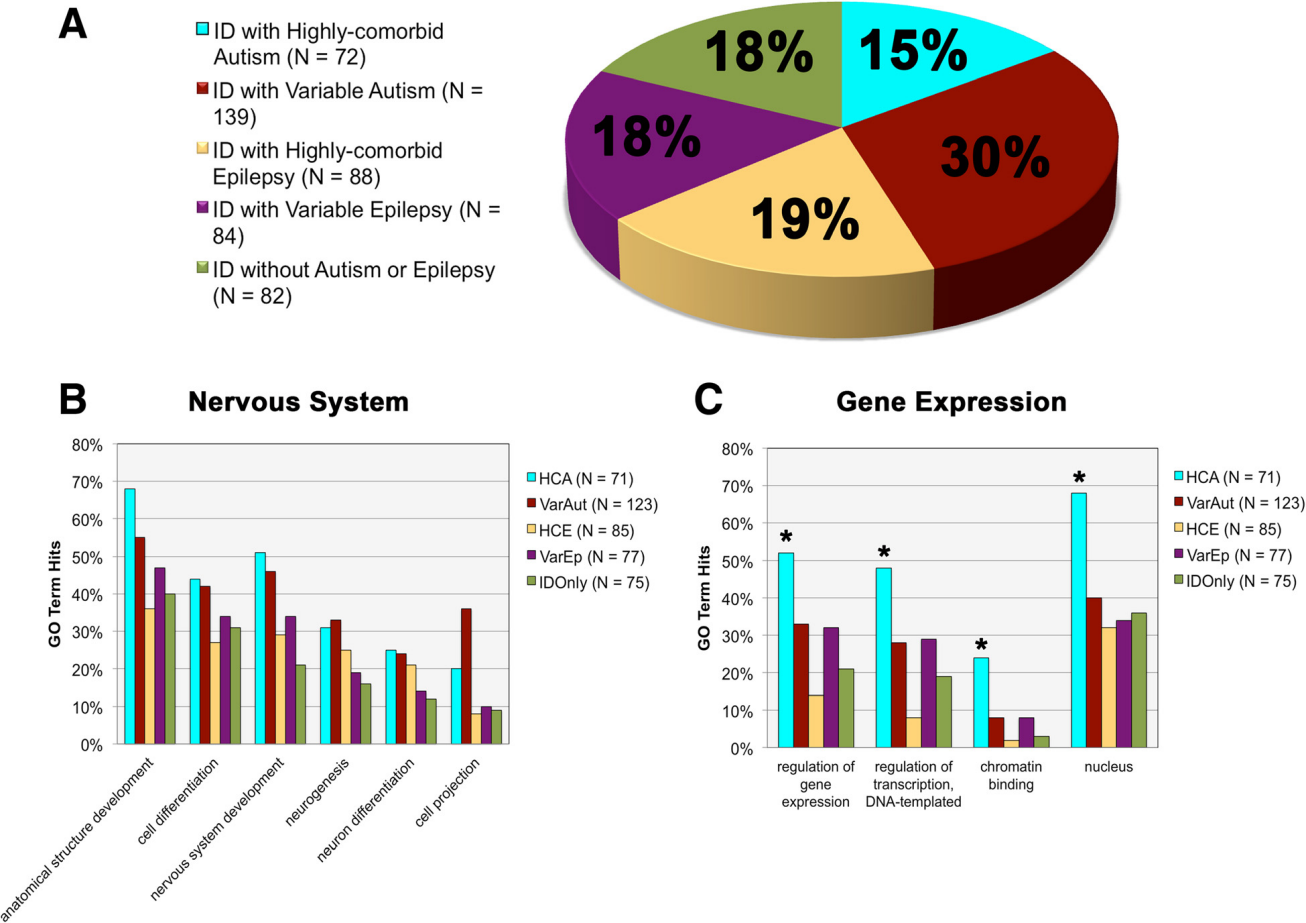
Gene ontology (GO) term enrichments across groups. **a** Proportional breakdown of the different comorbidity groups. **b** Morphogenesis and nervous system-related GO term enrichments according to group. **c** GO term enrichments in processes related to gene expression regulation by group

Associated genes were assigned to one of five categories according to the information available regarding their comorbidities with autism and epilepsy. This information was initially derived from the MIM database and was subsequently confirmed through thorough literature review. Genes were additionally cross-referenced with Pinto et al. [16], whose supplemental material includes a large list of conditions associated with autism and ID. The categories ultimately were: (1) ID with highly comorbid autism (HCA) (*N* = 72 conditions, 71 genes), (2) ID with variable autism (VarAut) (*N* = 139 conditions, 124 genes), (3) ID with highly comorbid epilepsy (HCE) (*N* = 88 conditions, 86 genes), (4) ID with variable epilepsy (VarEp) (*N* = 84 conditions, 78 genes), and (5) ID without autism or epilepsy (ID only) (*N* = 82 conditions, 75 genes) (Fig. 1a).

If a gene was redundant across two or more IDs, those with occurrences of autism were preferentially given preference and placed within HCA or VarAut, followed by epilepsy (HCE, VarEp), and so forth. Conditions were assigned the above comorbidity groups if: (1) there was even minor evidence of overlapping comorbidity reported on MIM or in the broader literature; (2) if, in the case of autism, the associated gene was included under “syndromic”, “high confidence”, “strong candidate”, or “suggestive evidence” headings in the SFARI Gene database or rated 9+ within the AutismKB database; (3) there were no reported instances of epilepsy/seizures but abnormal epileptiform activity had been noted; or (4) the gene was included in the epilepsy gene database, CarpeDB [17–19]. In instances in which a single MIM condition had multiple associated genes, all genes were placed in the same category provided any of those were not already in a superseding category.

For the purposes of dividing all autism-associated IDs into either the HCA or VarAut groups, any condition that had ≥20 % autism comorbidity rating according to intensive literature review was placed within the HCA category; meanwhile, all other IDs with at least two unrelated case studies involving autism or autistic symptomology were placed within VarAut (see Additional file 1 and Additional file 2 for comorbidity references). For the purposes of studying genetic penetrance, HCA and HCE conditions were annotated according to their inheritance patterns (dominant, recessive) and broken into their respective pattern groups for further analysis.

Epileptic comorbidity was rated upon whether epilepsy was reported in any cases within MIM or the larger literature and which were not apparently due to trauma or another identifiable illness; in conditions in which the reported *N* was low for the entire ID (≤3) and only one or a few instances of epilepsy/seizure were noted, these disorders were removed from the analysis entirely. Similar to autism, if epilepsy occurred only within a single family, this condition was not included. For assessing frequency of epilepsy, conditions were included in the HCE group if seizures were listed as a common neurological feature for a given condition within the clinical synopses portion of the MIM database, and this was subsequently corroborated via literature search. The same percentage cut-off for inclusion within HCE (20 %) was used as in HCA.

### Gene ontology

In order to assess associated gene product functions, we analyzed differences in sample frequency for gene ontology (GO) terms according to group across categories of biological process, cellular component, and molecular function [20]. A listing of significantly enriched terms were initially accessed for each gene group, followed by the removal of redundant parent terms and those unusable for direct annotation. Absolute frequencies were then compared for each of these significant terms across all groups. The *prop.test()* function in the statistical computing software, *R*, was used for most statistical analyses. All pairs of proportions were compared using a Chi-square test of two proportions with one degree of freedom. A false discovery rate adjustment was applied to account for multiple comparisons.

The same approach was used to study GO term enrichment across HCA dominant x HCA recessive and HCE dominant x HCE recessive conditions. Meanwhile, ratios of dominant:recessive in HCA vs. HCE were assessed using a two-tailed heteroscedastic *t* test.

For more intensive examination, UniProt/Swiss-Prot and Entrez Gene summaries of gene products’ molecular functions were used as the basis to determine whether a given gene was considered a nuclear epigenetic regulator (transcription factor/repressor, methylator, ubiquitinase, chromatin remodeler, etc.) [21, 22]. The same statistical analyses as used in the main GO experiment were also used here. For the purposes of studying rates of nuclear epigenetic regulators across dominant x recessive subgroups, a proportions comparison was used, without need for correction for multiple comparisons.

In addition, because a large minority of the HCA genes are not currently included or rated within the SFARI Gene Database, we compared the SFARI-only HCA group to the HCA non-SFARI group in terms of number of nuclear epigenetic regulators to ensure that no significant differences existed. This was performed using a two-tailed proportions comparison.

Finally, a thorough literature search was performed to determine which conditions within HCE and VarEp were considered neurodegenerative. HCE and VarEp rates of neurodegeneration were compared using a two-tailed proportions comparison.

### Protein-protein interaction networks

For the protein-protein interaction experiment, each gene group was loaded individually into String 10 alongside a selection of proteins representative of the core PPI network (WNT = CTNNB1, SHH = PTCHD1, NCOR = NCOR1, SWI/SNF = SMARCA1, NOTCH = NOTCH1, ERK1/2 = FGF8, TGF-β/BMP = SMAD4), and confidence data were analyzed [23]. Both the percentage of experimental genes connected with the core PPI and the number of intermediary nodes that lay between the genes of interest and their nearest core PPI neighbors were assessed according to group. For the former analysis, a proportions test with correction for multiple comparisons was used; meanwhile, for the latter, an ANOVA was utilized.

## Results

### Comorbidity data

We studied the comorbidities of a substantial list of IDs with known molecular origins derived from the MIM database. Of the 465 forms of IDs collected, we found that 45 % (*N* = 211) were comorbid with autism in at least a minority of cases. Meanwhile, 15 % (*N* = 72) were highly comorbid with autism, co-occurring in ≥20 % of reported cases. Some of these include conditions well known for autism association, such as FXS and TSC, however, also included conditions less well known, such as non-photosensitive trichothiodystrophy and Mowat-Wilson Syndrome. Meanwhile, 44 % (*N* = 204) of IDs were highly comorbid with epilepsy, while an additional 32 % (*N* = 151) exhibited variable rates of epilepsy. In fact, 55 % (*N* = 116) of autism-related conditions listed epilepsy as a common feature, which was consistent across both HCA and VarAut groups, reinforcing ideas of their shared etiologies (Table 1).

**Table 1:** List of intellectual disabilities highly comorbid with autism, including gene symbols, SFARI ratings, estimates of autism comorbidity, and indications of epilepsy comorbidity

Intellectual disability (OMIM #)	Gene symbol	SFARI rating	Autism comorbidity	References	Epilepsy frequency
Adenylosuccinase Deficiency (#103050)	*ADSL*	S	38 % (*N* = 8)	Jaeken et al. 1988 [36]	Common
Alpha-thalassemia/Mental Retardation Syndrome (#301040)	*ATRX*	6	27 %, males (*N* = 73)	Wada and Gibbons 2003 [37]	Common
Angelman Syndrome (#105830)	*UBE3A*	S	79 % (*N* = 39)	Bonati et al. 2007 [38]	Common
				Trillingsgaard and Østergaard 2004 [39]	
Autosomal Dominant Mental Retardation 1 (#156200)	*MBD5*	3S	100 % (*N* = 14)	Talkowski et al. 2011 [40]	Common
Autosomal Dominant Mental Retardation 5 (#612621)	*SYNGAP1*	1S	60 % (*N* = 10)	Hamdan et al. 2011 [41]	Common
				Carvill et al. 2013 [42]	
				Berryer et al. 2013 [43]	
Autosomal Dominant Mental Retardation 12 (#614562)	*ARID1B*	S	63 % (*N* = 8)	Halgren et al. 2012 [44]	Variable
Autosomal Dominant Mental Retardation 23 (#615761)	*SETD5*	1S	≤71 % (*N* = 7)	Grozeva et al. 2014 [45]	No evidence
Autosomal Dominant Mental Retardation 24 (#615828)	*DEAF1*	2S	75 % (*N* = 4)	Vulto-van Silfhout et al. 2014 [46]	No evidence
Autosomal Dominant Mental Retardation 26 (#615834)	*AUTS2*	3	41 % (*N* = 17)	Beunders et al. 2013 [47]	Variable
Autosomal Dominant Mental Retardation 30 (#616083)	*ZMYND11*	3	29 % (*N* = 7)	Coe et al. 2014 [48]	Variable
Autosomal Recessive Mental Retardation 3 (#608443)	*CC2D1A*	3	31 % (*N* = 16)	Manzini et al. 2014 [49]	Variable
Autosomal Recessive Mental Retardation 38 (#615516)	*HERC2*	NS	86 % (*N* = 7)	Puffenberger et al. 2012 [50]	Common
Athabaskan Brainstem Dysgenesis Syndrome (#601536)	*HOXA1*	S	22 % (*N* = 9)	Tischfield et al. 2005 [51]	Variable
Branched-chain Ketoacid Dehydrogenase Kinase Deficiency (#614923)	*BCKDK*	3	100 % (*N* = 6)	Novarino et al. 2012 [52]	Variable
Brunner Syndrome (#300615)	*MAOA*	4	71 %, males (*N* = 7)	Piton et al. 2014 [53]	Uncommon
				Palmer et al. 2016 [54]	
Cardiofaciocutaneous Syndrome 1 (#115150)	*BRAF*	NS	20 % (*N* = 15)	Nava et al. 2007 [55]	Common
Cardiofaciocutaneous Syndrome 3 (#615279)	*MAP2K1*	NI	63 % (*N* = 8)	Nava et al. 2007 [55]	Common
Cerebral Creatine Deficiency Syndrome 1 (#300352)	*SLC6A8*	4	68 % (*N* = 28)	Dunbar et al. 2014 [56]	Common
Cerebral Creatine Deficiency Syndrome 2 (#612736)	*GAMT*	NI	43 % (*N* = 7)	Cheillan et al. 2012 [57]	Common
Cerebral Creatine Deficiency Syndrome 3 (#612718)	*GATM*	NS	35 % (*N* = 20)	Mercimek-Mahmutoglu et al. 2014 [58]	Variable
CHARGE Syndrome (#214800)	*CHD7*	S	60 % (*N* = 10)	Smith et al. 2005 [59]	Variable
Childhood-onset Epileptic Encephalopathy (#615369)	*CHD2*	2S	50 % (*N* = 6)	Chénier et al. 2014 [60]	Common
Christian-type of X-linked Syndromic Mental Retardation (#300243)	*SLC9A6*	S	89 %, males (*N* = 9)	Pescosolido et al. 2014 [61]	Common
Chromosome 2p16.3 Deletion Syndrome (#614332)	*NRXN1*	2	50 % (*N* = 40)	Dabell et al. 2013 [62]	Variable
				Schaaf et al. 2012 [63]	
Cohen Syndrome (#216550)	*VPS13B*	S	49 % (*N* = 45)	Howlin et al. 2005 [64]	Common
Congenital Rett Syndrome (#613454)	*FOXG1*	5	100 % (*N* = 26)	Kortüm et al. 2011 [65]	Common
Cornelia de Lange Syndrome 1-5 (#122470, 300590, 610759, 614701, 300882)	*NIPBL*	NI	62 % (*N* = 34)	Moss et al. 2008 [66]	Variable
	*SMC1A*				
	*SMC3*				
	*RAD21*				
	*HDAC8*				
Early Infantile Epileptic Encephalopathy 4 (#612164)	*STXBP1*	NS	29 % (*N* = 7)	Barcia et al. 2014 [67]	Common
Early Infantile Epileptic Encephalopathy 6 (#607208)	*SCN1A*	S	24 % (*N* = 37)	Li et al. 2011 [68]	Common
Early Infantile Epileptic Encephalopathy 9 (#300088)	*PCDH19*	S	22 %, females (*N* = 27)	Scheffer et al. 2008 [69]	Common
Early Infantile Epileptic Encephalopathy 24 (#615871)	*HCN1*	NS	66 % (*N* = 6)	Nava et al. 2014 [70]	Common
Fragile X Mental Retardation Syndrome (#300624)	*FMR1*	S	45 % (*N* = 64)	Clifford et al. 2007 [71]	Common
Glass Syndrome (#612313)	*SATB2*	4	29 % (*N* = 7)	Balasubramanian et al. 2011 [72]	Common
Helsmoortel-Van der AA Syndrome (#615873)	*ADNP*	1	100 % (*N* = 11)	Helsmoortel et al. 2014 [73]	Common
				Pescosolido et al. 2014 [61]	
KBG Syndrome (#148050)	*ANKRD11*		33 % (*N* = 9)	Ockeloen et al. 2014 [74]	Uncommon
Kleefstra Syndrome (#610253)	*EHMT1*	3S	most, % unknown (*N* = 20)	Willemsen et al. 2012 [75]	Common
Lowe Oculocerebrorenal Syndrome (#309000)	*OCRL*	NS	71 %, males (*N* = 52)	Oliver et al. 2011 [76]	Common
Lubs X-linked Mental Retardation Syndrome (#300260)	*MECP2*	S	100 %, males (*N* = 18)	Ramocki et al. 2009 [77]	Common
Lujan-Fryns Syndrome (#309520)	*MED12*	6	≤62 %, males (*N* = 32)	Williams 2006 [78]	Common
Marshall-Smith Syndrome (#602535)	*NFIX*	NI	83 % (*N* = 6)	van Balkom et al. 2011 [79]	Variable
Mental Retardation with Language Impairment and Autistic Features (#613670)	*FOXP1*	3	75 % (*N* = 4)	Le Fevre et al. 2013 [80]	Variable
Mowat-Wilson Syndrome (#235730)	*ZEB2*	NI	40 % (*N* = 6)	Evans et al. 2012 [81]	Common
Mucopolysaccharidosis, Type IIIA (#252900)	*SGSH*	NI	29 % (*N* = 73)	Héron et al. 2011 [82]	Common
Muscular Dystrophy-dystroglycanopathy (Congenital with Mental Retardation), Type B3 (#613151)	*POMGNT1*	NS	22 % (*N* = 9)	Hehr et al. 2007 [83]	Variable
Myhre Syndrome (#139210)	*SMAD4*	NS	25 % (*N* = 8)	Caputo et al. 2012 [84]	Variable
Myotonic Dystrophy 1 (#160900)	*DMPK*	S	49 % (*N* = 57)	Ekström et al. 2008 [85]	Variable
Neurodegeneration due to Cerebral Folate Transport Deficiency (#613068)	*FOLR1*	NI	35 % (*N* = 20)	Ramaekers and Blau 2004 [86]	Common
				Steinfeld et al. 2009 [87]	
Nicolaides-Baraitser Syndrome (#601358)	*SMARCA2*	NI	28 % (*N* = 18)	Sousa et al. 2009 [88]	Common
Noonan Syndrome 3 (#609942)	*KRAS*	NI	33 % (*N* = 6)	Nava et al. 2007 [55]	Uncommon
Nonphotosensitive Trichothiodystrophy 1 (#234050)	*MPLKIP*	NI	60 % (*N* = 5)	Heller et al. 2015 [89]	Variable
Noonan Syndrome 3 (#609942)	*KRAS*	NI	33 % (*N* = 6)	Nava et al. 2007 [55]	Variable
Norrie Disease (#310600)	*NDP*	NI	27 %, males (*N* = 56)	Smith et al. 2012 [90]	Uncommon
Phelan-McDermid Syndrome (#606232)	*SHANK3*	1S	52 % (*N* = 130)	Phelan et al. 2001 [91]	Common
				Cusmano-Ozog et al. 2007 [92]	
				Dhar et al. 2010 [93]	
Pitt-Hopkins Syndrome (#610954)	*TCF4*	NS	75 % (*N* = 8)	van Balkom et al. 2011 [79]	Vommon
Renpenning Syndrome 1 (#309500)	*PQBP1*	NI	38 %, males (*N* = 13)	Germanaud et al. 2011 [94]	Variable
Rett Syndrome (#312750)	*MECP2*	S	100 %, females (*N* = 35)	Hagberg et al. 1983 [95]	Common
Schaaf-Yang Syndrome (#615547)	*MAGEL2*	NS	100 % (*N* = 6)	Schaaf et al. 2013 [96]	Common
				Soden et al. 2014 [97]	
Smith-Lemli-Opitz Syndrome (#270400)	*DHCR7*	S	75 % (*N* = 14)	Sikora et al. 2006 [98]	Common
Smith-Magenis Syndrome (#182290)	*RAI1*	S	90 % (*N* = 26)	Laje et al. 2010 [99]	Common
Temtamy Syndrome (#218340)	*C12orf57*	NS	100 % (*N* = 10)	Akizu et al. 2013 [100]	Common
Tuberous Sclerosis 2 (#613254)	*TSC2*	S	40 % (*N* = 103)	Numis et al. 2011 [101]	Common
Warburg Micro Syndrome 4 (#615663)	*TBC1D20*	NI	100 % (*N* = 7)	Liegel et al. 2013 [102]	Common
Wiedemann-Steiner Syndrome (#605130)	*KMT2A*	2S	33 % (*N* = 6)	Jones et al. 2012 [103]	Variable
Wu Type of X-linked Syndromic Mental Retardation (#300699)	*GRIA3*	NI	32 %, males (*N* = 6)	Philips et al. 2014 [104]	Common
X-linked Mental Retardation 1 (#309530)	*IQSEC2*	NI	55 %, males (*N* = 9)	Tran Mau-Them et al. 2013 [105]	Uncommon
				Shoubridge et al. 2010 [106]	
X-linked Mental Retardation 72 (#300271)	*RAB39B*	4	33 %, males (*N* = 9)	Russo et al. 2000 [107]	Common
				Giannandrea et al. 2010 [108]	
X-linked Mental Retardation 98 (#300912)	*KIAA2022*	NS	43 %, males (*N* = 7)	van Maldergem et al. 2013 [109]	Common
X-linked Mental Retardation with or without Seizures (#300419)	*ARX*	S	50 %, males (*N* = 6)	Turner et al. 2002 [110]	Variable
X-linked Syndromic Mental Retardation 14 (#300676)	*UPF3B*	S	50 %, males (*N* = 8)	Tarpey et al. 2007 [111]	Variable

Thirty-two of these genes are either not included in the SFARI Gene Database, are unscored, or are scored as a “6”. SFARI rating system: S = syndromic; 1 = high confidence; 2 = strong evidence; 3 = suggestive evidence; 4 = minimal evidence; 5 = hypothesized; 6 = not supported; NI = not included, NS = included but not scored (see Additional file 3 for references used to estimate comorbidities)

### Trends in gene product function

We went on to study the genetic etiologies of our conditions of interest, investigating GO term associations in the areas of biological process, molecular function, and cellular component (see Table 2 for gene list by category). Both autism groups, HCA and VarAut, exhibited enrichment in *nervous system development* compared to HCE and ID only (*p* = 0.0035–0.052, see Additional file 3 for full statistical results). However, despite differences in this overarching parent term, our comorbidity groups did not differ significantly in the child terms *neurogenesis* (*p* = 0.1218–0.8594), *neuron differentiation* (*p* = 0.3118–1.00), *neuron projection development* (*p* = 0.2608–1.00), and *synaptic transmission* (*p* = 0.5075–0.7988). HCA was mildly enriched in the *regulation of synaptic structure or activity* compared to HCE (*p* = 0.0477) and ID only (*p* = 0.0453), but not compared to the variable groups. In summary, both autism groups exhibit stronger nervous system enrichment than either HCE or ID only, suggesting that gene product involvement in nervous system development may characterize a significant subset of autism risk genes (Fig. 1b).

**Table 2:** Full gene list by category

ID with highly comorbid autism (HCA) *N = 71*	ID with variable autism (VarAut) *N = 124*	ID with highly comorbid epilepsy (HCE) *N = 86*	ID with variable epilepsy (VarEp) *N = 78*	ID without autism or epilepsy (ID only) *N = 75*
*ADNP*	*ACSL4*	*AASS*	*ADAR*	*AAAS*
*ADSL*	*ACY1*	*ACTB*	*ADCK3*	*ADAT3*
*ANKRD11*	*ADGRG1*	*ACTG1*	*ALDH3A2*	*AHDC1*
*ARID1B*	*AFF2*	*ADK*	*AP4B1*	*AIFM1*
*ARX*	*AHI1*	*AGA*	*AP4E1*	*ALX4*
*ATRX*	*ALDH5A1*	*ALDH18A1*	*AP4S1*	*AP1S1*
*AUTS2*	*ALDH7A1*	*ALDH4A1*	*ARID1A*	*AP4M1*
*BCKDK*	*AP1S2*	*ALG13*	*ATIC*	*ARSE*
*BRAF*	*ARHGEF6*	*ALG6*	*BSCL2*	*B4GALT1*
*C12orf57*	*ASL*	*ARG1*	*CACNG2*	*B4GALT7*
*CC2D1A*	*BBS10*	*ARHGEF9*	*CDK5RAP2*	*BBS7*
*CHD2*	*BCKDHA*	*ASPM*	*CENPJ*	*BRWD3*
*CHD7*	*BCKDHB*	*ASXL1*	*CHMP1A*	*C12orf65*
*DEAF1*	*BTD*	*ATP1A2*	*COG8*	*C5orf42*
*DHCR7*	*CACNA1D*	*ATP1A3*	*COL4A2*	*CA8*
*DMPK*	*CAMTA1*	*ATP6V0A2*	*CSPP1*	*CASC5*
*EHMT1*	*CASK*	*ATR*	*CYP27A1*	*CDH15*
*FMR1*	*CBS*	*CCDC88C*	*DCAF17*	*CEP152*
*FOLR1*	*CC2D2A*	*CLN5*	*DHTKD1*	*CLCNKB*
*FOXG1*	*CDKL5*	*CLN8*	*DIP2B*	*COG1*
*FOXP1*	*CDON*	*CLP1*	*DNMT3A*	*COG6*
*GAMT*	*CEP290*	*CTSA*	*EIF2AK3*	*CRADD*
*GATM*	*CEP41*	*CUL4B*	*ESCO2*	*CRBN*
*GRIA3*	*CHKB*	*D2HGDH*	*FAT4*	*CTDP1*
*HCN1*	*CNTNAP2*	*DHCR24*	*FGF14*	*DDHD2*
*HDAC8*	*COG5*	*DHFR*	*FGFR1*	*DDX59*
*HERC2*	*CREBBP*	*DPM1*	*GALE*	*DLAT*
*HOXA1*	*CTCF*	*EFTUD2*	*GALT*	*EMD*
*IQSEC2*	*CTNNB1*	*ELOVL4*	*GATAD2B*	*ENTPD1*
*KIAA2022*	*DAG1*	*EPG5*	*GJC2*	*FGFR3*
*KRAS*	*DBT*	*ERLIN2*	*GRIN1*	*FTCD*
*MAGEL2*	*DCHS1*	*FAM126A*	*HFE*	*GIF*
*MAOA*	*DCX*	*FBXL4*	*IGF1*	*GMPPA*
*MAP2K1*	*DDC*	*GABRA1*	*KAT6B*	*GNPTAB*
*MBD5*	*DEPDC5*	*GMPPB*	*KIAA1279*	*HPRT1*
*MECP2*	*DLG3*	*GRM1*	*KRAS*	*IDUA*
*MED12*	*DMD*	*KANSL1*	*LBR*	*IGBP1*
*MLL*	*DOCK8*	*KCNQ2*	*LINS1*	*INPP5E*
*MPLKIP*	*DPYD*	*KCNT1*	*MANBA*	*IRX5*
*NDP*	*DPYS*	*KCTD7*	*MTRR*	*KCNK9*
*NFIX*	*DYM*	*KIF5C*	*MYCN*	*KIF5A*
*NIPBL*	*DYNC1H1*	*KPTN*	*NIN*	*LARGE*
*NRXN1*	*DYRK1A*	*MGAT2*	*NTRK1*	*LARP7*
*OCRL*	*EP300*	*MLYCD*	*OFD1*	*MAN2B1*
*PCDH19*	*FGFR2*	*MTR*	*PEPD*	*MED23*
*POMGNT1*	*FH*	*NAGA*	*PEX1*	*MIR17HG*
*PQBP1*	*FTSJ1*	*NDE1*	*PIGO*	*NRAS*
*RAB39B*	*GABRB3*	*NSDHL*	*PORCN*	*PACS1*
*RAD21*	*GDI1*	*OCLN*	*PPOX*	*PDE4D*
*RAI1*	*GLYCTK*	*PAK3*	*PTCH1*	*PEX11B*
*SATB2*	*GNS*	*PDHA1*	*PVRL1*	*PEX6*
*SCN1A*	*GRIK2*	*PDX1*	*PYCR1*	*POLR3B*
*SETD5*	*GRIN2A*	*PGAP2*	*RAB23*	*POMT2*
*SGSH*	*GRIN2B*	*PGAP3*	*RAB3GAP1*	*PRKAR1A*
*SHANK3*	*GSS*	*PHGDH*	*RAB3GAP2*	*PTDSS1*
*SLC6A8*	*HCFC1*	*PIGA*	*RBBP8*	*RIT1*
*SLC9A6*	*HDAC4*	*PIGL*	*SAMHD1*	*SKI*
*SMAD4*	*HEPACAM*	*PIK3R2*	*SERAC1*	*SLC4A4*
*SMARCA2*	*HGSNAT*	*PLP1*	*SIL1*	*SOS1*
*SMC1A*	*HPD*	*PNKP*	*SIX3*	*SPTBN2*
*SMC3*	*HRAS*	*PPT1*	*SLC12A6*	*TAF2*
*STXBP1*	*HUWE1*	*QARS*	*SMARCA4*	*TBX1*
*SYNGAP1*	*IL1RAPL1*	*RAB18*	*SMARCB1*	*TECR*
*TBC1D20*	*KANK1*	*RANBP2*	*SOBP*	*THOC6*
*TCF4*	*KCNH1*	*RNASET2*	*SOX3*	*TMCO1*
*TSC2*	*KCNJ10*	*RTTN*	*SRD5A3*	*TMEM237*
*UBE3A*	*KDM5C*	*SEPSECS*	*ST3GAL3*	*TMEM67*
*UPF3B*	*KDM6A*	*SLC19A3*	*STIL*	*TTI2*
*VPS13B*	*KIAA0196*	*SLC2A1*	*SYP*	*UMPS*
*ZEB2*	*KIF7*	*SMS*	*TAT*	*UQCRQ*
*ZMYND11*	*KIRREL3*	*SNIP1*	*TBC1D7*	*WDR81*
	*KMT2D*	*SPTAN1*	*TMEM216*	*XYLT1*
	*L1CAM*	*ST3GAL5*	*TRAPPC9*	*YAP1*
	*L2HGDH*	*SZT2*	*UBE3B*	*ZBTB16*
	*LAMB1*	*TBCE*	*UBR1*	*ZBTB24*
	*MAN1B1*	*TECPR2*	*VLDLR*	
	*MCPH1*	*TRMT10A*	*ZDHHC9*	
	*MEF2C*	*TUBA8*	*ZIC2*	
	*METTL23*	*TUBB2A*		
	*MKKS*	*TUBB3*		
	*MTHFR*	*UBE2A*		
	*NAGLU*	*VPS53*		
	*NDN*	*WDR45*		
	*NR2F1*	*WDR62*		
	*NSD1*	*WWOX*		
	*NSUN2*	*ZSWIM6*		
	*OPHN1*			
	*PAH*			
	*PAX6*			
	*PGM3*			
	*PHF6*			
	*PHF8*			
	*PIGV*			
	*POMT1*			
	*PRSS12*			
	*PTEN*			
	*PTPN11*			
	*RELN*			
	*ROGDI*			
	*RPGRIP1L*			
	*RPS6KA3*			
	*SCN2A*			
	*SCN8A*			
	*SETBP1*			
	*SHH*			
	*SLC16A2*			
	*SLC17A5*			
	*SLC35C1*			
	*SOX10*			
	*SOX11*			
	*SRCAP*			
	*STAMBP*			
	*TCN2*			
	*TCTN3*			
	*TSC1*			
	*TSPAN7*			
	*TUBA1A*			
	*TUBG1*			
	*TUSC3*			
	*UPB1*			
	*USP9X*			
	*ZBTB20*			
	*ZNF711*			
	*ZNF81*			

HCA was also particularly enriched in *anatomical structure development* compared to all groups (*p* = 0.0021–0.0419) except VarAut (*p* = 0.2084), indicating the genes’ probable roles in structural morphogenesis (Fig. 1b). However, above all else, HCA was typified by *regulation of gene expression* (*p* = 0.000–0.0404) and was involved in *regulation of DNA-templated transcription* (*p* = 0.000–0.0406) and *chromatin binding* (*p* = 0.0018–0.0326). Matching its functional enrichment, HCA was strongly enriched within the *nucleus* (*p* = 0.0002–0.0009) in contrast to all other groups and was also modestly enriched at the *chromosome* compared to HCE (*p* = 0.0392) and ID only (*p* = 0.0416) (Fig. 1c).

**Fig. 2:**
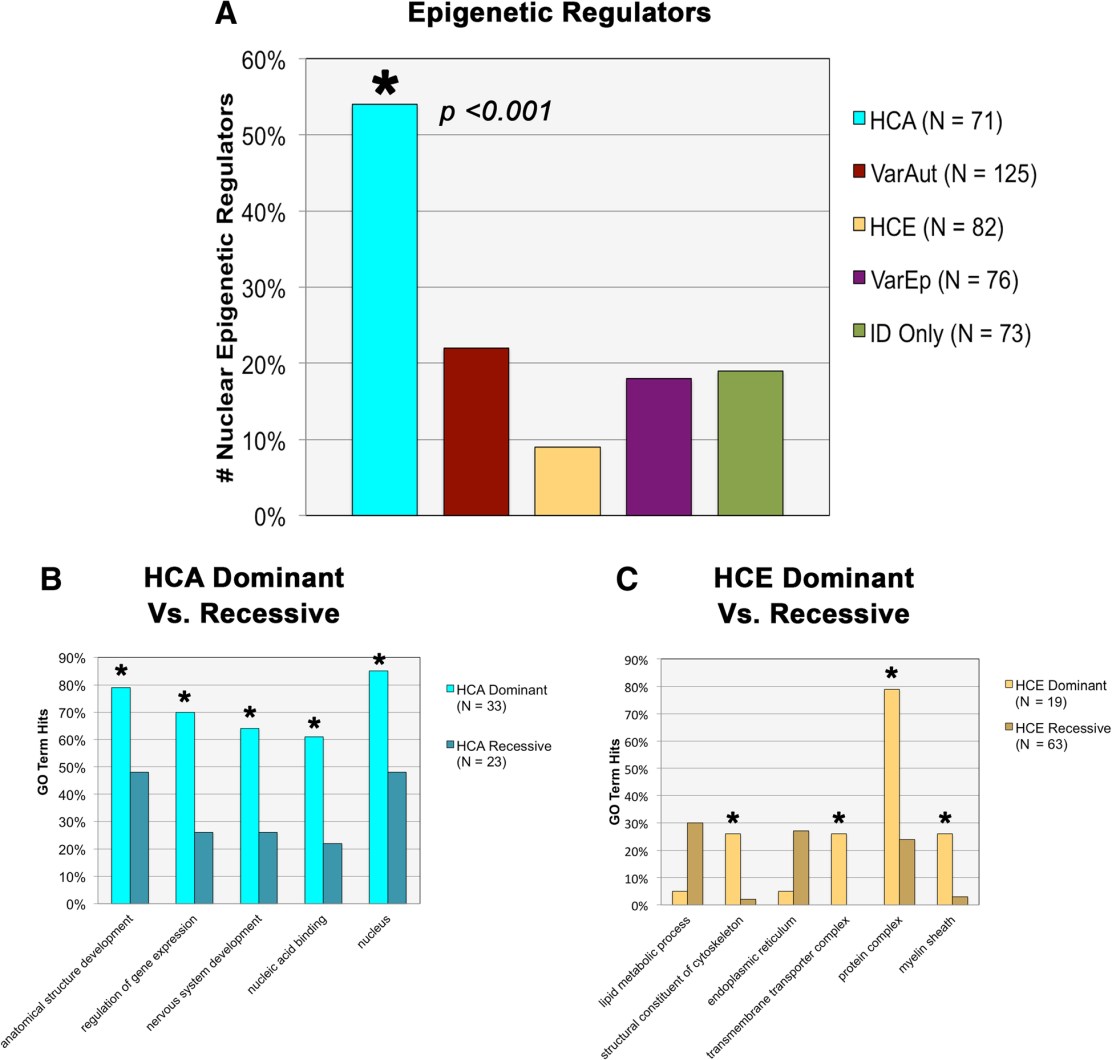
Additional functional and gene ontology (GO) enrichments. **a** Comparison across groups in number of nuclear epigenetic regulators. **b** Comparison of GO terms across dominant vs. recessive HCA subgroups. **c** GO term enrichment across dominant vs. recessive HCE subgroups

UniProt/Swiss-Prot and Entrez Gene analysis further revealed that HCA gene expression regulation was largely carried out through nuclear epigenetic means, such as transcription factors and repressors, methylation regulators, ubiquitin ligases, and other chromatin remodelers, which comprised over half of that gene group, a substantial increase compared to all other comorbidity groups (*p* = 0.000–0.0004) (Fig. 2a). In addition, 45 % of the genes in HCA are not currently included or rated within the SFARI database, yet even with their removal, the SFARI-only HCA group did not differ from those not included within the database in terms of their functional enrichment (*p* = 0.4501, *Z* = 0.8, Diff = −0.1436, 0.3236).

VarAut exhibited similar though more modest trends in functional enrichment as seen within HCA, such as *regulation of gene expression* (*p* = 0.0101) and *regulation of DNA-templated transcription* (*p* = 0.0034), although this was only apparent compared to HCE, the latter which tended to house particularly low enrichment in all of these terms.

HCE, VarEp, and ID Only did not show consistent differences in enrichments in biological processes, with the exception of HCE functional enrichment in *lipid metabolic processes* compared to all groups (*p* = 0.0092–0.0486) except VarEp (*p* = 0.1135). Compartmental enrichments for the non-autism groups were also minimal, with the exception of ID only enrichment within the *Golgi membrane* compared to all groups (*p* = 0.0298) except VarEp (*p* = 0.3132).

For more in-depth analysis, the HCA and HCE comorbidity groups were each divided in two according to their patterns of inheritance (dominant vs. recessive) and were compared against one another for significant GO term enrichments. Significant functional enrichments differentiated both sets of dominant and recessive groups. HCA dominant genes, for instance, were comparatively more enriched than HCA recessive genes in *anatomical structure development* (*p* = 0.0340), *nervous system development* (*p* = 0.0126), *cell differentiation* (*p* = 0.0395), *regulation of gene expression* (*p* = 0.0033), *regulation of DNA-templated transcription* (*p* = 0.0048), and *chromosome organization* (*p* = 0.0232) (Fig. 2b). This suggests that many of the significant GO terms associated with the larger HCA group are primarily driven by this dominantly inherited subgroup.

Meanwhile, the HCE recessive gene group neared significant enrichment in the term *lipid metabolic process* compared to HCE recessive (*p* = 0.0561). In addition, though they likewise did not reach significance, the recessive group was also comparatively enriched in the *endoplasmic reticulum* as well as the *endoplasmic reticulum membrane* (*p* = 0.0913). This suggests that disturbances to protein trafficking through the cell, particularly the endoplasmic reticulum, may be a risk factor for recessive forms of epilepsy.

Dominant HCE genes, in contrast, were enriched in terms related to *structural constituent of cytoskeleton* (*p* = 0.0018), *transmembrane transporter complex* (*p* = 0.0003), *potassium ion transmembrane transporter* (*p* = 0.0018), *protein complex* (*p* < 0.0001), and *myelin sheath* (*p* = 0.007) (Fig. 2c). The functional significance of these associations is not currently well understood.

These results together suggest that dominant and recessive patterns of inheritance may diverge according to gene function, though the reasons for this are currently unknown. It is possible that haploinsufficiency may be more or less detrimental according to broader groups of protein function, leading to variations in penetrance. In addition, we also found that the HCA group had a higher ratio of dominant:recessive disorders than HCE, though the relevance of this also cannot currently be determined and may simply be a reflection of the divergent classes of functional enrichment (*p* < 0.0001).

### Protein-protein interaction network data

**Fig. 3:**
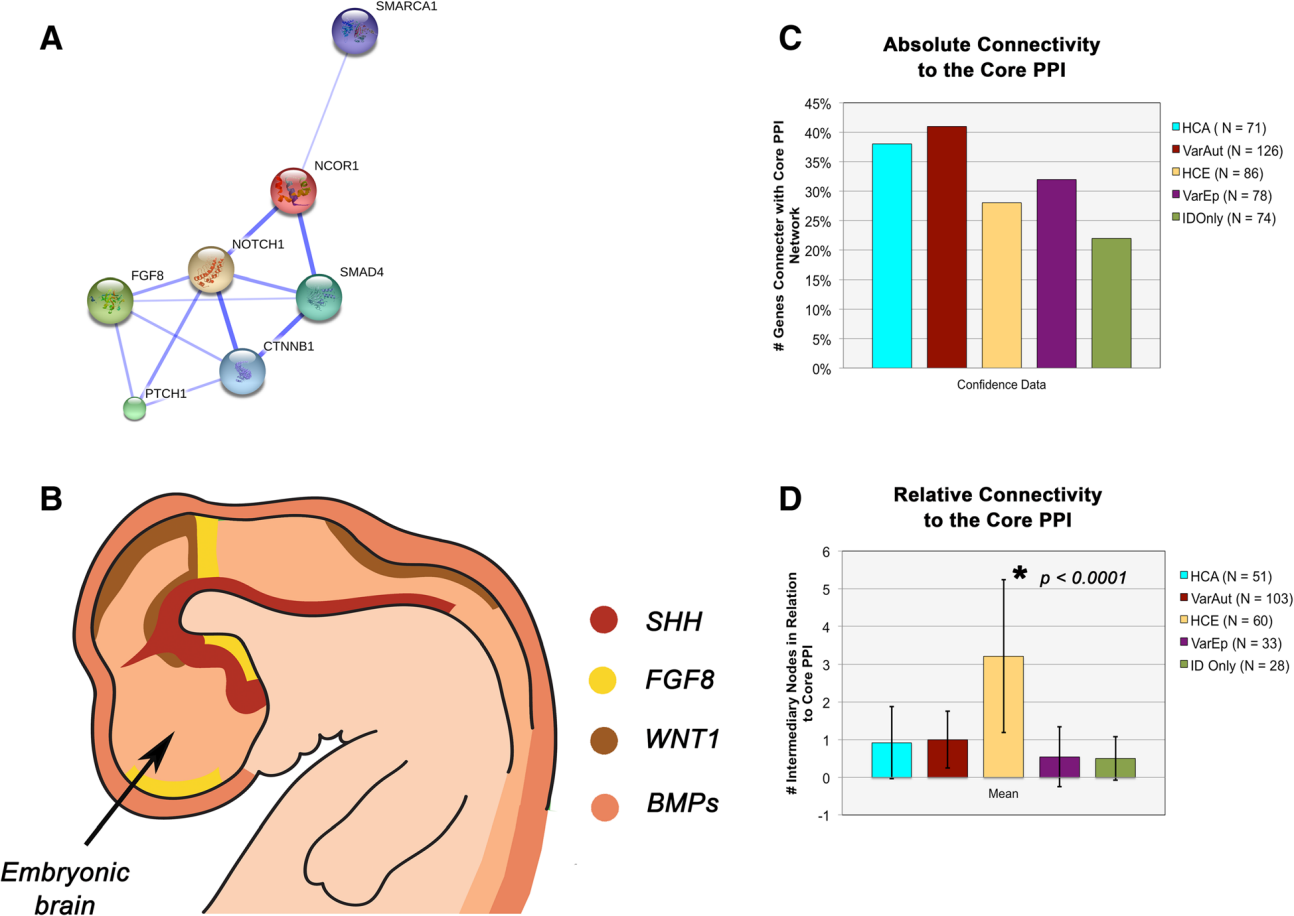
Central nervous system patterning. **a** String 10 results for general interaction of the core PPI within itself. **b** General locations of the major embryonic organizing centers of the central nervous system that underlie variations in its dorsoventral and rostrocaudal patterning. **c** Results across groups for absolute connectivity of experimental proteins to the core PPI network. **d** Network tightness of proteins surrounding the core PPI network, based upon the average number of intermediary nodes between a target protein and its nearest core PPI neighbor (Figure B adapted from Wurst and Bally-Cuif. Nat Rev Neurosci 2001;2(2):99-108)

Upon further study of our gene groups, we find that neither of our autism groups differ significantly from one another in their connectivity to the core PPI network, either in the number of proteins that connect to the core PPI (*p* = 0.1053) nor in the number of intermediary nodes that lay between our proteins and their nearest core PPI neighbor (network “tightness”) (*p* = 0.6098) (Fig. 3c, d). In addition, HCA does not differ from HCE in terms of the number of proteins that connect with the network (*p* = 0.9151), but they do vary according to the tightness of the networks surrounding the core (*p* < 0.0001). Meanwhile, HCA and VarAut exhibit a larger core network than both VarEp (*p* = 0.0001–0.0011) and ID only (*p* = 0.0001–0.0003), but the level of network tightness does not differ significantly. Overall, our autism groups exhibit a larger, tighter protein network surrounding the core PPI compared to all other groups.

The core PPI network is a single extensive protein network integral for patterning of the central nervous system (e.g., dorsoventral patterning) as well as later processes of neural maturation and ongoing plasticity. This network includes morphogens such as Wingless Integration Site (WNT), NOTCH, nuclear receptor corepressor (NCOR), SWItch/Sucrose Non-Fermentable (SWI/SNF), Sonic Hedgehog (SHH), transforming growth factor-β (TGF-β), bone morphogenetic proteins (BMP), and extracellular signal-related kinase 1 and 2 (ERK1/2)—a functional module with considerable overlap with recent reports by Hormozdiari et al. [24] pinpointing a particularly enriched protein network in autism that centers around WNT, NOTCH, SWI/SNF, and NCOR [25].

Why the VarEp group diverged from HCE somewhat in the String analysis, as well as various GO term enrichments, is currently unknown. However, the HCE group (*N* = 88) contained a significantly greater number of neurodegenerative conditions compared to VarEp (*N* = 84) (*z* = 2.5, *p* = 0.0119, Diff = 0.0375, 0.3025), suggesting that the prevalence of neurodegeneration could underlie divergence in their respective etiologies.

## Discussion

### Research implications

Despite that the rare conditions reported here within the HCA group display very high rates of autism comorbidity, genes associated with close to half of these conditions are not currently included within the SFARI Gene Database, are included but unscored, or are scored as “6” indicating that the “evidence does not support a role in [autism spectrum disorders]” (Table 1) [19]. In addition, 69 % of HCA genes that are scored in SFARI but not considered “syndromic” do in fact present with multiple physical dysmorphia, such as complex craniofacial malformations, indicating that with further investigation, these genes should and are likely to be subsumed under this umbrella category.

Some of the non-SFARI conditions that are used in this study are based on small numbers of patients and likely require further in depth diagnostics to confirm the presence of autism symptoms in these conditions at a higher-than-expected rate. In addition, a number of the studies is also hampered by poor diagnostic methodology, an issue that has plagued many of the earlier genetics studies, whereas today’s gold standards include the use of the ADOS and ADI-R as well as ID comparison groups for the purposes of research. Most tellingly, however, the removal of these conditions from the HCA group did not change our results; genes with high penetrance for syndromic and nonsyndromic autism are typically localized to the nucleus and are involved in transcription regulation. These results in particular stress the importance of grouping risk genes according to penetrance when possible, because this information was already present within the SFARI database, though is currently lost amidst the other genetic data.

Because genes associated with HCA are overrepresented within the nucleus and tend to directly regulate transcription, this suggests that mutation penetrance for autism may be strongly linked with regulatory, as opposed to enzymatic, transduction, and structural, cellular networks [26]. Transcription factors and regulators are the most common examples within this group; however, other epigenetic regulators, such as heterochromatin remodelers, ubiquitin ligases, and methylation regulators, were also overrepresented. It may be for their phenotypic penetrance that dominantly inherited conditions were so common in the HCA comorbidity group.

In addition, the presence of gene subgroups within HCA that share considerable overlap with a module for autism risk reported by Hormozdiari et al. [24] suggests that a significant portion of these cases, as well as those in VarAut, are rooted in disturbances to patterning of the CNS and ongoing deviations in neural maturation and plasticity. All of these morphogenetic pathways share considerable crosstalk that is foundational to dorsoventral and rostrocaudal patterning, planar cell polarity, locomotion, neuritogenesis, and finally synaptogenesis and plasticity [25, 27–29]. If disturbed, they are likely to affect all stages of neuronal development, spanning from the most foundational to the most nuanced. Further work at the cellular and tissue levels will be required to investigate whether disturbances to patterning may play roles in these conditions and how such patterning defects, alongside later impairments to neuronal development and plasticity, underlie the behavioral and neurological phenotypes.

In support of this, previous work investigating high-risk autism-related genes has suggested that disturbances to neural maturation may be a common theme to autism [30]. Our present results indicate that epigenetic dysregulation could inappropriately suppress or prematurely promote the expression of gene products, leading to chronological changes in the typical developmental process and ultimately to gross structural, microstructural, and physiological perturbations.

For example, the fragile X mental retardation protein (FMRP) associated with FXS normally aids in suppression of translation, thereby controlling timing of neural differentiation. Instead, when the *FMR1* gene is mutated leading to decreased production of FMRP, neurogenesis occurs prematurely [31]. These neurons also fail to express mature markers in a timely fashion (e.g., GAD67), a disparity likely resulting in poor maturation and circuit integration of adult neurons, and absolute or relative macrocephaly, periventricular heterotopias, and volumetric increase in periventricular white matter, further evidence of a pathological heterochrony and disturbances to patterning [6, 7, 32].

In contrast to our autism, variable epilepsy, and ID only groups, ID with highly comorbid epilepsy exhibited particularly low enrichment in nervous system-specific processes, was more often involved in lipid metabolism, and, compared to conditions with variable epilepsy, had higher rates of neurodegeneration. While this evidence is tantalizing, further work is needed to determine whether ID and epilepsy related to neurodegenerative processes follow a different etiological path than those related to general nervous system development as may be seen in ID with autism or variable epilepsies.

In contrast to the other groups, it was clear that the HCA group is surprisingly homogeneous, suggesting that risk for autism lies within a specific and very definable set of molecular events that confer greater risk the further downstream these elements are affected, i.e., at the level of the gene and its product. This likewise suggests that the further upstream a particular risk factor or environmental effector, the more variable the penetrance for autism due to the number of elements that may intercede and alter events, e.g., feedback inhibition. This is strongly suggested by the divergent compartmental enrichments seen in HCA vs. VarAut, in which the former is highly enriched for the nucleus while the latter is mildly enriched throughout numerous cellular compartments and within cell projections in particular. Ultimately, risk is a threshold effect and a risk factor must be closely upstream of its target (e.g., in the case of epigenetic regulators) or, if further upstream, then it must be capable of avoiding feedback inhibition in order to reach threshold in a consistent highly penetrant fashion (e.g., in the case of select sodium channel mutations).

On a similar note, factors that are comparatively less penetrant yet still confer measurable risk suggest the presence of additional variables, e.g., polygenic effects, environmental agencies, etc., in the determination of their etiologies. Given the nature of genetic selection, common gene variants that provide variable autism risk (i.e., common disease-common variant) are more likely to explain a wider breadth of cases than the rare, often de novo, mutations that confer higher penetrance for the phenotype. Although interestingly, a recent study by Alvarez-Mora et al. [33] reported that in a subset of high-functioning cases they studied, over 50 % (6/11) of the identified rare potentially deleterious single nucleotide variants (SNV) occurred within the HCA genes reported here, suggesting that these genes may be targets with variable penetrance dependent upon the specific type of mutation. Sanders et al. [34] have found that highly penetrant deleterious SNVs tend to affect the same genes that are also targets of small copy number variants (CNV) in autism, such as occurs in the monogenic conditions studied here. Meanwhile, individual genes that comprise larger CNVs each confer comparatively lesser risk. It is possible that if rare SNVs tend to overlap HCA genes, less penetrant SNVs (e.g., common variants) may overlap genes typically comprising larger CNVs and reflect polygenic risk.

In the future, we may find that the genetics of autism tends to diverge according to levels of severity, with rare mutations (e.g., small and large CNVs, highly deleterious rare SNVs) responsible for a significant portion of low-functioning individuals with intellectual disability while other rare SNVs and common variants, perhaps even polygenic and/or environmentally driven, play important roles in a larger portion of the moderate-to-high-functioning ranges. This hypothesis is not entirely unlike that proposed by Folstein [35] in which she suggests that autistic individuals with profound ID, complex dysmorphic features, or specific genetic conditions represent phenotypes that are clinically unique compared to the idiopathic autism reported by Kanner. In this case, however, we are suggesting that the genetics, though not necessarily the overall biology, diverges between the two.

### Additional limitations

Aside from the limitations mentioned above concerning questions of the diagnostic reliability of some of the HCA conditions, additional shortcomings of this study involve the availability of information regarding what are typically rare conditions and potential underreporting regarding comorbidities such as autism and epilepsy. There are, for instance, a number of VarAut conditions in which case studies or small group studies reporting autism incidence are available but no larger studies have been performed in order to provide better estimates of co-occurrence. Examples include conditions such as Succinic Semialdehyde Dehydrogenase Deficiency (#271980), Autosomal Dominant Mental Retardation 21 (#615502), and Dihydropyrimidine Dehydrogenase Deficiency (#274270) to name just a few that are likely worthy of more intensive study in relation to autism. Therefore, it is highly likely that some of the conditions presented within this study have been mis-categorized. In order to limit that occurrence to an absolute minimum, various genetic databases were used in conjunction with phenotypic data.

In addition, the fact that we limited our study of autism and epilepsy to forms of ID subsequently limits the potential scope of applicability of our results, although in doing so we were able to estimate comorbidity rates. We therefore hope that future research may elucidate which of the results presented here are applicable to the broader autism spectrum or whether these data solely define a subgroup of autism.

Because genetic mutations are infrequently identical across different individuals with a single form of ID, it is possible that some cases of autism or epilepsy within our variable groups were not due to mutations involving the primary gene associated with the monogenic condition but were instead due to confounding effects of other genes, such as may be seen in larger chromosomal rearrangements. However, most of the results presented here exhibit strong functional patterns and therefore while individual IDs may ultimately be mis-categorized, we are confident that the conclusions regarding the larger groups are relatively sound.

## Conclusions

While there were distinctive genetic differences between groups, particularly between ID with autism vs. ID without, the strongest findings within this study were overwhelmingly those regarding the HCA group. In particular, we find that the majority of genes that confer high risk for autism are located within the nucleus and function as nuclear epigenetic regulators.

Our results also suggest that both autism groups represents a collection of disabilities that share not only the autism and ID phenotypes, but also likely share developmental similarities in disruption to patterning of the central nervous system. Further work by way of molecular and animal studies is still needed to address this hypothesis.

Aside from novel conclusions derived from the genetic data presented here, we also hope that this curated list may be useful for others and can be updated as new information becomes available. In addition, we hope that this study can be used to inform further clinical research in order to better update databases such as SFARI, affecting research foci in future.

## Availability of data and materials

Additional information on the list of monogenic intellectual disabilities used in this study is available through the Online Mendelian Inheritance in Man (OMIM) database accessible through http://www.omim.org/. OMIM numbers are included within the table in Additional file 1.

## Additional files

Additional file 1: Full list of monogenic intellectual disabilities used in this study, gene information, relevant phenotypic data, and short references for categorization. (XLSX 138 kb) Additional file 2: References for Additional file 1. (DOCX 164 kb) Additional file 3: Full statistical results. (DOCX 157 kb)

## Abbreviations

HCA: intellectual disability with highly comorbid autism
HCE: intellectual disability with highly comorbid epilepsy and without autism
ID: intellectual disability
ID only: intellectual disability without autism or epilepsy
VarAut: intellectual disability with variable autism
VarEp: intellectual disability with variable epilepsy
