# Supplementary material for: Genes with high penetrance for syndromic and non-syndromic autism typically function within the nucleus and regulate gene expression
Source: Mol Autism. 2016 Mar 15;7:18. doi: 10.1186/s13229-016-0082-z (PMC4793536; doi:10.1186/s13229-016-0082-z)
Supplement: Additional file 2: — References for Additional file 1. (DOCX 164 kb) [file 13229_2016_82_MOESM2_ESM.docx]

**ADDITIONAL FILE 2 - STATISTICAL RESULTS:**

**Acronyms.**

HCA = Intellectual Disability with Highly-comorbid Autism

VarAut = Intellectual Disability with Variable Autism

HCE = Intellectual Disability with Highly-comorbid Epilepsy

VarEp = Intellectual Disability with Variable Epilepsy

ID Only = Intellectual Disability without Autism or Epilepsy

HCA Dominant = HCA conditions with dominant inheritance

HCA Recessive = HCA conditions with recessive inheritance

HCE Dominant = HCE conditions with dominant inheritance

HCE Recessive = HCE conditions with recessive inheritance

* = significant p-value between 0.01-0.05

** = significant p-value <0.01

------------------------------------------------------------------------------------------------------------------------------------------------------------------

**Analysis Series 1.** Comparison of Gene Ontology (GO) terms across ID groups. The *prop.test()* function in the statistical computing software, *R*, was used for the statistical analyses. All pairs of proportions were compared using a Chi-square test of two proportions with one degree of freedom. A false discovery rate adjustment was applied to account for multiple comparisons.

**HCA, N = 71**

**VarAut, N = 124**

**HCE, N = 86**

**VarEp, N = 78**

**ID Only, N = 75**

| **GO Biological Process** |  | **HCA**  **p-value** | **HCA**  **Chi** | **VarAut**  **p-value** | **VarAut**  **Chi** | **HCE**  **p-value** | **HCE**  **Chi** | **VarEp**  **p-value** | **VarEp**  **Chi** |
| --- | --- | --- | --- | --- | --- | --- | --- | --- | --- |
| *anatomical structure development* | **VarAut** | 0.2084 | 2.3532 | NA | NA | NA | NA | NA | NA |
| “ | **HCE** | **0.0021 | 13.7834 | *0.0381 | 6.3989 | NA | NA | NA | NA |
| “ | **VarEp** | *0.0419 | 5.7223 | 0.379 | 1.0602 | 0.3472 | 1.3629 | NA | NA |
| “ | **ID Only** | **0.0075 | 10.0884 | 0.1047 | 3.7642 | 0.7675 | 0.0874 | 0.5544 | 0.4572 |
|  |  | **HCA**  **p-value** | **HCA**  **Chi** | **VarAut**  **p-value** | **VarAut**  **Chi** | **HCE**  **p-value** | **HCE**  **Chi** | **VarEp**  **p-value** | **VarEp**  **Chi** |
| *axon development* | **VarAut** | 1 | 0.0461 | NA | NA | NA | NA | NA | NA |
| “ | **HCE** | 1 | 0.0661 | 1 | 0.000 | NA | NA | NA | NA |
| “ | **VarEp** | 0.4409 | 2.0798 | 0.4409 | 1.5013 | 0.5058 | 1.0589 | NA | NA |
| “ | **ID Only** | 0.4409 | 2.7853 | 0.4409 | 2.157 | 0.4409 | 1.6108 | 1 | 0.000 |
|  |  | **HCA**  **p-value** | **HCA**  **Chi** | **VarAut**  **p-value** | **VarAut**  **Chi** | **HCE**  **p-value** | **HCE**  **Chi** | **VarEp**  **p-value** | **VarEp**  **Chi** |
| *carbohydrate metabolic process* | **VarAut** | 0.4448 | 1.2327 | NA | NA | NA | NA | NA | NA |
| “ | **HCE** | 0.2252 | 4.017 | 0.4599 | 0.9812 | NA | NA | NA | NA |
| “ | **VarEp** | 0.7169 | 0.3168 | 0.8932 | 0.0477 | 0.4318 | 1.5314 | NA | NA |
| “ | **ID Only** | 0.2252 | 5.1491 | 0.4318 | 1.7866 | 0.8932 | 0.018 | 0.4176 | 2.35 |
|  |  | **HCA**  **p-value** | **HCA**  **Chi** | **VarAut**  **p-value** | **VarAut**  **Chi** | **HCE**  **p-value** | **HCE**  **Chi** | **VarEp**  **p-value** | **VarEp**  **Chi** |
| *cell cycle* | **VarAut** | 1 | 0.3915 | NA | NA | NA | NA | NA | NA |
| “ | **HCE** | 1 | 0.0365 | 1 | 1.3285 | NA | NA | NA | NA |
| “ | **VarEp** | 1 | 0.000 | 1 | 0.7452 | 1 | 0.000 | NA | NA |
| “ | **ID Only** | 1 | 0.0164 | 1 | 1.0884 | 1 | 0.000 | 1 | 0.000 |
|  |  | **HCA**  **p-value** | **HCA**  **Chi** | **VarAut**  **p-value** | **VarAut**  **Chi** | **HCE**  **p-value** | **HCE**  **Chi** | **VarEp**  **p-value** | **VarEp**  **Chi** |
| *cell differentiation* | **VarAut** | 0.9703 | 0.0014 | NA | NA | NA | NA | NA | NA |
| “ | **HCE** | 0.2266 | 4.0068 | 0.2266 | 4.4101 | NA | NA | NA | NA |
| “ | **VarEp** | 0.4882 | 1.1382 | 0.4882 | 1.1061 | 0.6416 | 0.5729 | NA | NA |
| “ | **ID Only** | 0.3647 | 2.1146 | 0.3647 | 2.1982 | 0.9045 | 0.1077 | 0.9045 | 0.0553 |
|  |  | **HCA**  **p-value** | **HCA**  **Chi** | **VarAut**  **p-value** | **VarAut**  **Chi** | **HCE**  **p-value** | **HCE**  **Chi** | **VarEp**  **p-value** | **VarEp**  **Chi** |
| *cell projection organization* | **VarAut** | 0.7882 | 0.2416 | NA | NA | NA | NA | NA | NA |
| “ | **HCE** | 0.7882 | 0.1389 | 0.6037 | 1.372 | NA | NA | NA | NA |
| “ | **VarEp** | 0.7882 | 0.4332 | 0.6037 | 2.1134 | 0.9072 | 0.0136 | NA | NA |
| “ | **ID Only** | 0.6037 | 1.5281 | 0.3973 | 4.2293 | 0.7882 | 0.5252 | 0.7882 | 0.1476 |
|  |  | **HCA**  **p-value** | **HCA**  **Chi** | **VarAut**  **p-value** | **VarAut**  **Chi** | **HCE**  **p-value** | **HCE**  **Chi** | **VarEp**  **p-value** | **VarEp**  **Chi** |
| *chromosome organization* | **VarAut** | 0.3036 | 1.903 | NA | NA | NA | NA | NA | NA |
| “ | **HCE** | 0.0853 | 5.6907 | 0.3823 | 1.229 | NA | NA | NA | NA |
| “ | **VarEp** | 0.2656 | 2.6092 | 0.7815 | 0.0769 | 0.7027 | 0.2288 | NA | NA |
| “ | **ID Only** | *0.0243 | 9.1906 | 0.1854 | 3.6634 | 0.6814 | 0.3662 | 0.3036 | 1.7797 |
|  |  | **HCA**  **p-value** | **HCA**  **Chi** | **VarAut**  **p-value** | **VarAut**  **Chi** | **HCE**  **p-value** | **HCE**  **Chi** | **VarEp**  **p-value** | **VarEp**  **Chi** |
| *embryo development* | **VarAut** | 0.567 | 0.47 | NA | NA | NA | NA | NA | NA |
| “ | **HCE** | *0.0107 | 10.6943 | *0.0305 | 7.5186 | NA | NA | NA | NA |
| “ | **VarEp** | 0.567 | 0.4335 | 1 | 0.000 | *0.0444 | 6.1268 | NA | NA |
| “ | **ID Only** | 0.1407 | 3.6432 | 0.3823 | 1.7088 | 0.3964 | 1.2719 | 0.3964 | 1.1795 |
|  |  | **HCA**  **p-value** | **HCA**  **Chi** | **VarAut**  **p-value** | **VarAut**  **Chi** | **HCE**  **p-value** | **HCE**  **Chi** | **VarEp**  **p-value** | **VarEp**  **Chi** |
| *establishment of protein localization* | **VarAut** | 0.8085 | 0.4541 | NA | NA | NA | NA | NA | NA |
| “ | **HCE** | 1 | 0.000 | 0.8085 | 0.3295 | NA | NA | NA | NA |
| “ | **VarEp** | 0.4168 | 2.3531 | 0.0504 | 7.8643 | 0.3793 | 3.1517 | NA | NA |
| “ | **ID Only** | 0.9983 | 0.0163 | 0.504 | 1.3123 | 0.964 | 0.0846 | 0.504 | 1.4353 |
|  |  | **HCA**  **p-value** | **HCA**  **Chi** | **VarAut**  **p-value** | **VarAut**  **Chi** | **HCE**  **p-value** | **HCE**  **Chi** | **VarEp**  **p-value** | **VarEp**  **Chi** |
| *gliogenesis* | **VarAut** | 0.3268 | 2.2839 | NA | NA | NA | NA | NA | NA |
| “ | **HCE** | 1 | 0.000 | 0.3268 | 3.2822 | NA | NA | NA | NA |
| “ | **VarEp** | 1 | 0.005 | 0.3268 | 4.2686 | 1 | 0.000 | NA | NA |
| “ | **ID Only** | 1 | 0.000 | 0.3268 | 2.5665 | 1 | 0.000 | 1 | 0.0004 |
|  |  | **HCA**  **p-value** | **HCA**  **Chi** | **VarAut**  **p-value** | **VarAut**  **Chi** | **HCE**  **p-value** | **HCE**  **Chi** | **VarEp**  **p-value** | **VarEp**  **Chi** |
| *lipid metabolic process* | **VarAut** | 0.7734 | 0.3044 | NA | NA | NA | NA | NA | NA |
| “ | **HCE** | **0.0092 | 9.9845 | **0.0092 | 9.7013 | NA | NA | NA | NA |
| “ | **VarEp** | 0.53 | 1.2425 | 0.7734 | 0.2476 | 0.1135 | 4.0033 | NA | NA |
| “ | **ID Only** | 0.7734 | 0.3643 | 1 | 0.000 | *0.0486 | 5.9655 | 0.91 | 0.0524 |
|  |  | **HCA**  **p-value** | **HCA**  **Chi** | **VarAut**  **p-value** | **VarAut**  **Chi** | **HCE**  **p-value** | **HCE**  **Chi** | **VarEp**  **p-value** | **VarEp**  **Chi** |
| *locomotion* | **VarAut** | 0.8597 | 0.0827 | NA | NA | NA | NA | NA |  |
| “ | **HCE** | 0.8037 | 0.4938 | 0.4545 | 1.7829 | NA | NA | NA | NA |
| “ | **VarEp** | 0.4545 | 1.9553 | 0.4157 | 4.1525 | 0.8532 | 0.2792 | NA | NA |
| “ | **ID Only** | 0.8037 | 0.6952 | 0.4545 | 2.0825 | 1 | 0.000 | 0.8597 | 0.1024 |
|  |  | **HCA**  **p-value** | **HCA**  **Chi** | **VarAut**  **p-value** | **VarAut**  **Chi** | **HCE**  **p-value** | **HCE**  **Chi** | **VarEp**  **p-value** | **VarEp**  **Chi** |
| *nervous system development* | **VarAut** | 0.6697 | 0.1908 | NA | NA | NA | NA | NA | NA |
| “ | **HCE** | *0.036 | 6.4984 | 0.052 | 5.3445 | NA | NA | NA | NA |
| “ | **VarEp** | 0.1098 | 3.6855 | 0.1792 | 2.5883 | 0.6697 | 0.182 | NA | NA |
| “ | **ID Only** | **0.0035 | 12.4697 | **0.0035 | 11.4674 | 0.4048 | 0.9734 | 0.1792 | 2.3481 |
|  |  | **HCA**  **p-value** | **HCA**  **Chi** | **VarAut**  **p-value** | **VarAut**  **Chi** | **HCE**  **p-value** | **HCE**  **Chi** | **VarEp**  **p-value** | **VarEp**  **Chi** |
| *neurogenesis* | **VarAut** | 0.8594 | 0.0314 | NA | NA | NA | NA | NA | NA |
| “ | **HCE** | 0.6776 | 0.482 | 0.4085 | 1.3996 | NA | NA | NA | NA |
| “ | **VarEp** | 0.3855 | 2.0304 | 0.1724 | 3.8467 | 0.6776 | 0.3717 | NA | NA |
| “ | **ID Only** | 0.1724 | 3.7845 | 0.1218 | 6.284 | 0.4085 | 1.3512 | 0.8078 | 0.1219 |
|  |  | **HCA**  **p-value** | **HCA**  **Chi** | **VarAut**  **p-value** | **VarAut**  **Chi** | **HCE**  **p-value** | **HCE**  **Chi** | **VarEp**  **p-value** | **VarEp**  **Chi** |
| *neuron differentiation* | **VarAut** | 1 | 0.000 | NA | NA | NA | NA | NA | NA |
| “ | **HCE** | 0.8861 | 0.1812 | 0.8861 | 0.1394 | NA | NA | NA | NA |
| “ | **VarEp** | 0.3424 | 2.2119 | 0.3424 | 2.3791 | 0.581 | 0.8785 | NA | NA |
| “ | **ID Only** | 0.3118 | 3.4735 | 0.3118 | 3.7726 | 0.3637 | 1.7824 | 0.9552 | 0.0313 |
|  |  | **HCA**  **p-value** | **HCA**  **Chi** | **VarAut**  **p-value** | **VarAut**  **Chi** | **HCE**  **p-value** | **HCE**  **Chi** | **VarEp**  **p-value** | **VarEp**  **Chi** |
| *neuron projection development* | **VarAut** | 0.9124 | 0.1323 | NA | NA | NA | NA | NA | NA |
| “ | **HCE** | 0.9124 | 0.1192 | 1 | 0.000 | NA | NA | NA | NA |
| “ | **VarEp** | 0.2608 | 3.3308 | 0.2608 | 2.288 | 0.2908 | 1.8439 | NA | NA |
| “ | **ID Only** | 0.2608 | 4.0931 | 0.2608 | 2.9804 | 0.2608 | 2.461 | 1 | 0.000 |
|  |  | **HCA**  **p-value** | **HCA**  **Chi** | **VarAut**  **p-value** | **VarAut**  **Chi** | **HCE**  **p-value** | **HCE**  **Chi** | **VarEp**  **p-value** | **VarEp**  **Chi** |
| *organ development* | **VarAut** | **0.0039 | 10.0169 | NA | NA | NA | NA | NA | NA |
| “ | **HCE** | *0.0253 | 6.2177 | **<0.0001 | 37.7606 | NA | NA | NA | NA |
| “ | **VarEp** | 0.4126 | 0.7992 | **<0.0001 | 19.3324 | 0.2125 | 9.3217 | NA | NA |
| “ | **ID Only** | 0.0779 | 3.9548 | **<0.0001 | 30.2197 | 0.798 | 2.7124 | 0.4126 | 1.4353 |
|  |  | **HCA**  **p-value** | **HCA**  **Chi** | **VarAut**  **p-value** | **VarAut**  **Chi** | **HCE**  **p-value** | **HCE**  **Chi** | **VarEp**  **p-value** | **VarEp**  **Chi** |
| *regulation of developmental process* | **VarAut** | 0.166 | 2.9031 | NA | NA | NA | NA | NA | NA |
| “ | **HCE** | **0.0001 | 18.8333 | **0.0092 | 8.9599 | NA | NA | NA | NA |
| “ | **VarEp** | 0.2886 | 1.5046 | 0.9063 | 0.0139 | **0.0092 | 9.3217 | NA | NA |
| “ | **ID Only** | *0.0266 | 6.5244 | 0.3218 | 1.1216 | 0.166 | 2.7124 | 0.2886 | 1.4353 |
|  |  | **HCA**  **p-value** | **HCA**  **Chi** | **VarAut**  **p-value** | **VarAut**  **Chi** | **HCE**  **p-value** | **HCE**  **Chi** | **VarEp**  **p-value** | **VarEp**  **Chi** |
| *regulation of gene expression* | **VarAut** | *0.0312 | 5.8454 | NA | NA | NA | NA | NA | NA |
| “ | **HCE** | **<0.0001 | 24.1888 | *0.0101 | 8.7886 | NA | NA | NA | NA |
| “ | **VarEp** | *0.0404 | 5.0772 | 1 | 0.000 | *0.0239 | 6.7132 | NA | NA |
| “ | **ID Only** | **0.0011 | 13.6414 | 0.1421 | 2.7135 | 0.3578 | 0.9806 | 0.2159 | 1.8593 |
|  |  | **HCA**  **p-value** | **HCA**  **Chi** | **VarAut**  **p-value** | **VarAut**  **Chi** | **HCE**  **p-value** | **HCE**  **Chi** | **VarEp**  **p-value** | **VarEp**  **Chi** |
| *regulation of synaptic structure or activity* | **VarAut** | 0.4656 | 0.9652 | NA | NA | NA | NA | NA | NA |
| “ | **HCE** | *0.0477 | 6.7182 | 0.1469 | 2.9083 | NA | NA | NA | NA |
| “ | **VarEp** | 0.8278 | 0.1908 | 0.9505 | 0.0332 | 0.1003 | 3.8367 | NA | NA |
| “ | **ID Only** | *0.0453 | 8.0587 | 0.1003 | 4.1864 | 1 | 0.000 | 0.0741 | 5.2278 |
|  |  | **HCA**  **p-value** | **HCA**  **Chi** | **VarAut**  **p-value** | **VarAut**  **Chi** | **HCE**  **p-value** | **HCE**  **Chi** | **VarEp**  **p-value** | **VarEp**  **Chi** |
| *regulation of transcription, DNA-templated* | **VarAut** | *0.0143 | 7.2396 | NA | NA | NA | NA | NA | NA |
| “ | **HCE** | **<0.0001 | 29.3811 | **0.0034 | 10.7671 | NA | NA | NA | NA |
| “ | **VarEp** | *0.0406 | 5.0671 | 1 | 0.000 | **0.0039 | 10.0271 | NA | NA |
| “ | **ID Only** | **0.0017 | 12.8188 | 0.2367 | 1.5842 | 0.1233 | 2.9425 | 0.2367 | 1.5506 |
|  |  | **HCA**  **p-value** | **HCA**  **Chi** | **VarAut**  **p-value** | **VarAut**  **Chi** | **HCE**  **p-value** | **HCE**  **Chi** | **VarEp**  **p-value** | **VarEp**  **Chi** |
| *synaptic transmission* | **VarAut** | 1 | 0.0079 | NA | NA | NA | NA | NA | NA |
| “ | **HCE** | 1 | 0.000 | 1 | 0.0506 | NA | NA | NA | NA |
| “ | **VarEp** | 0.7827 | 0.05885 | 0.5431 | 1.5225 | 0.7827 | 0.5229 | NA | NA |
| “ | **ID Only** | 0.5075 | 2.1369 | 0.5075 | 3.6634 | 0.5075 | 2.0497 | 1 | 0.1472 |

| **GO Molecular Function** |  | **HCA**  **p-value** | **HCA**  **Chi** | **VarAut**  **p-value** | **VarAut**  **Chi** | **HCE**  **p-value** | **HCE**  **Chi** | **VarEp**  **p-value** | **VarEp**  **Chi** |
| --- | --- | --- | --- | --- | --- | --- | --- | --- | --- |
| *catalytic activity* | **VarAut** | 0.2218 | 3.3919 | NA | NA | NA | NA | NA | NA |
| “ | **HCE** | *0.0416 | 8.2144 | 0.4463 | 1.4839 | NA | NA | NA | NA |
| “ | **VarEp** | 0.5213 | 0.821 | 0.6102 | 0.4805 | 0.2218 | 3.3663 | NA | NA |
| “ | **ID Only** | 0.4905 | 1.0999 | 0.6739 | 0.2653 | 0.2402 | 2.7693 | 1 | 0.000 |
|  |  | **HCA**  **p-value** | **HCA**  **Chi** | **VarAut**  **p-value** | **VarAut**  **Chi** | **HCE**  **p-value** | **HCE**  **Chi** | **VarEp**  **p-value** | **VarEp**  **Chi** |
| *chromatin binding* | **VarAut** | *0.0146 | 8.1225 | NA | NA | NA | NA | NA | NA |
| “ | **HCE** | **0.0011 | 14.902 | 0.2918 | 2.1147 | NA | NA | NA | NA |
| “ | **VarEp** | *0.0326 | 6.1627 | 1 | 0.000 | 0.311 | 1.5194 | NA | NA |
| “ | **ID Only** | **0.0018 | 12.767 | 0.311 | 1.5773 | 1 | 0.000 | 0.3662 | 1.1058 |
|  |  | **HCA**  **p-value** | **HCA**  **Chi** | **VarAut**  **p-value** | **VarAut**  **Chi** | **HCE**  **p-value** | **HCE**  **Chi** | **VarEp**  **p-value** | **VarEp**  **Chi** |
| *DNA binding* | **VarAut** | 0.0948 | 4.8014 | NA | NA | NA | NA | NA | NA |
| “ | **HCE** | *0.0353 | 8.5132 | 0.7222 | 0.834 | NA | NA | NA | NA |
| “ | **VarEp** | 0.1179 | 3.9403 | 1 | 0.000 | 0.802 | 0.4961 | NA | NA |
| “ | **ID Only** | 0.0598 | 6.3157 | 0.846 | 0.2869 | 1 | 0.00074 | 0.8933 | 0.1337 |

| **GO Cellular Component** |  | **HCA**  **p-value** | **HCA**  **Chi** | **VarAut**  **p-value** | **VarAut**  **Chi** | **HCE**  **p-value** | **HCE**  **Chi** | **VarEp**  **p-value** | **VarEp**  **Chi** |
| --- | --- | --- | --- | --- | --- | --- | --- | --- | --- |
| *axon* | **VarAut** | 0.7954 | 0.0672 | NA | NA | NA | NA | NA | NA |
| “ | **HCE** | *0.0302 | 6.6244 | *0.0185 | 9.4233 | NA | NA | NA | NA |
| “ | **VarEp** | 0.4984 | 0.5885 | 0.2095 | 1.9463 | 0.1891 | 2.6272 | NA | NA |
| “ | **ID Only** | *0.037 | 5.757 | *0.0185 | 8.231 | NA | NA | 0.2029 | 2.2308 |
|  |  | **HCA**  **p-value** | **HCA**  **Chi** | **VarAut**  **p-value** | **VarAut**  **Chi** | **HCE**  **p-value** | **HCE**  **Chi** | **VarEp**  **p-value** | **VarEp**  **Chi** |
| *cell projection* | **VarAut** | 0.0713 | 4.7961 | NA | NA | NA | NA | NA | NA |
| “ | **HCE** | 0.1266 | 3.4486 | **0.0001 | 19.1335 | NA | NA | NA | NA |
| “ | **VarEp** | 0.2472 | 1.8565 | **0.0005 | 14.5658 | 1 | 0.0404 | NA | NA |
| “ | **ID Only** | 0.2014 | 2.4065 | **0.0004 | 15.6763 | 1 | 0.000 | 1 | 0.000 |
|  |  | **HCA**  **p-value** | **HCA**  **Chi** | **VarAut**  **p-value** | **VarAut**  **Chi** | **HCE**  **p-value** | **HCE**  **Chi** | **VarEp**  **p-value** | **VarEp**  **Chi** |
| *chromosome* | **VarAut** | 0.1345 | 3.3484 | NA | NA | NA | NA | NA | NA |
| “ | **HCE** | *0.0392 | 8.3217 | 0.3477 | 1.5812 | NA | NA | NA | NA |
| “ | **VarEp** | 0.5571 | 0.4519 | 0.5317 | 0.6355 | 0.1345 | 4.1859 | NA | NA |
| “ | **ID Only** | *0.0416 | 6.9617 | 0.4092 | 1.1363 | 1 | 0.000 | 0.1345 | 3.3605 |
|  |  | **HCA**  **p-value** | **HCA**  **Chi** | **VarAut**  **p-value** | **VarAut**  **Chi** | **HCE**  **p-value** | **HCE**  **Chi** | **VarEp**  **p-value** | **VarEp**  **Chi** |
| *cytosol* | **VarAut** | 0.3955 | 2.4351 | NA | NA | NA | NA | NA | NA |
| “ | **HCE** | 1 | 0.000 | 0.3955 | 2.5286 | NA | NA | NA | NA |
| “ | **VarEp** | 0.7278 | 0.4352 | 0.1173 | 6.3518 | 0.7238 | 0.6114 | NA | NA |
| “ | **ID Only** | 0.8402 | 0.1249 | 0.6298 | 1.0101 | 0.8402 | 0.0964 | 0.5504 | 1.5034 |
|  |  | **HCA**  **p-value** | **HCA**  **Chi** | **VarAut**  **p-value** | **VarAut**  **Chi** | **HCE**  **p-value** | **HCE**  **Chi** | **VarEp**  **p-value** | **VarEp**  **Chi** |
| *endomembrane system* | **VarAut** | 1 | 0.000 | NA | NA | NA | NA | NA | NA |
| “ | **HCE** | 0.2144 | 2.3092 | 0.1745 | 2.9245 | NA | NA | NA | NA |
| “ | **VarEp** | 0.0879 | 4.9325 | 0.0879 | 6.3688 | 0.762 | 0.3879 | NA | NA |
| “ | **ID Only** | 0.1127 | 4.0151 | 0.0879 | 5.1295 | 0.8634 | 0.1583 | 1 | 0.002 |
|  |  | **HCA**  **p-value** | **HCA**  **Chi** | **VarAut**  **p-value** | **VarAut**  **Chi** | **HCE**  **p-value** | **HCE**  **Chi** | **VarEp**  **p-value** | **VarEp**  **Chi** |
| *Golgi apparatus* | **VarAut** | 1 | 0.000 | NA | NA | NA | NA | NA | NA |
| “ | **HCE** | 0.1539 | 3.4948 | 0.0612 | 6.2769 | NA | NA | NA | NA |
| “ | **VarEp** | 0.5338 | 0.6309 | 0.3805 | 1.4516 | 0.5307 | 0.7987 | NA | NA |
| “ | **ID Only** | 0.0867 | 4.9563 | *0.0355 | 8.5026 | 0.8867 | 0.0655 | 0.3805 | 1.6642 |
|  |  | **HCA**  **p-value** | **HCA**  **Chi** | **VarAut**  **p-value** | **VarAut**  **Chi** | **HCE**  **p-value** | **HCE**  **Chi** | **VarEp**  **p-value** | **VarEp**  **Chi** |
| *Golgi membrane* | **VarAut** | 1 | 0.0024 | NA | NA | NA | NA | NA | NA |
| “ | **HCE** | 1 | 0.000 | 1 | 0.000 | NA | NA | NA | NA |
| “ | **VarEp** | 0.6001 | 0.9476 | 0.6001 | 0.6502 | 0.6001 | 0.7047 | NA | NA |
| “ | **ID Only** | *0.0298 | 6.837 | *0.0298 | 8.3841 | *0.0298 | 6.9717 | 0.3132 | 2.35 |
|  |  | **HCA**  **p-value** | **HCA**  **Chi** | **VarAut**  **p-value** | **VarAut**  **Chi** | **HCE**  **p-value** | **HCE**  **Chi** | **VarEp**  **p-value** | **VarEp**  **Chi** |
| *neuron part* | **VarAut** | 0.6923 | 0.2416 | NA | NA | NA | NA | NA | NA |
| “ | **HCE** | **0.0052 | 9.4654 | **0.0004 | 15.3438 | NA | NA | NA | NA |
| “ | **VarEp** | 0.1253 | 3.1665 | *0.0175 | 6.8766 | 0.3507 | 1.1644 | NA | NA |
| “ | **ID Only** | **0.0023 | 11.5458 | *0.0004 | 17.0688 | 0.7944 | 0.0679 | 0.1588 | 2.5375 |
|  |  | **HCA**  **p-value** | **HCA**  **Chi** | **VarAut**  **p-value** | **VarAut**  **Chi** | **HCE**  **p-value** | **HCE**  **Chi** | **VarEp**  **p-value** | **VarEp**  **Chi** |
| *neuron projection* | **VarAut** | 0.6747 | 0.1761 | NA | NA | NA | NA | NA | NA |
| “ | **HCE** | **0.0004 | 14.6659 | **0.0001 | 19.5426 | NA | NA | NA | NA |
| “ | **VarEp** | 0.0687 | 3.7852 | *0.0124 | 7.3081 | 0.0687 | 3.7312 | NA | NA |
| “ | **ID Only** | **0.0007 | 12.9018 | **0.0001 | 17.2445 | NA | NA | 0.0828 | 3.2014 |
|  |  | **HCA**  **p-value** | **HCA**  **Chi** | **VarAut**  **p-value** | **VarAut**  **Chi** | **HCE**  **p-value** | **HCE**  **Chi** | **VarEp**  **p-value** | **VarEp**  **Chi** |
| *nucleus* | **VarAut** | **0.0009 | 12.7956 | NA | NA | NA | NA | NA | NA |
| “ | **HCE** | **0.0002 | 18.4975 | 0.5947 | 1.086 | NA | NA | NA | NA |
| “ | **VarEp** | **0.0004 | 15.5932 | 0.7932 | 0.5082 | 0.9176 | 0.0107 | NA | NA |
| “ | **ID Only** | **0.0009 | 13.3464 | 0.8725 | 0.1505 | 0.8725 | 0.1583 | 0.9176 | 0.0141 |
|  |  | **HCA**  **p-value** | **HCA**  **Chi** | **VarAut**  **p-value** | **VarAut**  **Chi** | **HCE**  **p-value** | **HCE**  **Chi** | **VarEp**  **p-value** | **VarEp**  **Chi** |
| *somatodendritic compartment* | **VarAut** | 1 | 0.027 | NA | NA | NA | NA | NA | NA |
| “ | **HCE** | 0.0744 | 4.8137 | 0.0664 | 7.3674 | NA | NA | NA | NA |
| “ | **VarEp** | 0.1637 | 2.7343 | 0.0744 | 4.7239 | 1 | 0.0126 | NA | NA |
| “ | **ID Only** | 0.0962 | 3.9068 | 0.068 | 6.0895 | 1 | 0.000 | 1 | 0.000 |
|  |  | **HCA**  **p-value** | **HCA**  **Chi** | **VarAut**  **p-value** | **VarAut**  **Chi** | **HCE**  **p-value** | **HCE**  **Chi** | **VarEp**  **p-value** | **VarEp**  **Chi** |
| *synapse* | **VarAut** | 0.4464 | 1.8095 | NA | NA | NA | NA | NA |  |
| “ | **HCE** | 1 | 0.0775 | 0.2465 | 4.2413 | NA | NA | NA | NA |
| “ | **VarEp** | 1 | 0.0158 | 0.2465 | 3.3996 | 1 | 0.000 | NA | NA |
| “ | **ID Only** | 1 | 0.0072 | 0.2465 | 3.1934 | 1 | 0.000 | 1 | 0.000 |

------------------------------------------------------------------------------------------------------------------------------------------------------------------

**Analysis Series 2.** Comparison of GO gene functions in dominant versus recessive patterns of inheritance in both the HCE and HCA groups. The *prop.test()* function in the statistical computing software, *R*, was used for statistical analyses. All pairs of proportions were compared using a Chi-square test of two proportions with one degree of freedom. P-values are unadjusted due to lack of multiple comparisons.

**HCE Dominant, N = 19**

**HCE Recessive, N = 63**

| **Analysis** | **Biological Process** | **p-value** | **Chi** |
| --- | --- | --- | --- |
| **HCE Dominant x HCE Recessive** | *lipid metabolic process* | 0.0561 | 3.65 |
| **“** | *carbohydrate derivative metabolic process* | 0.2908 | 1.12 |
| **“** | *membrane lipid metabolic process* | 0.1461 | 2.11 |
| **“** | *cellular amino acid metabolic process* | 0.1461 | 2.11 |

| **Analysis** | **Molecular Function** | **p-value** | **Chi** |
| --- | --- | --- | --- |
| **HCE Dominant x HCE Recessive** | *structural constituent of cytoskeleton* | **0.0018 | 9.77 |
| **“** | *potassium ion transmembrane transporter activity* | **0.0018 | 9.77 |
| **“** | *transferase* | 0.0783 | 3.10 |

| **Analysis** | **Cellular Component** | **p-value** | **Chi** |
| --- | --- | --- | --- |
| **HCE Dominant x HCE Recessive** | *endomembrane system* | 0.1829 | 1.77 |
| **“** | *endoplasmic reticulum* | 0.0913 | 2.85 |
| **“** | *endoplasmic reticulum membrane* | 0.0913 | 2.85 |
| **“** | *transmembrane transporter complex* | **0.0003 | 13.36 |
| **“** | *protein complex* | **<0.0001 | 16.83 |
| **“** | *myelin sheath* | **0.007 | 7.27 |

**HCA Dominant, N = 33**

**HCA Recessive, N = 23**

| **Analysis** | **Biological Process** | **p-value** | **Chi** |
| --- | --- | --- | --- |
| **HCA Dominant x HCA Recessive** | *anatomical structure development* | *0.0340 | 4.50 |
| **“** | *regulation of gene expression* | **0.0033 | 8.65 |
| **“** | *nervous system development* | *0.0126 | 6.22 |
| **“** | *regulation of transcription, DNA-templated* | **0.0048 | 7.96 |
| **“** | *cell differentiation* | *0.0395 | 4.24 |
| **“** | *neurogenesis* | 0.1426 | 2.15 |
| **“** | *chromosome organization* | *0.0232 | 5.15 |
| **“** | *regulation of synapse structure or activity* | 0.2588 | 1.28 |

| **Analysis** | **Molecular Function** | **p-value** | **Chi** |
| --- | --- | --- | --- |
| **HCA Dominant x HCA Recessive** | *nucleic acid binding* | **0.0092 | 6.79 |
| **“** | *DNA binding* | *0.0206 | 5.36 |
| **“** | *chromatin binding* | *0.0247 | 5.04 |

| **Analysis** | **Cellular Component** | **p-value** | **Chi** |
| --- | --- | --- | --- |
| **HCA Dominant x HCA Recessive** | *membrane-bounded organelle* | **0.0076 | 7.12 |
| **“** | *neuron part* | 0.1032 | 2.66 |
| **“** | *neuron projection* | *0.0391 | 4.26 |

------------------------------------------------------------------------------------------------------------------------------------------------------------------

**Analysis 3.** Comparison of the number of nuclear epigenetic regulators according to dominant and recessive subgroups in HCA and HCE. Performed with a proportions comparison. “Dom” = dominant; “Rec” = recessive.

| **Analysis** | **Z** | **p-value** | **Diff** |
| --- | --- | --- | --- |
| **HCA Dom x HCA Rec** | 3.5 | **0.0005 | 0.2045, 0.7355 |
| **HCE Dom x HCE Rec** | 0.4 | 0.6835 | -0.1142, 0.1742 |

------------------------------------------------------------------------------------------------------------------------------------------------------------------

**Analysis 4.** Comparison of the ratios of dominant/recessive conditions in HCA versus HCE. Dominant conditions were assigned a value of “1” while recessive conditions were assigned “2”. A two-tailed heteroscedastic T-test was used.

| **Group** | **N** | **Mean** | **SD** | **p-value** |
| --- | --- | --- | --- | --- |
| **HCA** | 56 | 1.4107 | 0.4964 | -- |
| **HCE** | 82 | 1.7683 | 0.4245 | -- |
| **Total** | 138 | -- | -- | **< 0.0001 |

------------------------------------------------------------------------------------------------------------------------------------------------------------------

**Analysis Series 5.** Comparison of the number of gene products that act as nuclear epigenetic regulators according to group. The *prop.test()* function in the statistical computing software, *R*, was used for the statistical analyses. All pairs of proportions were compared using a Chi-square test of two proportions with one degree of freedom. A false discovery rate adjustment was applied to account for multiple comparisons.

**HCA, N = 71**

**VarAut, N = 124**

**HCE, N = 86**

**VarEp, N = 78**

**ID Only, N = 75**

| **Epigenetic Regulators** |  | **HCA**  **p-value** | **HCA**  **Chi** | **VarAut**  **p-value** | **VarAut**  **Chi** | **HCE**  **p-value** | **HCE**  **Chi** | **VarEp**  **p-value** | **VarEp**  **Chi** |
| --- | --- | --- | --- | --- | --- | --- | --- | --- | --- |
|  | **VarAut** | **0.0004 | 18.2671 | NA | NA | NA | NA | NA | NA |
|  | **HCE** | **<0.0001 | 34.9546 | *0.0316 | 5.8231 | NA | NA | NA | NA |
|  | **VarEp** | **0.0004 | 18.2768 | 0.7767 | 0.244 | 0.1584 | 2.5412 | NA | NA |
|  | **ID Only** | **0.0004 | 16.942 | 0.803 | 0.1259 | 0.1494 | 2.8805 | 1 | 0 |

------------------------------------------------------------------------------------------------------------------------------------------------------------------

**Analysis Series 6.** Comparison of the number of gene products that act as nuclear epigenetic regulators in the SFARI HCA set versus those HCA genes low-ranked or not included in SFARI (HCA Non-SFARI). A 2-tailed proportions comparison was used.

**Full HCA, N = 32**

**SFARI-only HCA, N = 39**

| **Analysis** | **Z** | **p-value** | **Diff** |
| --- | --- | --- | --- |
| **HCA SFARI Only x HCA Non-SFARI** | 0.8 | 0.4501 | -0.1436, 0.3236 |

------------------------------------------------------------------------------------------------------------------------------------------------------------------

**Analysis Series 7.** Comparison of the number of genes that connects with the core protein-protein (PPI) network according to String 10. The *prop.test()* function in the statistical computing software, *R*, was used for the statistical analyses. All pairs of proportions were compared using a Chi-square test of two proportions with one degree of freedom. A false discovery rate adjustment was applied to account for multiple comparisons.

**HCA, N = 71**

**VarAut, N = 124**

**HCE, N = 86**

**VarEp, N = 78**

**ID Only, N = 74**

| **Protein-protein Interaction** |  | **HCA**  **p-value** | **HCA**  **Chi** | **VarAut**  **p-value** | **VarAut**  **Chi** | **HCE**  **p-value** | **HCE**  **Chi** | **VarEp**  **p-value** | **VarEp**  **Chi** |
| --- | --- | --- | --- | --- | --- | --- | --- | --- | --- |
| *Confidence* | **VarAut** | 0.1053 | 2.981 | NA | NA | NA | NA | NA | NA |
| “ | **HCE** | 0.9151 | 0.0114 | *0.0432 | 4.6937 | NA | NA | NA | NA |
| “ | **VarEp** | **0.0011 | 11.9996 | **<0.0001 | 35.1987 | **0.0012 | 11.4692 | NA | NA |
| “ | **ID Only** | **0.0003 | 15.5409 | **<0.0001 | 41.2973 | **0.0003 | 15.1194 | 0.7687 | 0.1571 |

------------------------------------------------------------------------------------------------------------------------------------------------------------------

**Analysis Series 8.** Comparison of the number of intermediary nodes that lay between a target protein and its closest core protein neighbor according to String 10. Derived from the Confidence (not Action) network. Analyzed using an ANOVA. Bonferroni correction for multiple (10) comparisons. (α = 0.005.) First table displays group means and standard deviations (SD). Second table displays analyses and unadjusted p-values. All p-values <0.005 are considered significant.

| **Group** | **N** | **Mean** | **SD** |
| --- | --- | --- | --- |
| **HCA** | 51 | 0.9216 | 0.9556 |
| **VarAut** | 103 | 1.0000 | 0.7541 |
| **HCE** | 60 | 3.2167 | 2.0260 |
| **VarEp** | 33 | 0.5455 | 0.7942 |
| **ID Only** | 28 | 0.5000 | 0.5774 |

| **Analysis** | **unadjusted p-value** |
| --- | --- |
| **HCA x VarAut** | 0.6098 |
| **HCA x HCE** | **<0.0001 |
| **HCA x VarEp** | 0.0543 |
| **HCA x ID Only** | 0.0170 |
| **VarAut x HCE** | **<0.0001 |
| **VarAut x VarEp** | 0.0006 |
| **VarAut x ID Only** | *0.0004 |
| **HCE x VarEp** | **<0.0001 |
| **HCE x ID Only** | **<0.0001 |
| **VarEp x ID Only** | 0.7973 |

------------------------------------------------------------------------------------------------------------------------------------------------------------------

**Analysis Series 9.** Comparison of the number of neurodegenerative disorders in HCE versus VarEp. A 2-tailed proportions test was used.

| **Analysis** | **Z** | **p-value** | **Diff** |
| --- | --- | --- | --- |
| **HCE x VarEp** | 2.5 | *0.0119 | 0.0375, 0.3025 |
