# Supplementary material for: Genes with high penetrance for syndromic and non-syndromic autism typically function within the nucleus and regulate gene expression
Source: Mol Autism. 2016 Mar 15;7:18. doi: 10.1186/s13229-016-0082-z (PMC4793536; doi:10.1186/s13229-016-0082-z)
Supplement: Additional file 3: — Full statistical results. (DOCX 157 kb) [file 13229_2016_82_MOESM3_ESM.docx]

**ADDITIONAL FILE 3 - REFERENCES FOR ADDITIONAL FILE 1:**

Abdollahi MR, Morrison E, Sirey T, Molnar Z, Hayward BE, Carr IM, et al. 2009. Mutation of the variant alpha-tubulin TUBA8 results in polymicrogyria with optic nerve hypoplasia. *Am J Hum Genet* **85**: 737-744.

Abou Jamra R, Wohlfart S, Zweier M, Uebe S, Priebe L, Ekici A, et al. 2011. Homozygosity mapping in 64 Syrian consanguineous families with non-specific intellectual disability reveals 11 novel loci and high heterogeneity. *Eur J Hum Genet* **19**: 1161-1166.

Adachi M, Abe Y, Aoki Y, and Matsubara Y. 2012. Epilepsy in RAS/MAPK syndrome: two cases of cardio-facio-cutaneous syndrome with epileptic encephalopathy and a literature review. *Seizure* **21**: 55-60.

Adam MP, Hudgins L. 2005. Kabuki syndrome: a review. *Clin Genet* **67**: 209-219.

Agha Z, Iqbal Z, Azam M, Willemsen MH, Kleefstra T, Zweier C, de Leeuw N, Qamar R, van Bokhoven H. 2014. A complex microcephaly syndrome in a Pakistani family associated with a novel missense mutation in RBBP8 and a heterozygous deletion in NRXN1. *Gene* **538**: 30-35*.*

Akizu N, Shembesh NM, Ben-Omran T, Bastaki L, Al-Tawari A, Zaki MS, Koul R, Spencer E, Rosti RO, Scott E, Nickerson E, et al. 2013. Whole-exome sequencing identifies mutated c12orf57 in recessive corpus callosum hypoplasia. *Am J Hum Genet* **92**: 392-400.

Alazami AM, Adly N, Al Dhalaan H, Alkuraya FS. 2011. A nullimorphic ERLIN2 mutation defines a complicated hereditary spastic paraplegia locus (SPG18). *Neurogenetics* **12**: 333-336.

Aldahmesh MA, Mohamed JY, Alkuraya HS, Verma IC, Puri RD, Alaiya AA, et al. 2011. Recessive mutations in ELOVL4 cause ichthyosis, intellectual disability, and spastic quadriplegia. *Am J Hum Genet* **89**: 745-750.

Alesi V, Bertoli M, Barrano G, Torres B, Pusceddu S, Pastorino M, Perria C, Nardone A M, Novelli A, Serra G. 2012. 335.4 kb microduplication in chromosome band Xp11.2p11.3 associated with developmental delay, growth retardation, autistic disorder and dysmorphic features. *Gene* **505**: 384-387.

al-Hemidan AI, al-Hazzaa SA. 1995. Richner-Hanhart syndrome (tyrosinemia type II). Case report and literature review. *Ophthalmic Genet* **16**: 21-26.

Al-Kateb H, Shimony JS, Vineyard M, Manwaring L, Kulkarni S, Shinawi M. 2013. NR2F1 haploinsufficiency is associated with optic atrophy, dysmorphism and global developmental delay. *Am J Med Genet* **161A:** 377-381.

Alkuraya FS. 2010. Mental retardation, growth retardation, unusual nose, and open mouth: an autosomal recessive entity. *Am J Med Genet A* **152A**: 2160-2163.

Alkuraya FS, Cai X, Emery C, Mochida GH, Al-Dosari MS, Felie JM, et al. 2011. Human mutations in NDE1 cause extreme microcephaly with lissencephaly. *Am J Hum Genet* **88**: 536-547.

Al-Sarraj Y, Ben-Omran T, Tolefat M, Bejaoui Y, El-Shanti H, Kambouris M. 2014. A novel missense mutation in SRD5A3 causes congenital disorder of glycosylation type I (cerebello-ocular syndrome). *J Inborn Errors Metab Scr* **2**: 2326409814550528.

Andria A, Strisciuglio P, Pontarelli G, Sly WS, Dodson WE. 1981. Infantile neuraminidase and beta-galactosidase deficiencies (galactosialidosis) with mild clinical courses. In: *Perspectives in Inherited Metabolic Diseases*. Milan Ermes (pub.) 4: pp. 379-395.

Ardinger HH, Hanson JW, Zellweger HU. 1984. Börjeson-Forssman-Lehman syndrome: further delineation in five cases. *Am J Med Genet* **19**: 653-664.

Armour CM, Allanson JE. 2008. Further delineation of cario-facio-cutaneous syndrome: clinical features of 38 individuals with proven mutations. *J Med Genet* **45**: 249-254.

Arvio M, Oksanen V, Autio S, Gaily E, Sainio K. 1993. Epileptic seizures in aspartylgucosaminuria: a common disorder. *Acta Neurol Scand* **87**: 342-344.

Aula N, Salomäki P, Timonen R, Verheijen F, Mancini G, Månsson JE, Aula P, Peltonen L. 2000. The spectrum of SLC17A5-gene mutations resulting in free sialic acid-storage diseases indicates some genotype-phenotype correlation. *Am J Hum Genet* **67**: 832-840.

Babu P, Sharma R, Jayaseelan E, Appachu D. (2008). Berardinelli-Seip syndrome in a 6-year-old boy. *Indian J Dermatol Venereol Leprol* **74**: 644-646.

Bachmann-Gagescu R, Ishak GE, Dempsey JC, Adkins J, O’Day D, Phelps IG, et al. 2012. Genotype-phenotype correlation in CC2D2A-related Joubert syndrome reveals an association with ventriculomegaly and seizures. *J Med Genet* **49**: 126-137.

Bakircioglu M, Carvalho OP, Khurshid M, Cox JJ, Tuysuz B, Barak T, et al. 2011. The essential role of centrosomal NDE1 in human cerebral cortex neurogenesis. *Am J Hum Genet* **88**: 523-535.

Bakker HD, de Sonnaville ML, Vreken P, Abeling NG, Groener JE, Keulemans JL, et al. 2001. Human alpha-N-acetylgalactosaminidase (alpha-NAGA) deficiency: no association with neuroaxonal dystrophy? *Eur J Hum Genet* **9**: 91-96.

Balasubramanian M, Smith K, Basel-Vanagaite L, Feingold MF, Brock P, Gowans GC, et al. 2011. Case series: 2q33.1 microdeletion syndrome—further delineation of the phenotype. *J Med Genet* **48**: 290-298.

Banka S, Blom HJ, Walter J, Aziz M, Urquhart J, Clouthier CM, et al. 2011. Identification and characterization of an inborn error of metabolism caused by dihydrofolate reductase deficiency. *Am J Hum Genet* **88**: 216-225.

Baple EL, Maroofian R, Chioza BA, Izadi M, Cross HE, Al-Turkis S, et al. 2014. Mutations in KPTN cause macrocephaly, neurodevelopmental delay, and seizures. *Am J Hum Genet* **94**: 87-94.

Barcia G, Chemaly N, Gobin S, Milh M, Van Bogaert P, Barnerias C, Kaminska A, Dulac O, Desguerre I, Cormier V, et al. 2014. Early epileptic encephalopathies associated with STXBP1 mutations: Could we better delineate the phenotype? *Eur J Med Genet* ***57***: 15-20.

Barten S, Proesmans W. 1996. Alport syndrome: Clinical experience with 21 paediatric patients. *Eur J Pediatri* **155**: 49-52.

Basel-Vanagaite L, Dallapiccola B, Ramirez-Solis R, Segref A, Thiele H, Edwards A, Arends MJ, Miró X, White JK, Désir J, et al. 2012. Deficiency for the ubiquitin ligase UBE3B in a blepharophimosis-ptosis-intellectual-disability syndrome. *Am J Hum Genet* **91**: 998-1010.

Basel-Vanagaite L, Hershkovitz T, Heyman E, Raspall-Chaure, Kakar N, Smirin-Yosef P, et al. 2013. Biallelic SZT2 mutations cause infantile encephalopathy with epilepsy and dysmorphic corpus callosum. *Am J Hum Genet* **93**: 524-529.

Bassi MT, Bresolin N, Tonelli A, Nazos K, Crippa F, Baschirotto C, et al. 2004. A novel mutation in the ATP1A2 gene causes alternating hemiplegia of childhood. *J Med Genet* **41**: 621-628.

Bauer P, Leshinsky-Silver E, Blumkin L, Schlipf N, Schröder C, Schicks J, Lev D, Riess O, Lerman-Sagie T, Schöls L. 2012. Mutation in the AP4B1 gene cause hereditary spastic paraplegia type 47 (SPG47). *Neurogenetics* **13**: 73-76.

Belet S, Fieremans N, Yuan X, Van Esch H, Verbeeck J, Ye Z, et al. 2014. Early frameshift mutation in PIGA identified in a large XLID family without neonatal lethality. *Hum Mutat* **35**: 350-355.

Bem D, Yoshimura S, Nunes-Bastos R, Bond FC, Kurian MA, Rahman F, et al. 2011. Loss-of-function mutations in RAB18 cause Warburg micro syndrome. *Am J Hum Genet* **88**: 499-507.

Ben-Zeev, B, Hoffman C, Lev D, Watemberg N, Malinger G, Brand N, et al. 2003. Progresive cerebellocerebral atrophy: a new syndrome with microcephaly, mental retardation, and spastic quadriplegia. *J Med Genet* **40**: e96.

Berard-Badier M, Adechy-Benkoel L, Chamlian A, Dubois-Gambarelli D, Casanova P, Mariani A. 1970. Etude ultrastructurale du parenchyme hepatique dans les mucopolysaccharidoses. *Path Biol* **18**: 117-128.

Berryer MH, Hamdan FF, Klitten LL, Møller RS, Carmant L, Schwartzentruber J, et al. 2013. Mutations in SYNGAP1 cause intellectual disability, autism, and a specific form of epilepsy by inducing haploinsufficiency. *Hum Mutat* **34**: 385-394.

Beunders G, Voorhoeve E, Golzio C, Pardo LM, Rosenfeld JA, Talkowski ME, et al. 2013. Exonic deletions in AUTS2 cause a syndromic form of intellectual disability and suggest a critical role for the C terminus. *Am J Hum Genet* **92**: 210-220.

Biancheri R, Bertini E, Falace A, Pedemonte M, Rossi A, D’Amico A, Scapolan S, Bergamino L, Petrini S, Cassandrini D, et al. 2006. POMGnT1 mutations in congenital muscular dystrophy: genotype-phenotype correlation and expanded clinical spectrum. *Arch Neurol* **63**: 1491-1495.

Biancheri R, Zara F, Bruno C, Rossi A, Bordo L, Gazzerro E, et al. 2007. Phenotypic characterization of hypomyelination and congenital cataract. *Ann Neurol* **62**: 121-127.

Bicknell LS, Pitt J, Aftimos S, Ramadas R, Maw MA, Robertson SP. 2008. A missense mutation in the ALDH18A1, encoding Delta1-pyrroline-5-carboxylate syndrome (P5CS), causes an autosomal recessive neurocutaneous syndrome. *Eur J Hum Genet* **16**: 1176-1186.

Bienvenu T, des Portes V, Saint Martin A, McDonnell N, Billuart P, Carrié A, Vinet MC, Couvert P, Toniolo D, Ropers HH, et al. 1998. Non-specific X-linked semidominant mental retardation by mutations in a Rab GDP-dissociation inhibitor. *Hum Mol Gene* **7**: 1311-1315.

Bilgüvar K, Oztürk AK, Louvi A, Kwan KY, Choi M, Tatli B, et al. 2010. Whole-exome sequencing identifies recessive WDR62 mutations in severe brain malformations. *Nature* **467**: 207-210.

Bindu PS, Shehanaz KE, Christopher R, Pal PK, Ravishankar S. 2007. Intermediate maple syrup urine disease: neuroimaging observations in 3 patients from South India. *J Child Neurol* **22**: 911-913.

Bjursell MK, Blom HJ, Cayuela JA, Engvall ML, Lesko N, Balasubramaniam S, et al. 2011. Adenosine kinase deficiency disrupts the methionine cycle and causes hypermethioninemia, encephalopathy, and abnormal liver function. *Am J Hum Genet* **89**: 507-515.

Blake KD, Salem-Hartshorne N, Daoud MA, Gradstein J. 2005. Adolescent and adult issues in CHARGE syndrome. *Clin Pediatr (Phila)* **44**: 151-159.

Boccuto L, Chen CF, Pittman AR, Skinner CD, McCartney HJ, Jones K, Bochner, BR, Stevenson RE, and Schwartz CE. 2013. Decreased tryptophan metabolism in patients with autism spectrum disorders. *Mol Autism* **4**: 16.

Bodamer OA, Giugliani R, Wood T. 2014. The laboratory diagnosis of mucopolysaccharidosis III (Sanfilippo syndrome): a changing landscape. *Mol Genet Metab* **113**: 34-41.

Bohring A, Silengo M, Lerone M, Superneau DW, Spaich C, Braddock SR, et al. 1999. Severe end of Opitz trigonocephaly (C) syndrome or new syndrome? *Am J Med Genet* **8**: 438-446.

Bonaglia MC, Ciccone R, Gimelli G, Gimelli S, Marelli S, Verheij J, Giorda R, Grasso R, Borgatti R, Pagone F, et al. 2008. Detailed phenotype-genotype study in five patients with chromosome 6q16 deletion: narrowing the critical region for Prader-Willi-like phenotype. *Eur J Hum Genet* **16**: 1443-1449.

Bonati MT, Russo S, Finelli P, Valsecchi MR, Cogliati F, Cavalleri F, Roberts W, Elia M, Larizza L. 2007. Evaluation of autism traits in Angelman syndrome: a resource to unfolding autism genes. *Neurogenetics* **8**: 169-178.

Borck G, Mollà-Herman A, Boddaert N, Encha-Razavi F, Philippe A, Robel L, Desguerre I, Brunelle F, Benmerah A, Munnich A, et al. 2008. Clinical, cellular, and neuropathological consequences of AP1S2 mutations: further delineation of a recognizable X-linked mental retardation syndrome. *Hum Mutat* **29**: 966-974.

Botta E, Nardo T, Orioli D, Guglielmino R, Ricotti R, Bondanza S, Benedicenti F., Zambruno G, Steganini M. 2009. Genotype-phenotype relationships in trichothiodystrophy patients with novel splicing mutations in the XPD gene. *Hum Mutat* **30**: 438-445.

Bowen P, Armstrong HB. 1976. Ectodermal dysplasia, mental retardation, cleft lip/palate and other anomalies in three sibs. *Clin Genet* **9**: 35-42.

Bowl MR, Nesbit MA, Harding B, Levy E, Jefferson A, Volpi E, Rizzoti K, Lovell-Badge R, Schlessinger D, Whyte, MP, et al. 2005. An interstitial deletion-insertion involving chromosomes 2p25.3 and Xq27.1, near SOX3, causes X-linked recessive hypoparathyroidism. *J Clin Invest* **115**: 2822-2831.

Budny B, Badura-Stronka M, Materna-Kiryluk A, Tzschach A, Raynaud M, Latos-Bielenska A, et al. 2010. Novel missense mutations in the ubiquitination-related gene UBE2A cause a recognizable X-linked mental retardation syndrome. *Clin Genet* **77**: 541-551.

Burd L, Stenehjem A, Franceschini LA, Kerbeshian J. 2000. A 15-year follow-up of a boy with pyridoxine (vitamin B6)-dependent seizures with autism, breath holding, and severe mental retardation. *J Child Neurol* **15**: 763-765.

Burlina AB, Burlina AP, Hyland K, Bonafe L, Blau N. 2001. Autistic syndrome and aromatic L-amino acid decarboxylase deficiency. *J Inhert Metab Dis* **24**: 34.

Butler MG, Hall BD, Maclean RN, Lozzi CB. 1987. Do some patients with Seckel syndrome have hematological problems and/or chromosome breakage? *Am J Med Genet* **27**: 645-649.

Cantani A, Gagliesi D. 1998. Rubinstein-Taybi syndrome. Review of 732 cases and analysis of the typical traits. *Eur Rev Med Pharmacol Sci* **2**: 81-87.

Capovilla G, Lorenzetti ME, Montagnin A, Borgatti R, Piccinelli P, Giordana L, et al. 2001. Seckel’s syndrome and malformations of cortical development: report of three new cases and review of the literature. *J Child Neurol*

Caputo V, Cianetti L, Niceta M, Carta C, Ciolfi A, Bocchinfuso G, Carrani E, Dentici ML, Biamino E, Belligni E, et al. 2012. A restricted spectrum of mutations in the SMAD4 tumor-suppressor gene underlies Myhre syndrome. *Am J Hum Genet* **90**: 161-169. **16**: 382-286.

Cario H, Smith DE, Blom H, Blau N, Bode H, Holzmann K, et al. 2011. Dhydrofolate reductase deficiency due to a homozygous DHFR mutation causes megaloblastic anemia and cerebral folate deficiency leading to severe neurologic disease. *Am J Hum Genet* **88**: 226-231.

Carrascosa-Romero MC, Suela J, Pardal-Fernández JM, Bermejo-Sánchez E, Vidal-Company A, MacDonald A, Tébar-Gil R, Martínez-Fernández ML, Martínez-Frías ML. 2013. A 2.84 Mb deletion at 21q22.11 in a patient clinically diagnosed with Marden-Walker syndrome. *Am J Med Genet* **161A**: 2281-2290.

Carss KJ, Stevens E, Foley AR, Cirak S, Riemersma M, Torelli S, et al. 2013. Mutations in GDP-mannose pyrophosphorylase B cause congenital and limb-girdle muscular dystrophies associated with hypoglycosylation of α-dystroglycan. *Am J Hum Genet* **93**: 29-41.

Carta C, Pantaleoni F, Bocchinfuso G, Stella L, Vasta I, Sarkozy A, Digilio C, Palleschi A, Pizzuti A, Grammatico P, et al. 2006. Germline missense mutations affecting KRAS isoform B are associated with a severe Noonan syndrome phenotype. *Am J Hum Genet* **79**, 129-135.

Carvalho-Neto Ad, Ono SE, Cardoso Gde M, Santos ML, Celidonio I. 2009. Oculocerebrorenal syndrome of Lowe: magnetic resonance imaging findings in the first six years of life. *Arq Neuropsiquiatr* **67**: 305-307.

Carvill GL, Heavin SB, Yendle SC, McMahon JM, O’Roak BJ, Cook J, et al. 2013. Targeted resequencing in epileptic encephalopathies identifies de novo mutations in CHD2 and SYNGAP1. *Nat Genet* **45**: 825-830.

Carvill Gl, Weckhuysen S, McMahon JM, Hartmann C, Møller RS, Hjalgrim H, et al. 2014. GABRA1 and STXBP1: novel genetic causes of Dravet syndrome. *Neurology* **82**: 1245-1253.

Castro Gago M, Rodriguez E, Ugarte J, Diaz Cardama I, Alonso A, Pena J. 1982. Agenesia hereditaria del cuerpo calloso: una nueva forma. *Rev Esp Pediat* **38**: 349-353.

Chakanovskis JE, Sutherland GR. 1971. The Smith-Lemli-Opitz syndrome in a profoundly retarded epileptic boy. *J Ment Defic Res* **15**: 153-162.

Chang BS, Piao X, Giannini C, Cascino GD, Scheffer I, Woods CG, Topcu M, Tezcan K, Bodell A, Leventer RJ, et al. 2004. Bilateral generalized polymicrogyria (BGP): A distinct syndrome of cortical malformation. *Neurology* **62**: 1722-1728.

Cheillan D, Joncquel-Chevalier Curt M, Briand G, Salomons GS, Mention-Mulliez K, Dobbelaere D, Cuisset JM, Lion-François L, Portes VD, Chabli A, et al. 2012. Screening for primary creatine deficiencies in French patients with unexplained neurological symptoms. *Orphanet J Rare Dis* **7**: 96.

Chen H. 2006. Roberts syndrome. in *Atlas of genetic diagnosis and counseling*, pp. 852-855. Springer-Verlag, New York, NY.

Chénier S, Yoon G, Argiropoulos B, Lauzon J, Laframboise R, Ahn JW, Ogilvie CM, Lionel AC, Marshall CR, Vaags AK, et al. 2014. CHD2 haploinsufficiency is associated with developmental delay, intellectual disability, epilepsy and neurobehavioural problems. *J Neurodev Disord* **6**: 9.

Chitayat D, Applegarth DA, Lewis J, Dimmick JE, McCormick AQ, Hall JG. 1988. Juvenile galactosialidosis in a white male: a new variant. *Am J Med Genet* **31**: 887-901.

Clifford S, Dissanayake C, Bui QM, Huggins R, Taylor AK, Loesch DZ. 2007. Autism spectrum phenotype in males and females with fragile X full mutation and permutation. *J Autism Dev Disord* **37**: 738-747.

Cobben JM, Weiss MM, van Dijk FS, De Reuver R, de Kruiff C, Pondaag W, Hennekam, R. C., and Yntema, H. G. (2014). A de novo mutation in ZMYND11, a candidate gene for 10p15.3 deletion syndrome, is associated with syndrome intellectual disability. *Eur J Med Genet* **57**: 636-638.

Coe BP, Witherspoon K, Rosenfeld JA, van Bon BW, Vulto-van Silfhout AT, Bosco P, et al. 2014. Refining analyses of copy number variation identifies specific genes associated with developmental delay. *Nat Genet* **46**: 1063-1071.

Cormier-Daire V, Amiel J, Vuillaumier-Barrot S, Tan J, Durand G, Munnich A, et al. 2000. Congenital disorders of glycosylation IIa cause growth retardation, mental retardation, and facial dysmorphism. *J Med Genet* **37**: 875-877.

Coutton C, Bidart M, Rendu J, Devillard F. Vieville G, Amblard F, Lopez G, Jouk PS, Satre V. 2013. 190-kb duplication in 1p36.11 inluding PIGV and ARID1A genes in a girl with intellectual disability and hexadactyly. *Clin Genet* **84**: 596-599.

Cullup T, Kho AL, Dionisi-Vici C, Brandmeier B, Smith F, Urry Z, et al. 2013. Recessive mutations in EPG5 cause Vici syndrome, a multisystem disorder with defective autophagy. *Nat Genet* **45**: 83-87.

Cushion TD, Paciorkowski AR, Pilz DT, Mullins JG, Seltzer LE, Marion RW, et al. 2014. De novo mutations in the beta-tubulin gene TUBB2A cause simplified gural patterning and infantile-onset epilepsy. *Am J Hum Genet* **94**: 634-641.

Cusmano-Ozog K, Manning MA, Hoyme HE. 2007. *Am J Med Genet C Semin Med Genet* **145C**: 393-398.

Dabell MP, Rosenfeld JA, Bader P, Escobar LF, El-Khechen D, Vallee SE, et al. 2013. Investigation of NRXN1 deletions: clinical and molecular characterization. *Am J Med Genet A* **161A**: 717-731.

Dancourt J, Vuillaumier-Barrot S, de Baulny HO, Sfaello I, Barnier A, le Bizec C, et al. 2006. A new intronic mutation in the DPM1 gene is associated with a milder form of CDG Ie in two French siblings. *Pediatr Res* **59**: 835-839.

Dansault A, David G, Schwartz C, Jaliffa C, Vieira V, de la Houssaye G, Bigot K, Catin F, Tattu L, Chopin C, et al. 2007. Three new PAX6 mutations including one causing an unusual ophthalmic phenotype associated with neurodevelopmental abnormalities. *Mol Vis* **13**: 511-523.

Davis LK, Meyer KJ, Rudd DS, Librant AL, Epping EA, Sheffield VC, Wassink, TH. 2008. Pax6 3’ deletion results in aniridia, autism and mental retardation. *Hum Genet* **123**: 371-378.

Day R, Beckett B, Donnai D, Fryer A, Heidenbald M, Howard P, Kerr B, Mansour, S, Maye U, McKee S, et al. 2008. A clinical and genetic study of the Say/Barber/Biesecker/Young-Simpson type of Ohdo syndrome. *Clin Genet* **74**: 434-444.

de Alencastro G, McCloskey DE, Kliemann SE, Maranduba CM, Pegg AE, Wang X, et al. 2008. New SMS mutation leads to a striking reduction in spermine synthase protein function and a severe form of Snyder-Robinson X-linked recessive mental retardation syndrome. *J Med Genet* **45**: 539-543.

de Barsy AM, Moens E, Dierckx L. 1968. Dwarfism, oligophrenia and degeneration of the elastic tissue in skin and cornea. A new syndrome? *Helv Paediatr Acta* **23**: 305-313.

de Cock P, Jaeken J. 2009. MGAT2 deficiency (CDG-IIa): the life of J. *Biochim Biophys Acta* **1792**: 844-846.

De Koning TJ, Duran M, Van Maldergem L, Pineda M, Dorland L, Gooskens R, et al. 2002. Congenital microcephaly and seizures due to 3-phosphoglycerate dehydrogenase deficiency: outcome of treatment with amino acids. *J Inherit Metab Dis* **25**: 119-125.

de Ligt J, Willemsen MH, van Bon BW, Kleefstra T, Yntema HG, Kroes T, et al. 2012. Diagnostic exome sequencing in persons with severe intellectual disability. *N Engl J Med* **367**: 1921-1929.

Descheemaeker MJ, Govers V, Vermeulen P, Fryns JP. 2006. Pervasive developmental disorders in Prader-Willi syndrome: the Leuven experience in 59 subjects and controls. *Am J Med Genet A* **140**: 1136-1142.

Descipio C, Schneider L, Young TL, Wasserman N, Yaeger D, Yaeger D, Lu F, Wheeler PG, Williams MS, Bason L, Jukofsky L, et al. 2005. Subtelomeric deletions of chromosome 6p: molecular and cytogenetic characterization of three new cases with phenotypic overlap with Ritscher-Schinzel (3C) syndrome. *Am J Med Genet A* **134A**: 3-11.

De Wit MC, de Coo IF, Julier C, Delépine M, Lequin MH, van de Laar I, Sibbles BJ, Bruining GJ, Mancini GM. 2006. Microcephaly and simplified gyral pattern of the brain associated with early onset insulin-dependent diabetes mellitus. *Neurogenetics* **I7I**: 259-263.

Dhar SU, del Gaudio D, German JR, Peters SU, Ou Z, Bader PI, et al. 2010. 22q deletion syndrome: clinical and molecular analysis using array CGH. *Am J Med Genet A* **152A**: 573-581.

Di Bartolomeo R, Polidori G, Piastra M, Viola L, Zampino G, Chiaretti A. 2003. Malignant hypertension and cerebral haemorrhage in Seckel syndrome. *Eur J Pediatr* **162**: 860-862.

Dimopoulou A, Fischer B, Gardeitchik T, Schröter P, Kayserili H, Schlack C, Li Y, Brum JM, Barisic I, Castori M, Spaich C, et al. 2013. Genotype-phenotype spectrum of PYRCR1-related autosomal recessive cutis laxa. *Mol Genet Metab* **110**: 352-361.

Drielsma A, Jalas C, Simonis N Désir J, Simanovsky N, Pirson I, et al. 2012. Two novel CCDC88C mutations confirm the role of DAPLE in autosomal recessive congenital hydrocephalus. *J Med Genet* **49**: 708-712.

Dunbar M, Jaggumantri S, Sargent M, Stockler-Ipsiroglu S, van Karnebeek CD. 2014. Treatment of X-linked creatine transporter (SLC6A8) deficiency: systematic review of the literature and three new cases. *Mol Genet Metab* **112**: 259-274.

Edvardson S, Baumann AM, Mühlenhoff M, Stephan O, Kuss AW, Shaag A, He L, Zenvirt S, Tanzi R, Gerardy-Schann R. 2013. West syndrome caused by ST3Gal-III deficiency. *Epilepsia* **54**: e24-27.

Edvardson S, Shaag A, Zenvirt S, Erlich Y, Hannon GJ, Shanske AL, Gomori JM, Ekstein J, Elpeleg O. 2010. Joubert syndrome 2 (JBTS2) in Ashkenazi Jews is associated with a TMEM216 mutation. *Am J Hum Genet* 86: 93-97.

Ekici AB, Hilfinger D, Jatzwauk M, Thiel CT, Wenzel D, Lorenz I, et al. 2010. Disturbed Wnt signaling due to a mutation in CCDC88C causes an autosomal recessive non-syndromic hydrocephalus with medial diverticulum. *Mol Syndromol* **1**: 99-112.

Ekström AB, Hakenäs-Plate L, Samuelsson L, Tulinius M, Wentz E. 2008. Autism spectrum conditions in myotonic dystrophy type 1: a study on 57 individuals with congenital and childhood forms. *Am J Med Genet B Neuropsychiatr Genet* **147B**: 918-926.

Ellaway CJ, Holme E, Standing S, Preece MA, Green A, Ploechl E, Ugarte M, Trefz, FK, Leonard JV. 2001. Outcome of tyrosinaemia type III. *J Inherit Metab Dis* **24**: 824-832.

Epi4K Consortium; Epilepsy Phenome/Genome Project, Allen AS, Berkovic SF, Cossette P, Delanty N, et al. 2013. De novo mutations in epileptic encephalopathies. *Nature* **501**: 217-221.

Evans E, Einfeld S, Mowat D, Taffe J, Tonge B, Wilson M. 2012. The behavioral phenotype of Mowat-Wilson syndrome. *Am J Med Genet A* **158A**: 358-366.

Farrant RD, Walker V, Mills GA, Mellor JM, Langley GJ. 2001. Pyridoxal phosphate de-activation by pyrroline-5-carboxylic acid. Increased risk of vitamin B6 deficiency and seizures in hyperprolinemia type II. *J Biol Chem* **276**: 15107-15116.

Feinstein M, Flusser H, Lerman-Sagie T, Ben-Zeev B, Lev D, Agamy O, et al. 2014. VPS53 mutations cause progressive cerebello-cerebral atrophy type 2 (PCCA2). *J Med Genet* **51**: 303-308.

Fischer B, Callewaert B, Schröter P, Coucke PJ, Schlack C, Ott CE, et al. 2014. Severe congenital cutis laxa with cardiovascular manifestations due to homozygous deletions in ALDH18A1. *Mol Genet Metab* **112**: 310-316.

FitzPatrick DR, Hill A, Tolmie JL, Thorburn DR, Christodoulou J. 1999. The molecular basis of malonyl-CoA decarboxylase deficiency.

FitzPatrick DR, Keeling JW, Evans MJ, Kan AE, Bell JE, Porteous ME, et al. 1998. Clinical phenotype of desmosterolosis. *Am J Med Genet* **75**: 145-152.

Fleisher TA. 2014. Autosomal recessive phosphoglucomutase 3 (PGM3) mutations link glycosylation defects to atopy, immune deficiency, autoimmunity, and neurocognitive impairment. *Pediatrics* **134**: S181-S182.

Froyen G, Belet S, Martinez F, Santos-Rebouças CB, Declercq M, Verbeeck J, Donckers L, Berland S, Mayo S, et al. 2012. Copy-number gains of HUWE1 due to replication- and recombination-based rearrangements. *Am J Hum Genet* **91**: 252-264.

Froyen G, Corbett M, Vandewalle J, Jarvela I, Lawrence O, Meldrum C, Bauters M, Govaerts K, Vandeleur L, Van Esch H, et al. 2008. Submicroscopic duplications of the hydroxysteroid dehydrogenase HSD17B10 and the E3 ubiquitin ligase HUWE1 are associated with mental retardation. *Am J Hum Genet* **82**: 432-443.

Gai X, Ghezzi D, Johnson MA, Biagosch CA, Shamseldin HE, Haack TB, et al. 2013. Mutations in FBXL4, encoding a mitochondrial protein, cause early-onset mitochondrial encephalomyopathy. *Am J Hum Genet* **93**: 482-495.

Garcia-Cruz D, Figuera LE, Feria-Velazco A, Sánchez-Corona J, García-Cruz M. O, Ramírez-Duenãs RM, Hernandez-Córdova A, Ruiz MX, Bitar-Alatorre WE, Ramírez-Duenãs ML, et al. 1993. The Myrhe syndrome: report of two cases. *Clin Genet* **44**: 203-207.

García-Silva MT, Matthijs G, Schollen E, Cabrera JC, Sanchez del Pozo J, Martí Herreros M, et al. 2004. Congenital disorder of glycosylation (CDG) type Ie. A new patient. *J Inherit Metab Dis* **27**: 591-600.

García-Villoria J, Navarro-Sastre A, Fons C, Pérez-Cerdá C, Baldellou A, Fuentes-Castelló MA, et al. 2009. Study of patients and carriers with 2-methyl-3-hydroxybutyryl-CoA dehydrogenase (MHBD) deficiency: difficulties in the diagnosis. *Clin Biochem* **42**: 27-33.

Gedeon AK, Nelson J, Gécz J, Mulley JC. 2003. X-linked mild non-syndromic mental retardation with neuropsychiatric problems and the missense mutation A365E in PAK3. *Am J Med Genet A* **120A**: 509-517.

Geraughty MT, Vaughn D, Nicholson AJ, Lin WW, Jimenez-Sanchez G, Obie C, et al. 1998. Mutations in the Delta1-pyrroline 5-carboxylate dehydrogenase gene cause type II hyperprolinemia. *Hum Mol Genet* **7**: 1411-1415.

Germanaud D, Rossi M, Bussy G, Gérard D, Hertz-Pannier L, Blanchet P, Dollfus H, Giuliano F, Bennouna-Greene V, Sarda P, et al. 2011. The Renpenning syndrome spectrum: new clinical insights supported by 13 new PQBP1-mutated males. *Clin Genet* **79**: 225-235.

Giannandrea M, Bianchi V, Mignogna ML, Sirri A, Carrabino S, D’Elia E, et al. 2010. Mutations in the small GTPase gene RAB39B are responsible for X-linked mental retardation associated with autism, epilepsy, and macrocephaly. *Am J Hum Genet* **86**: 185-195.

Gibbons RJ, Higgs DR. 2000. Molecular-clinical spectrum of the ATR-X syndrome. *Am J Med Genet* **97**: 204-212.

Gika AD, Hulse AJ, Josifova D, Baird G, Hedderly TJ. 2010. Autism stereotypies in Allan-Herndon-Dudley syndrome. *Movement Disord* **25**: S527.

Gillis D, Krishnamohan A, Yaacov B, Shaag A, Jackman JE, Elpeleg O. 2014. TRMT10A dysfunction is associated with abnormalities in glucose homeostasis, short stature and microcephaly. *J Med Genet* **51**: 581-586.

Goldberg MF, Cotlier E, Fichenscher LG, Kenyon K, Enat R, Borowsky SA. 1971. Macular cherry-red spot, corneal clouding, and beta-galactosidase deficiency. Clinical, biochemical, and electron microscopic study of a new autosomal recessive storage disease. *Arch Intern Med* **128**: 387-398.

Goodwin F, Muntoni F, Dubowitz V. 1997. Epilepsy in Duchenne and Becker muscular dystrophies. *Eur J Paediatr Neurol* **1**: 155-119.

Graziadio C, Rosa RF, Rosa RC, Zen PR, Flores JA, Paskulin GA. 2011. Short-term follow-up of a Brazilian patient with Cantú syndrome. *Am. J. Med. Genet. A* **155A**: 1184-1188.

Gribaa M, Salih M, Anheim M, Lagier-Tourenne C, H-mida D, Drouot N, et al. 2007. A new form of childhood onset, autosomal recessive spinocerebellar ataxia and epilepsy is localized at 16q21-a23.

Grozeva D, Carss K, Spasic-Boskovic O, Parker MJ, Archer H, Firth HV, Park SM, Canham N, Holder SE, Wilson M, et al. 2014. De novo loss-of-function mutations in SETD5, encoding a methyltransferase in a 3p25 microdeletion syndrome critical region, cause intellectual disability. *Am J Hum Genet* **94**: 618-624.

Grünewald S, Imbach T, Huijben K, Rubio-Gozalbo ME, Verrips A, de Klerk JB, et al. 2000. Clinical and biochemical characteristics of congenital disorder of glycosylation type Ic, the first recognized endoplasmic reticulum defect in N-glycan synthesis. *Ann Neurol* **47**: 776-781.

Guerguelcheva V, Azmanov DN, Angelicheva D, Smith KR, Chamova T, Florez L, et al. 2012. Autosomal-recessive congenital cerebellar ataxia is caused by mutations in metabotropic glutamate receptor 1. *Am J Hum Genet* **91**: 553-564.

Gulati S, Baker P, Li YN, Fowler B, Kruger W, Brody LC, et al. 1996. Defects in human methionine synthase in cbIG patients. *Hum Mol Genet* **5**: 1859-1865.

Hackett A, Tarpey PS, Licata A, Cox J, Whibley A, Boyle J, Rogers C, Grigg J, Partington M, Stevenson RE, et al. 2010. CASK mutations are frequent in males and cause X-linked nystagmus and variable XLMR phenotypes. *Eur J Hum Genet* **18**: 544-552.

Hagberg B, Aicardi J, Dias K, Ramos O. 1983. A progressive syndrome of autism, dementia, ataxia, and loss of purposeful hand use in girls: Rett’s syndrome: report of 35 cases. *Ann Neurol* **14**: 471-479.

Halgren C, Kjaergaard S, Bak M, Hansen C, El-Schich Z, Anderson CM, et al. 2012. Corpus callosum abnormalities, intellectual disability, speech impairment, and autism in patients with haploinsufficiency of ARID1B. *Clin Genet* **82**: 248-255.

Haliloglu G, Gross C, Senbil N, Talim B, Hehr U, Uyanik G, Winkler J, Topaloglu H. 2004. Clinical spectrum of muscle-eye-brain disease: from the typical presentation to severe autistic features. *Acta Myol* **23**: 137-139.

Hamdan FF, Daoud H, Piton A, Gauthier J, Dobrzeniecka S, Krebs MO, et al. 2011. De novo SYNGAP1 mutations in nonsyndromic intellectual disability and autism. *Biol Psychiatry* **69**: 898-901.

Hamdan FF, Saitsu H, Nishiyama K, Gauthier J, Dobrzeniecka S, Spiegelman D, et al. 2012. Identification of a novel in-frame de novo mutation in SPTAN1 in intellectual disability and pontocerebellar atrophy. *Eur J Hum Genet* **20**: 596-800.

Harbord MG, Baraitser M, Wilson J. 1989. Microcephaly, mental retardation, cataracts, and hypogonadism in sibs: Martsolf’s syndrome. *J Med Genet* **26**: 397-400.

Hardies K, May P, Djémié T, Tarta-Arsene O, Deconinck T, Craiu D; AR working group of the EuroEPINOMICS RES Consortium, Helbig I, Suls A, Balling R, Weckhuysen S, et al. 2014. Recessive loss-of-function mutations in AP4S1 cause mild fever-sensitive seizures, developmental delay and spastic paraplegia through loss of AP-4 complex assembly. *Hum Mol Genet* **24**: 2218-2227.

Harlalka GV, Baple EL, Cross H, Kühnle S, Cubillos-Rojas M, Matentzoglu K, Patton MA, Wagner K, Coblentz R, et al. 2013. Mutation of HERC2 causes developmental delay with Angelman-like features. *J Med Genet* **50**: 65-73.

Harvey K, Duguid IC, Alldred MJ, Beatty SE, Ward H, Keep NH, et al. 2004. The GDP-GTP exchange factor collybistin: an essential determinant of neuronal gephyrin clustering. *J Neurosci* **24**: 5816-5826.

Hehr U, Gross C, Diebold U, Wahl D, Beudt U, Heidemann P, Hehr A, Mueller D. 2004. Wide phenotypic variability in families with holoprosencephaly and a sonic hedgehog mutation. *Eur J Pediatr* **163**: 347-352.

Hehr U, Uyanik G, Gross C, Walter MC, Bohring A, Cohen M, Oehl-Jaschkowitz B, Bird LM, Shamdeen GM, Bogdahn U, et al. 2007. Novel POMGnT1 mutations define broader phenotypic spectrum of muscle-eye-brain disease. *Neurogenetics* **8**: 279-288.

Heinzen EL, Swoboda KJ, Hitomi Y, Gurrieri F, Nicole S, de Vries B, et al. 2012. De novo mutations in ATP1A3 cause alternating hemiplegia of childhood. *Nat Genet* **44**: 1030-1034.

Heller ER, Khan SG, Kuschal C, Tamura D, DiGiovanna JJ, Kraemer KH. 2015. Mutations in the TTDN1 gene are associated with a distinct trichothiodystrophy phenotype. *J Invest Dermatol* **135**: 734-741.

Helsmoortel C, Vulto-van Silfhout AT, Coe BP, Vandeweyer G, Rooms L, van den Ende J, et al. 2014. A SWI/SNF-related autism syndrome caused by de novo mutations in ADNP. *Nat Genet* **46**: 380-384.

Hengst M, Tücke J, Zerres K, Blaum M, Häusler M. 2010. Megalencephaly, mega corpus callosum, and complete lack of motor development: delineation of a rare syndrome. *Am J Med Genet* **152A**: 2360-2364.

Henneke M, Preuss N, Engelbrecht V, Aksu F, Bertini E, Bibat G, et al. 2005. Cystic leukoencephalopathy without megalencephaly: a distinct disease entity in 15 children. *Neurology* **64**: 1411-1416.

Héron B, Mikaeloff Y, Froissart R, Caridade G, Maire I, Caillaud C, et al. 2011. Incidence and natural history of mucopolysaccharidosis type III in France and comparison with United Kingdom and Greece. *Am J Med Genet A* **155A**: 58-68.

Heron SE, Smith KR, Bahlo M, Nobili L, Kahana E, Licchetta L, et al. 2012. Missense mutations in the sodium-gated potassium channel gene KCNT1 cause severe autosomal dominant nocturnal frontal lobe epilepsy. *Nat Genet* **44**: 1188-1190.

Heylen E, Scherer G, Vincent MF, Marie S, Fischer J, Nassogne MC. 2012. Tyrosinemia type III detected via neonatal screening: management and outcome. *Mol Genet Metab* **107**: 605-607.

Hing AV, Syed N, Cunningham ML. 2004. Familial acromelic frontonasal dysostosis: autosomal dominant inheritance with reduced penetrance. *Am J Med Genet* **128A**: 374-382.

Hoefnagel D, Pomeroy J, Wurster D, Saxon A. 1971. Congenital athetosis, mental deficiency, dwarfism and laxity of skin and ligaments. *Helv Paediatr Acta* **26**: 397-402.

Hoffman WH, Lee JR, Kovacs K, Chen H, Yaghmai F. 2007. Johanson-Blizzard syndrome: autopsy findings with special emphasis on hypopituitarism and review of the literature. *Pediatr Dev Pathol* **10**: 55-60.

Hoischen A, van Bon BW, Rodríguez-Santiago B, Gilissen C, Vissers LE, de Vries P, et al. 2011. De novo nonsense mutations in ASXL1 cause Bohring-Opitz syndrome. *Nat Genet* **26**: 729-731.

Holmberg V, Lauronen L, Autti T, Santavuori P, Savukoski M, Uvebrant P, et al. 2000. Phenotype-genotype correlation in eight patients with Finnish variant late infantile NCL (CLN5). *Neurology* **55**: 579-581.

Homan CC, Kumar R, Nguyen LS, Haan E, Raymond FL, Abidi F, Raynaud M, Schwartz CE, Wood SA, Gecz J, et al. 2014. Mutations in USP9X are associated with X-linked disability and disrupt neuronal cell migration and growth. *Am J Hum Genet* **94**: 470-478.

Houten SM, Te Brinke H, Denis S, Ruiter JP, Knegt AC, de Klerk JB, et al. 2013. Genetic basis of hyperlysinemia. *Orphanet J Rare Dis* **8**: 57.

Howard MF, Murakami Y, Pagnamenta AT, Daumer-Haas C, Fischer B, Hecht J, et al. 2014. Mutations in PGAP3 impair GPI-anchor maturation, causing a subtype of hyperphosphatasia with mental retardation. *Am J Hum Genet* **94**: 278-287.

Howlin P, Karpf J, Turk J. 2005. Behavioural characteristics and autistic features in individuals with Cohen syndrome. *Eur. Child. Adolesc. Psychiatry* **14**: 57-64.

Hoyer J, Ekici AB, Endele S, Popp B, Zweier C, Wiesener A, Wohlleber E, Dufke, A, Rossier E, Petsch C, et al. 2012. Haploinsufficiency of ARID1B, a member of the SWI/SNF-a chromatin-remodeling complex, is a frequent cause of intellectual disability. *Am J Hum Genet* **90**: 565-572.

Huppke P, Wegener E, Böher-Rabel H, Bolz HJ, Zoll B, Gärtner J, Bergmann C. 2014. Tectonic gene mutations in patients with Joubert syndrome. *Eur J Hum Genet* **23**: 616-620*.*

Hyohyeon C, Lee CG. 2015. A 13-year-old boy with a 7q36.1-q36.3 deletion with additional findings. *Am J Med Genet A* **167A**: 198-203.

Imbach T, Schenk B, Schollen E, Burda P, Stutz A, Grunewald S, et al. 2000. Deficiency of dolichol-phosphate-mannose synthase-1 causes congenital disorder of glycosylation type Ie. *J Clin Invest* **105**: 233-239.

Iqbal Z, Vandeweyer G, van der Voet M, Waryah AM, Zahoor MY, Besseling JA, Roca LT, Vulto-van Silfhout AT, Nijhof B, Kramer JM, et al. 2013. Homozygous and heterozygous disruptions of ANK3: at the crossroads of neurodevelopmental and psychiatric disorders. *Hum Mol Genet* **22**: 1960-1970.

Igoillo-Esteve M, Genin A, Lambert N, Désir J, Pirson I, Abdulkarim B, et al. 2013. tRNA methyltransferase homolog gene TRMT10A mutation in young onset diabetes and primary microcephaly in humans. *PLoS Genet* **9**: e1003888.

Ivanov IS, Azmanov DN, Ivanova MB, Chamova T, Pacheva IH, Panova MV, et al. 2014. Founder p.Arg 446* mutations in the PDHX gene explains over half of cases with congenital lactic acidosis in Roma children. *Mol Genet Metab* **113**: 76-83.

Ito S, Nakayama T, Ide S, Ito Y, Oguni H, Goto Y, Osawa M. 2008. Aromatic L-amino acid decarboxylase deficiency associated with epilepsy mimicking non-epileptic involuntary movements. *Dev Med Child Neurol* **50**: 876-878.

Jackson SR, Guner YS, Woo R, Randolph LM, Ford H, Shin CE. 2009. L1CAM mutation in association with X-linked hydrocephalus and Hirschsprung’s disease. *Pediatr Surg Int* **25**: 823-825.

Jaeken J, Detheux M, Van Maldergem L, Foulon M, Carchon H, Van Schaftingen E. 1996. 3-Phosphoglycerate dehydrogenase deficiency: an inborn error of serine biosynthesis. *Arch Dis Child* **74**: 542-545.

Jaeken J, Wadman SK, Duran M, van Sprang FJ, Beemer FA, Holl RA, et al. 1988. Adenylosuccinase deficiency: an inborn error of purine nucleotide synthesis. *Eur J Pediatr* **148**: 126-131.

Jansen J, Friesema EC, Kester MH, Schwartz CE, Visser TJ. 2008. Genotype-phenotype relationship in patients with mutations in thyroid hormone transporter MCT8. *Endocrinology* **149**: 2184-2190.

Johnson JP, Carey JC, Glassy FJ, Paglieroni T, Lipson MH. 1983. Marshall-Smith syndrome: two case reports and a review of pulmonary manifestations. *Pediatrics* **71**: 219-223.

Johnston JJ, Gropman AL, Sapp JC, Teer JK, Martin JM, Liu CF, et al. 2012. The phenotype of a germline mutation in PIGA: the gene somatically mutated in paroxysmal nocturnal hemoglobinuria. *Am J Hum Genet* **90**: 295-300.

Kanavakis E, Xaidara A, Papathanasiou-Klontza D, Papadimitriou A, Velentza S, Youroukos S. 2003. Alternating hemiplegia of childhood: a syndrome inherited with an autosomal dominant trait. *Dev Med Child Neurol* **45**: 833-836.

Kantojärvi K, Kotala I, Rehnström K, Ylisaukko-Oja T, Vanhala R, von Wendt TN, von Wendt L, Järvelä I. 2011. Fine mapping of Xq11.1-q21.33 and mutation screening of RPS6KA6, ZNF711, ACSL4, DLG3, and IL1RAPL2 for autism spectrum disorders (ASD). *Autism Res* **4**: 228-233.

Karaca E, Weitzer S, Pehlivan D, Shiraishi H, Gogakos T, Hanada T, et al. 2014. Human CLP1 mutations alter tRNA biogenesis, affecting both peripheral and central nervous system function. *Cell* **157**: 636-650.

Kato M, Saitsu H, Murakami Y, Kikuchi K, Watanabe S, Iai M, et al. 2014. PIGA mutations cause early-onset epileptic encephalopathies and distinctive features. *Neurology* **82**: 1587-1596.

Kavaslar GN, Onenüt S, Derman O, Kaya A, Tolun A. 2000. The novel genetic disorder microhydranencephaly maps to chromosome 16p13.3-12.1. *Am J* *Hum Genet* **66**: 1705-1709.

Khalifa O, Imtiaz F, Al-Sakati N, Al-Manea K, Verloes A, Al-Owain M. 2011. Dyggve-Melchior-Clausen syndrome: novel splice mutation with atlanto-axial subluxation. *Eur J Pediatr* **170**: 121-126.

Kheradmand Kia S, Verbeek E, Engelen E, Schot R, Poor RA, de Coo IF, et al. 2012. RTTN mutations links primary cilia function to organization of the human cerebral cortex. *Am J Hum Genet* **91**: 533-540.

Kiani R, Gangadharan SK, Miller H. 2007. Case report: association of Waardenburg syndrome with intellectual disability, autism spectrum disorder and unprovoked aggressive outbursts: a new behavioural phenotype? *Brit J Dev Disab* **53**: 53-62.

Kim SA, Kim JH, Park M, Cho IH, Yoo HJ. 2007. Family-based association study between GRIK2 polymorphisms and autism spectrum disorders in the Korean trios. *Neurosci Res* **58**: 332-335.

Kim S, Westphal V, Srikrishna G, Mehta DP, Peterson S, Filiano J, et al. 2000. Dolichol phosphate mannose synthase (DPM1) mutations define congenital disorder of glycosylation Ie (CDG-Ie). *J Clin Invest* **105**: 191-198.

Kimonis VE, Steller J, Sahai I, Grange DK, Shoemaker J, Zelaya BM, Mandell R, Shih K, Shih V. 2012. Mild fumarase deficiency and a trial of low protein diet. *Mol Genet Metab* **107**: 241-242.

Kivuva EC, Parker MJ, Cohen MC, Wagner BE, Sobey G. 2008. De Barsy syndrome: a review of the phenotype. *Clin Dysmorphol* **17**: 99-107.

Kleefstra T, Yntema HG, Oudakker AR, Banning MJ, Kalscheuer VM, Chelly J, Moraine C, Ropers HH, Fryns JP, Janssen IM, et al. 2004. Zinc finger 81 (ZNF81) mutations associated with X-linked mental retardation. *J Med Genet* **41**: 394-399.

Kluk MJ, An Y, James P, Coulter D, Harris D, Wu BL, Shen Y. 2011. Avoiding pitfalls in molecular genetic testing: case studies of high-resolution array comparative genomic hybridization testing in the definitive diagnosis of Mowat-Wilson syndrome. *J Mol Diagn* **13**: 363-367.

Koolen DA, Sharp AJ, Hurst JA, Firth HV, Knight SJ, Goldenberg A, et al. 2008. Clinical and molecular delineation of the 17q21.31 microdeletion syndrome. *J Med Genet* **45**: 710-720.

Kortüm F, Das S, Flindt M, Morris-Rosendahl DJ, Stefanova I, Goldstein A, Horn D, Klopocki E, Kluger G, Martin P, et al. 2011. The core FOXG1 syndrome phenotype consists of postnatal microcephaly, severe mental retardation, absent language, dyskinesia, and corpus callosum hypogenesis. *J Med Genet* 48: 396-406.

Kousi M, Anttila V, Schulz A, Calafato S, Jakkula E, Riesch E, et al. 2012. Novel mutations consolidate KCTD7 as a progressive myoclonus epilepsy gene. *J Med Genet* **49**: 391-399.

Krawitz PM, Murakami Y, Reiss A, Hietala M, Krüger U, Zhu N, et al. 2013. PGAP2 mutations, affecting the GPI-anchoring-synthesis pathway, cause hyperphosphatasia with mental retardation syndrome. *Am J Hum Genet* **92**: 584-589.

Kvittingen EA, Spangen S, Lindemans J, Fowler B. 1997. Methionine synthase deficiency without megaloblastic anaemia. *Eur J Pediatr* **156**: 925-930.

Labrune P, Zittoun J, Duvaltier I, Trioche P, Marquet J, Niaudet P, et al. 1999. Haemolytic uraemic syndrome and pulmonary hypertension in a patient with methionine synthase deficiency. *Eur J Pediatr* **158**: 734-739.

Lacbawan F, Solomon BD, Roessler E, El-Jaick K, Domené S, Vélez JI, Zhou N, Hadley D, Balog JZ, Long R, et al. 2009. Clinical spectrum of SIX3-associated mutations in holoprosencephaly: correlation between genotype, phenotype and function. *J Med Genet* **46**: 389-398.

Laje G, Morse R, Richter W, Ball J, Pao M, Smith AC. 2010. Autism spectrum features in Smith-Magenis syndrome. *Am J Med Genet C Semin Med Genet* **154C**: 456-462.

Lam CW, Law CY, Leung KF, Lai CK, Chen SP. 2014. NMR-based urinalysis for rapid diagnosis of β-ureidopropionase deficiency in a patient with Dravet syndrome. *Clin Chim Acta* **S0009-8981**: 00461-6.

Laumonnier F, Bonnet-Brilhault F, Gomot M, Blanc R, David A, Moizard MP, Raynaud M, Ronce,N, Lemonnier E, Calvas P, et al. 2004. X-linked mental retardation and autism are associated with a mutation in the NLGN4 gene, a member of the neuroligin family. *Am J Hum Genet* **74**: 552-557.

Laumonnier F, Holbert S, Ronce N, Faravelli F., Lenzner S, Schwartz CE, Lespinasse J, Van Esch H, Lacombe D, Goizet C, et al. 2005. Mutations in PHF8 are associated with X linked mental retardation and cleft lip/cleft palate. *J Med Genet* **42**: 780-786.

Leclerc D, Campeau E, Goyette P, Adjalla CE, Christensen B, Ross M, et al. 1996. Human methionine synthase: cDNA cloning and identification of mutations in patients of the cbIG complementation group of folate/cobalamin disorders. *Hum Mol Genet* **5**: 1867-1874.

Le Fevre AK, Taylor S, Malek NH, Horn D, Carr CW, Abdul-Rahman OA, et al. 2013. FOXP1 mutations cause intellectual disability and a recognizable phenotype. *Am J Med Genet A* **161A**: 3166-3175.

Leger PL, Souville I, Boddaert N, Elie C, Pinard JM, Plouin P, et al. 2008. The location of DCX mutations predicts malformation severity in X-linked lissencephaly. *Neurogenetics* **9**:277-285.

Lesca G, Moizard MP, Bussy G, Boggio D, Hu H, Haas SA, Ropers HH, Kalscheuer VM, Des Portes V, Labalme A, et al. 2013. Clinical and neurocognitive characterization of a family with a novel MED12 gene frameshift mutation. *Am J Med Genet A* **161**: 3063-3071.

Li BM, Liu XR, Yi YH, Deng YH, Su T, Zou X, Liao WP. 2011. Autism in Dravet syndrome: prevalence, features, and relationship to the clinical characteristics of epilepsy and mental retardation. *Epilepsy Behav* **21**: 291-295.

Liegel RP, Handley MT, Ronchetti A, Brown S, Langemeyer L, Linford A, et al. 2013. Loss-of-function mutations in the TBC1D20 cause cataracts and male infertility in blind sterile mice and Warburg micro syndrome in humans. *Am J Hum Genet* **93**: 1001-1014.

Lines MA, Huang L, Schwartzentruber J, Douglas SL, Lynch DC, Beaulieu C, et al. 2012. Haploinsufficiency of a spliceosomal GTPase encoded by EFTUD2 causes mandibulofacial dysostosis with microcephaly. *Am J Hum Genet* **90**: 369-377.

Lo-Castro A, Giana G, Fichera M, Castiglia L, Grillo L, Musumeci SA, Galasso C, Curatolo P. 2009. Deletion 2p25.2: a cryptic chromosome abnormality in a patient with autism and mental retardation detected using aCGH. *Eur J Med Genet* **52**: 67-70.

López-Hernández T, Ridder MC, Montolio M, Capdevila-Nortes X, Polder E, Sirisi S, Duarri A, Schulte U, Fakler B, Nunes V, et al. (2011). Mutant GlialCAM causes megalencephalic leukoencephalopathy with subcortical cysts, benign familial macrocephaly, and macrocephaly with retardation and autism. *Am J Hum Genet* **88**: 422-432.

Makrythanasis P, Nelis M, Santoni FA, Guipponi M, Vannier A, Béna F, et al. 2014. Diagnostic exome sequencing to elucidate the genetic basis of likely recessive disorders in consanguineous families. *Hum Mutat* **35**: 1203-1210.

Mallaret M, Synofrik M, Lee J, Sagum CA, Manhajnah M, Sharkia R, et al. 2014. The tumour suppressor gene WWOX is mutated in autosomal recessive cerebellar ataxia with epilepsy and mental retardation. *Brain* **137**: 411-419.

Malm G, Månsson JE. 2010. Mucopolysaccharidosis type III (Sanfilippo disease) in Sweden: Clinical presentation of 22 children diagnosed during a 30-year period. *Acta Paediatr* **99**: 1253-1257.

Manouvrier-Hanu S, Amiel J, Jacquot S, Merienne K, Moerman A, Coëslier A, Labarriere F, Vallée L, Croquette MF, et al. 1999. Unreported RSK2 missense mutation in two male sibs with an unusually mild form of Coffin-Lowry syndrome. *J Med Genet* **36**: 775-778.

Manzini MC, Xiong L, Shaheen R, Tambunan DE, Di Costanzo S, Mitisalis V, Tischfield DJ, Cinquino A, Ghaziuddin M, Christian M, et al. 2014. CC2D1A regulated human intellectual and social function as well as NF-κB signaling homeostasis. *Cell Rep* **8**: 647-655.

Marco EJ, Abidi FE, Bristow J, Dean WB, Cotter P, Jeremy RJ, et al. 2008. ARHGEF9 disruption in a female patient is associated with X linked mental retardation and sensory hyperarousal. *J Med Genet* **45**: 100-105.

Masurel-Paulet A, Kalscheuer VM, Lebrun N, Hu H, Levy F, Thauvin-Robinet C, Darmency-Stamboul V, El Chehadeh S, Thevenon J, Chancenotte S, et al. 2014. Expanding the clinical phenotype of patients with a ZDHHC9 mutation. *Am J Med Genet* **164A**: 789-795.

Matsumoto A, Kuwajima M, Miyake K, Kojima K, Nakashima N, Jimbo EF, Kubota T, Momoi MY, Yamagata T. 2013. An Xp22.12 microduplication including RPS6KA3 identified in a family with variably affected intellectual and behavioral disabilities. *J Hum Genet* **58**: 755-757.

McLarren KW, Severson TM, du Souich C, Stockton DW, Kratz LE, Cunningham D, et al. 2010. Hypomorphic temperature-sensitive alleles of NSDHL cause CK syndrome. *Am J Hum Genet* **87**: 905-914.

Mercimek-Mahmutoglu S, Ndika J, Kanhai W, de Villemeur TB, Cheillan D, Christensen E, et al. 2014. Thirteen new patients with guanidioacetate methyltransferase deficiency and functional characterization of nineteen novel missense variants in the GAMT gene. *Hum Mutat* **35**: 462-469.

Ming JE, Kaupas ME, Roessler E, Brunner HG, Golabi M, Tekin M, Stratton RF, Sujansky E, Bale SJ, Muenke M. 2002. Mutations in PATCHED-1, the receptor for SONIC HEDGEHOG, are associated with holoprosencephaly. *Hum Genet* **110**: 297-301.

Mirzaa G, Dodge NN, Glass I, Day C, Gripp K, Nicholson L, et al. 2004. Megalencephaly and perisylvian polymicrogyria with postaxial polydactyly and hydrocephalus: a rare brain malformation syndrome associated with mental retardation and seizures. *Neuropediatrics* **35**: 353-359.

Mirzaa GM, Rivière JB, Dobyns WB. 2013. Megalencephaly syndromes and activating mutations in the PI3K-AKT pathway: MPPH and MCAP. *Am J Med Genet C Semin Med Genet* **163C**: 122-130.

Misceo D, Fannemel M, Barøy T, Roberto R, Tvedt B, Jaeger T, Bryn V, Strømme P, Frengen E. 2009. SCA27 caused by a chromosome translocation: further delineation of the phenotype. *Neurogenetics* **10**: 371-374.

Moin M, Aghamohammadi A, Kouhi A, Tavassoli S, Rezaei N, Ghaffari SR, Gharagozlou M, Movahedi M, Purpak Z, Mirsaeid Ghazi B, et al. 2007. Ataxia-telangiecstasia in Iran: clinical and laboratory features of 104 patients. *Pediatr Neurol* **37**: 21-28.

Mole SE, Williams RE, Goebel HH. 2005. Correlations between genotype, ultrastructural morphology and clinical phenotype in the neuronal ceroid lipofuscinoses. *Neurogenetics* **6**: 107-126.

Moore SJ, Green JS, Fan Y, Bhogal AK, Dicks E, Fernandez BA, Stefanelli M, Murphy C, Cramer BC, Dean JC, et al. 2005. Clinical and genetic epidemiology of Bardet-Biedl syndrome in Newfoundland: a 22-year prospective, population-based, cohort study. *Am J Med Genet* **132**: 352-360.

Morava E, Lefeber DJ, Urban Z, de Meirleir L, Meinecke P, Gillessen-Kaesbach G, et al. 2008. Defining the phenotype in an autosomal recessive cutis laxa syndrome with a combined congenital defect of glycosylation. *Eur J Hum Genet* **16**: 28-35.

Morey-Canellas J, Sivagamasundari U, Barton H. 2003. A case of autism in a child with Apert’s syndrome. *Eur Child Adolesc Psychiatry* **12**: 100-102.

Mory A, Dagan E, Shahor I, Mandel H, Illi B, Zolotushko J, Kurolap A, Chechik E, Valente EM, Amselem S, et al. 2014. Kohlschutter-Tonz syndrome: clinical genetic insights gained from 16 cases deriving from a close-knit village in Northern Israel. *Pediatr Neurol* **50**: 421-426.

Moss, JF, Oliver C, Berg K, Kaur G, Jephcott L, Cornish K. 2008. Prevalence of autism spectrum phenomenology in Cornelia de Lange and Cru du Chat syndromes. *Am J Ment Retard* **113**: 278-291.

Musumeci SA, Ferri R, Scuderi C, Bosco P, Elia M. 2001. Seizures and epileptiform EG abnormalities in FRAXE syndrome. *Clin Meurophysiol* **112**: 1954-1955.

Nakamura M, Yamagata T, Momoi MY, Yamazaki T. 1998. Drop episodes in Coffin-Lowry syndrome: exaggerated startle responses treated with clonazepam. *Pediatr Neurol* **19**: 148-150.

Nankovic S, Kovac AB, Hajnsek S, Sulentic V, Petelin Z. 2012. Ritscher-Schinzel syndrome-3C (Cranio-Cerebello-Cardiac) syndrome: case report. (T. Grivas, ed.). In: Recent Advances in Scoliosis. INTECH Open Access Publisher, pp. 39-46.

Nascimento RM, Otto PA, de Brouwer AP, Vianna-Morgante AM. 2006. UBE2A, which incodes a ubiquitin-conjugating enzyme, is mutated in a novel X-linked mental retardation syndrome. *Am J Hum Genet* **79**: 549-555.

Nava C, Dalle C, Rastetter A, Striano P, de Kovel CG, Nabbout R, Cancès C, Ville D, Brilstra EH, Gobbi G, et al. 2014. De novo mutations in HCN1 cause early infantile epileptic encephalopathy. *Nat Genet* **46**: 640-645.

Nava C, Hanna N, Michot C, Pereira S, Pouvreau N, Niihori T, et al. 2007. Cardio-facio-cutaneous and Noonan syndromes due to mutations in the RAS/MAPK signaling pathway: genotype-phenotype relationships and overlap with Costello syndrome. *J Med Genet* **44**: 763-771.

Nava C, Lamari F, Héron D, Mignot C, Rastetter A, Keren B, Cohen D, Faudet A, Bouteiller D, Gilleron M, et al. 2012. Analysis of the chromosome X exome in patients with autism spectrum disorders identified novel candidate genes, including TMLHE. *Transl Psychiatry* **2**: e179.

Ndika JD, Johnston K, Barkovich JA, Wirt MD, O’Neill P, Betsalel OT, Jakobs C, Salomons GS. 2012. Developmental progress and creatine restoration upon long-term creatin supplementation of a patient with arginine:glycine amidinotransferase deficiency. *Mol Genet Metab* **106**: 48-54.

Neilson DE, Adams MD, Orr CM, Schelling DK, Eiben RM, Kerr DS, et al. 2009. Infection-triggered familiar or recurrent cases of acute necrotizing encephalopathy caused by mutations in a component of the nuclear pore, RANBP2. *Am J Hum Genet* **84**: 44-51.

Nguyen LS, Jolly L, Shoubridge C, Chan WK, Huang L, Laumonnier F, Raynaud M, Hackett A, Field M, Rodriguez J, et al. 2012. Transcriptome profiling of UPF3B/NMD-deficient lymphoblastoid cells from patients with various forms of intellectual disability. *Mol Psychiatry* **17**: 1103-1115.

Nomura T, Koyama N, Yokoyama M, Awaya A, Yokochi K. 2009. DOOR syndrome concomitant with non-convulsive status epilepticus and hyperintense cerebellar cortex on T2-weighted imaging. *Brain Dev* **31**: 75-78.

Nonoda Y, Saito Y, Nagai S, Sasaki M, Iwasaki T, Matsumoto N, et al. 2013. Progressive diffuse brain atrophy in West syndrome with marked hypomyelination due to SPTAN1 gene mutation. *Brain Dev* **35**: 280-283.

Novarino G, El-Fishawy P, Kayserili H, Meguid NA, Scott EM, Schroth J, et al. 2012. Mutations in BCKD-kinase lead to a potentially treatable form of autism with epilepsy. *Science* **338**: 394-397.

Numis AL, Major P, Montenegro MA, Muzykewicz DA, Pulsifer MB, Thiele EA. 2011. Identification of risk factors for autism spectrum disorders in tuberous sclerosis complex. *Neurology* **76**: 981-987.

Ockeloen CW, Willemsen MH, de Munnik S, van Bon BW, de Leeuw N, Verrips A, et al. 2015. Further delineation of the KBG syndrome phenotype caused by ANKRD11 aberrations. *Eur J Hum Genet* **23**: 1176-1185.

O’Leary RE, Shih JC, Hyland K, Kramer N, Asher YJ, Graham JM Jr. 2012. De novo microdeletion of Xp11.3 exclusively encompassing the monoamine oxidase A and B genes in a male infant with episodic hypotonia: a genomics approach to personalized medicine. *Eur J Med Genet* **55**: 349-353.

Oliver C, Berg K, Moss J, Arron K, Burbidge C. 2011. Delineation of behavioral phenotypes in genetic syndromes: characteristics of autism spectrum disorder, affect and hyperactivity. *J Autism Dev Disord* **41**: 1019-1032.

Ozand PT, Gascon GG, Al Essa M, Joshi S, Al Jishi E, Bakheet S, et al. 1998. Bioin-responsive basal ganglia disease: a novel entity. *Brain* **121**: 1267-1279.

Ozgen HM, van Daalen E, Bolton PF, Maloney VK, Huang S, Cresswell L, van den Boogaard MJ, Eleveld MJ, van ‘t Slot R, Hochstenbach R, et al. 2009. Copy number changes of the microcephalin 1 gene (MCPH1) in patients with autism spectrum disorders. *Clin Genet* **76**: 348-356.

Oz-Levi D, Ben-Zeev B, Ruzzo EK, Hitomi Y, Gelman A, Pelak K, et al. 2012. Mutation in TECPR2 reveals a role for autophagy in hereditary spastic paraparesis. *Am J Hum Genet* **91**: 1065-1072.

Palmer EE, Leffler M, Rogers C, Shaw M, Carroll R, Earl J, et al. 2016. New insights into Brunner syndrome and potential for targeted therapy. *Clin Genet* **89**: 120-127.

Passemard S, Titomanlio L, Elmaleh M, Afenjar A, Alessandri JL, Andria G, et al. 2009. Expanding the clinical and neuroradiologic phenotype of primary microcephaly due to ASPM mutations. *Neurology* **73**: 962-969.

Pavlović M, Neubauer D, Al Tawari A, Heberle LC. 2014. The microcephaly-capillary malformation syndrome in two brothers with novel clinical features. *Pediatr Neurol* **51**: 560-565.

Pedersen PS, Christensen E, Brandt NJ. 1983. Prolidase deficiency. *Acta Paediatr Scand* **72**: 785-788.

Peippo M, Koivisto AM, Särkämö T, Sipponen M, von Koskull H, Ylisaukko-oja T, et al. 2007. PAK3 related mental disability: further characterization of the phenotype. *Am J Med Genet A* **143A:** 2406-2416.

Pescosolido MF, Schwede M, Johnson Harrison A, Schmidt M, Gamsiz ED, Chen WS, et al. 2014. Expansion of the clinical phenotype associated with mutations in activity-dependent neuroprotective protein. *J Med Genet* **51**: 587-589.

Phelan MC, Rogers RC, Saul RA, Stapleton GA, Sweet K, McDermid H, et al. 2001. 22q13 deletion syndrome. *Am J Med Genet* **101**: 91-99.

Philips AK, Sirén A, Avela K, Somer M, Peippo M, Ahvenainen M, et al. 2014. X-exome sequencing in Finnish families with intellectual disability—four novel mutations and two novel syndromic phenotypes. *Orphanet J Rare Dis* **9**: 49.

Pinto D, Delaby E, Merico D, Barbosa M, Merikangas A, Klei L, et al. 2014. Convergence of genes and cellular pathways dysregulated in autism spectrum disorders. *Am J Hum Genet* **94**: 677-694.

Piton A, Gauthier J, Hamdan FF, Lafrenière RG, Yang Y, Henrion E, Laurent S, Noreau A, Thibodeau P, Karemera L, et al. 2011. Systematic resequencing of X-chromosome synaptic genes in autism spectrum disorder and schizophrenia. *Mol Psychiatry* **16**: 867-880.

Piton A, Poquet H, Redin C, Masurel A, Lauer J, Muller J, et al. 2014. 20 ans après: a second mutation in MAOA identified by targeted high-throughput sequencing in a family with altered behavior and cognition. *Eur J Hum Genet* **22**: 776-783.

Poirier K, Keays DA, Francis F, Saillour Y, Bahi N, Manouvrier S, Fallet-Bianco C, Pasquier L, Toutain A, Tuy FP, et al. 2007. Large spectrum of lissencephaly and pachygyria phenotypes resulting from de novo missense mutations in tubulin alpha 1A (TUBA1A). *Hum Mutat* **28:** 1055-1064.

Poirier K, Lebrun N, Broix L, Tian G, Saillour Y, Boscheron C, et al. 2013. Mutations in the TUBG1, DYNC1H1, KIF5C and KIF2A cause malformations of cortical development and microcephaly. *Nat Genet* **45**: 639-647.

Poirier K, Saillour Y, Bahi-Buisson N, Jaglin XH, Fallet-Bianco C, Nabbout R, et al. 2010. Mutations in the neuronal beta-tubulin subunit TUBB3 result in malformation of cortical development and neuronal migration defects. *Hum Mol Genet* **19**: 4462-4473.

Pontz BF, Zepp F, Stöss H. 1986. Biochemical, morphological and immunological findings in a patient with a cutis laxa-associated inborn disorder (De Barsy syndrome). *Eur J Pediatr* **145**: 428-434.

Prasad AN, Breen JC, Ampola MG, Rosman NP. 1997. Argininemia: a treatable genetic cause of progressive spastic diplegia simulating cerebral palsy: case reports and literature review. *J Child Neurol* **12**: 301-309.

Prasad C, Rupar T, Prasad AN. 2011. Pyruvate dehydrogenase deficiency and epilepsy. *Brain Dev* **33**: 856-865.

Priolo M, Grosso E, Mammì C, Labate C, Naretto VG, Valcalebre C, Caridi P, Laganà. 2012. A peculiar mutation in the DNA-binding/dimerization domain of NFIX causes Sotos-like overgrowth syndrome: a new case. *Gene* **511**: 103-105.

Proud VK, Levine C, Carpenter NJ. 1992. New X-linked syndrome with seizures, acquired micrencephaly, and agenesis of the corpus callosum. *Am J Med Genet* **43**: 458-466.

Puffenberger EG, Jinks RN, Sougnez C, Cibulskis K, Willert RA, Achilly NP, et al. 2012a. Genetic mapping and exome sequencing identify variants associated with five novel diseases. *PLoS One* **7**: e28936.

Puffenberger EG, Jinks RN, Wang H, Xin B, Fiorentini C, Sherman EA, Degrazio D, Shaw C, Sougnez C, Cibulskis K., et al. 2012b. A homozygous missense mutation in HERC2 associated with global developmental delay and autism spectrum disorder. *Hum Mutat* **33**: 1639-1646.

Quinlivan R, Mitsuahashi S, Sewry C, Cirak S, Aoyama C, Moore D, Abbs S, Robb S, Newton T, Moss C, et al. 2013. Muscular dystrophy with large mitochondria associated with mutations in the CHKB gene in three British patients: Extending the clinical and pathological phenotype. *Neuromuscul Disord* **23**: 549-556.

Rabier D, Nuttin C, Poggi F, Padovani JP, Abdo K, Bardet J, et al. 1992. Familial joint hyperlaxity, skin hyperelasticity, cataract and mental retardation with hyperammoneia and low citrulline, ornithine and proline: a new disorder of collagen metabolism? Abstracts of Free Communications, 30^th^ Annual Symposium Leuven, Sept. 8-11. The Society for the Study of Inborn Errors of Metabolism (SSIEM): Leuven, 1992, p. 61.

Rakkolainen A, Ala-Mello S, Kristo P, Orpana A, Järvelä I. 2002. Four novel mutations in the OFD1 (Cxorf5) gene in Finnish patients with oral-facial-digital syndrome 1. *J Med Genet* **39**: 292-296.

Ramaekers V, Blau N. 2004. Cerebral folate deficiency. *Dev Med Child Neurol* **46**: 843-851.

Ramantani G, Maillard LG, Bast T, Husain RA, Niggemann P, Kohlhase J, Hertzberg C, Ungerath K, Innes MA, Walkenhorst H, et al. 2014. Epilepsy in Aicardi-Goutières syndrome. *Eur J Paediatr Neurol* **18**: 30-37.

Ramer, J. C., Frankel, C. A., and Ladda, R. L. (1993). Marden-Walker phenotype: spectrum of variability in three infants. *Am J Med Genet* **45**: 285-291.

Ramocki MB, Peters SU, Tavyev YJ, Zhang F, Carvalho CM, Shaaf CP, Richman R, Fang P, Glaze DG, Lupski JR, et al. 2009. Autism and other neuropsychiatric symptoms are prevalent in individuals with MeCP2 duplication syndrome. *Ann Neurol* **66**: 771-782.

Ramos JM, Davis GJ, Hunsaker JC 3^rd^, Balko MG. 2009. Sudden death in a child with Carpenter syndrome. Case report and literature review. *Forensic Sci Med Pathol* **5**: 313-317.

Reardon W, Hockey A, Silberstein P, Kendall B, Farag TI, Swash M, et al. 1994. Autosomal recessive congenital intrauterine infection-like syndrome of microcephaly, intracranial calcification, and CNS disease. *Am J Med Genet* **52**: 58-65.

Reinthaler EM, Lal D, Jurkowski W, Feucht M, Steinböck H, Gruber-Sedlmayr U, Ronen GM, Geldner J, Haberlandt E, Neophytou B, et al. 2014. Analysis of ELP4, SRPX2, and interacting genes in typical and atypical rolandic epilepsy. *Epilepsia* **55**: e89-e93.

Rejeb I, Saillour Y, Castelnau L, Julien C, Bienvenu T, Taga P, et al. 2008. A novel splice mutation in PAK3 gene underlying mental retardation with neuropsychiatric features. *Eur J Hum Genet* **16**: 1358-1363.

Ridel KR, Leslie ND, Gilbert DL. 2005. An updated review of the long-term neurological effects of galactosemia. *Pediatr Neurol* **33**: 153-161.

Rio M, Clech L, Amiel J, Faivre L, Lyonnet S, Le Merrer M, Odent S, Lacombe D, Edery P, Brauner R, et al. 2003. Spectrum of NSD1 mutations in Sotos and Weaver syndromes. *J Med Genet* **40**: 436-440.

Rivière JB, van Bon BW, Hoischen A, Kholmanskikh SS, O’Roak BJ, Gilissen C, et al. 2012. De novo mutations in the actin genes ACTB and ACTG1 cause Baraitser-Winter syndrome. *Nat Genet* **44**: 440-444.

Rosenblatt DS, Thomas IT, Watkins D, Cooper BA, Erbe RW. 1987. Vitamin B12 responsive homocystinuria and megaloblastic anemia: heterogeneity in methylcobalamin deficiency. *Am J Med Genet* **26**: 377-383.

Rudnik-Schöneborn S, Barth PG, Zerres K. 2014. Pontocerebellar hypoplasia. *Am J Med Genet C Semin Med Genet* **166C**: 173-183.

Russo S, Cogliati F, Cavalleri F, Cassitto MG, Giglioli R, Toniolo D, et al. 2000. Mapping to distal Xq28 of nonspecific X-linked mental retardation MRX72: linkage analysis and clinical findings in a three-generation Sardinian family. *Am J Med Genet* **94**: 376-382.

Rymen D, Keldermans L, Race V, Régal L, Deconinck N, Dionisi-Vici C, Fung CW, Sturiale L, Rosnoblet C, Foulguier F, et al. 2012. COG5-CDG: expanding the clinical spectrum. *Orphanet J Rare Dis* **7**: 94.

Rymen D, Peanne R, Millón MB, Race V, Sturiale L, Garozzo D, Mills P, Clayton P, Asteggiano CG, Quelhas D, et al. 2013. MAN1B1 deficiency: an unexpected CDG-II. *PLoS Genet* **9**: e1003989.

Saitsu H, Nishimura T, Muramatsu K, Kodera H, Kumada S, Sugai K, et al. 2013. De novo mutations in the autophagy gene WDR45 cause static encephalopathy of childhood with neurodegeneration in adulthood. *Nat Genet* **45**: 445-449.

Sanjad SA, Sakati NA, Abu-Osba YK, Kaddoura R, Milner RD. 1991. A new syndrome of congenital hypoparathyroidism, severe growth failure, and dysmorphic features. *Arch Dis Child* **66**: 193-196.

Sarig O, Goldsher D, Nousbeck J, Fuchs-Telem D, Cohen-Katsenelson K, Iancu TC, Manov I, Saada A, Sprecher E, Mandel H. 2013. Infantile mitochondrial hepatopathy is a cardinal feature of MEGDEL syndrome (3-methylglutaconic aciduria type IV with sensorineural deafness, encephalopathy and Leigh-like syndrome) caused by novel mutations in SERAC1. *Am J Med Genet A* **161**: 2204-2215.

Sarimski K. 1997. Communication, social-emotional development and parenting stress in Cornelia-de-Lange syndrome. *J Intellect Disabil Res* **41**: 70-75.

Schaaf CP, Boone PM, Sampath S, Williams C, Bader PI, Mueller JM, et al. 2012. Phenotypic spectrum and genotype-phenotype correlations of NRXN1 exon deletions. *Eur J Hum Genet* **20**: 1240-1247.

Schaaf CP, Gonzalez-Garay ML, Xia F, Potocki L, Gripp KW, Zhang B, Peters BA, McElwain MA, Drmanac R, Beaudet AL, et al. 2013. Truncating mutations MAGEL2 cause Prader-Willi phenotypes and autism. *Nat Genet* **45**: 1405-1408.

Schaaf CP, Koster J, Katsonis P, Kratz L, Shchelochkov OA, Scaglia F, et al. 2011. Desmosterolosis-phenotypic and molecular characterization of a third case and review of the literature. *Am J Med Genet A* **155A**: 1597-1604.

Schaffer AE, Eggens VR, Caglayan AO, Reuter MS, Scott E, Coufal NG, et al. 2014. CLP1 founder mutation links tRNA splicing and maturation to cerebellar development and neurodegeneration. *Cell* **157**: 651-663.

Scheffer IE, Turner SJ, Dibbens LM, Bayly MA, Friend K, Hodgson B, Burrows L, Shaw M, Wei C, Ullmann R, et al. 2008. Epilepsy and mental retardation limited to females: an under-recognized disorder. *Brain* **131**: 918-927.

Schinzel A, Kaufmann U. 1986. The acrocallosal syndrome in sisters. *Clin Genet* **30**: 399-405.

Schnur RE, Greenbaum BH, Heymann WR, Christensen K, Buck AS, Reid CS. 1997. Acute lymphoblastic leukemia in a child with the CHIME neuroectodermal dysplasia syndrome. *Am J Med Genet* **72**: 24-29.

Schwartz CE, Tarpey PS, Lubs HA, Verloes A, May MM, Risheg H, et al. 2007. The original Lujan syndrome family has a novel missense mutation (p.N1007S) in the MED12 gene. *J Med Genet* **44**: 472-477.

Selicorni A, Russo S, Gervasini C, Castronovo P, Milani D, Cavalleri F, Bentivegna A, Masciadri M, Domi A, Divizia MT, et al. 2007. Clinical score of 62 Italian patients with Cornelia de Lange syndrome and correlations with the presence and type of NIPBL mutation. *Clin Genet* **72**: 98-108.

Shashi V, Zunich J, Kelly TE, Fryburg JS. 1995. Neuroectodermal (CHIME) syndrome: an additional case with long term follow up of all reported cases. *J Med Genet* **32**: 465-469.

Shen J, Gilmore EC, Marshall CA, Haddadin M, Reynolds JJ, Eyaid W, et al. 2010. Mutations in PNKP cause microcephaly, seizures and defects in DNA repair. *Nat Genet* **42**: 245-249.

Shimojima K, Sugawara M, Shichiji M, Mukaida S, Takayama R, Imai K, et al. 2011. Loss-of-function mutation of collybistin is responsible for X-linked mental retardation associated with epilepsy. *J Hum Genet* **56**: 561-565.

Shimojima K, Tanaka R, Shimada S, Sangu N, Nakayama J, Iwasaki N, Yamamoto T. 2013. A novel homozygous mutation of GJC2 derived from maternal uniparental disomy in a female patient with Pelizaeus-Merzbacher disease. *J Neurol Sci* **330**: 123-126.

Shoubridge C, Tarpey PS, Abidi F, Ramsden SL, Rujirabanjerd S, Murphy JA, et al. 2010. Mutations in the guanine nucleotide exchange factor gene IQSEC2 cause nonsyndromic intellectual disability. *Nat Genet* **42**: 486-488.

Sikora DM, Pettit-Kekel K, Penfield J, Merkens LS, Steiner RD. 2006. The near universal presence of autism spectrum disorders in children with Smith-Lemli-Opitz syndrome. *Am J Med Genet A* **140**: 1511-1518.

Simonati A, Boaretto F, Vettori A, Dabrilli P, Criscuolo L, Rizzuto N, Mostacciuolo ML. 2006. A novel missence mutation in the L1CAM gene in a boy with L1 disease. *Neurol Sci* **27**: 115-117.

Skidmore DL, Chitayat D, Morgan T, Hinek A, Fischer B, Dimopoulou A, et al. 2011. Further expansion of the phenotypic spectrum associated with mutations in ALDH18A1, encoding Δ^1^-pyrroline-5-carboxylate synthase (P5CS). *Am J Med Genet A* **155A**: 1848-1856.

Slaney SF, Goodman FR, Eilers-Walsman BL, Hall BD, Williams DK, Young ID, et al. 1999. Acromelic frontonasal dysostosis. *Am J Med Genet* **83**: 109-116.

Slavotinek AM, Searby C, Al-Gazali L, Hennekam RC, Schrander-Stumpel C, Orcana-Losa M, Pardo-Reoyo S, Cantani A, Kumar D, Capellini Q, et al. 2002. Mutation analysis of the MKKS gene in McKusick-Kaufman syndrome and selected Bardet-Biedl syndrome patients. *Hum Genet* **110**: 561-567.

Smith JD, Hing AV, Clarke CM, Johnson NM, Perez FA, Park SS, et al. 2014. Exome sequencing identifies a recurrent de novo ZSWIM6 mutation associated with acromelic frontonasal dysostosis. *Am J Hum Genet* **95**: 235-240.

Smith SE, Mullen TE, Graham D, Sims KB, Rehm HL. 2012. Norrie disease: extraocular clinical manifestations in 56 patients. *Am J Med Genet* **158A**: 1909-1917.

Smith IM, Nichols SL, Issekutz K, Blake K; Canadian Paediatric Surveillance Program. 2005. Behavioral profiles and symptoms of autism in CHARGE syndrome: preliminary Canadian epidemiological data. *Am J Med Genet* **133A**: 248-256.

Soden SE, Saunders C, Willig LK, Farrow EG, Smith LD, Petrikin JE, et al. 2014. Effectiveness of exome and genome sequencing guided by acuity of illness for diagnosis of neurodevelopmental disorders. *Sci Transl Med* **6**: 265ra168.

Sousa SB, Abdul-Rahman OA, Bottani A, Cormier-Daire V, Fryer A, Gillessen-Kaesbach G, Horn D, Josifova D, Kuechler A, Lees M, et al. 2009. Nicolaides-Baraitser syndrome: delineation of the phenotype. *Am J Med Genet A* **149A**: 1628-1640.

Steiner CE, Guerreiro MM, Marques-de-Faria AP. 2004. Brief report: acrocallosal syndrome and autism. *J Autism Dev Disord* **34**: 723-726.

Steinfeld R, Grapp M, Kraetzner R, Dreha-Kulaczewski S, Helms G, Dechent P, et al. 2009. Folate receptor alpha defect causes cerebral folate transport deficiency: a treatable neurodegenerative disorder associated with disturbed myelin metabolism. *Am J Hum Genet* **85**: 354-363.

Stevens CA, Pouncey J, Knowles D. 2011. Adults with Rubinstein-Taybi syndrome. *Am J Med Genet A* **155A**: 1680-1684.

Stocco dos Santos RC, Castro NH, Lillia Holmes A, Beçak W, Tackels-Horne D, Lindsey CJ, Lubs HA, Stevenson RE, Schwartz CE. 2003. Stocco dos Santos X-linked mental retardation syndrome: clinical elucidation and localization to Xp11.3-Xq21.3. *Am J Med Genet A* **118A**: 255-259.

Su HC. 2010. Dedicator of cytokinesis 8 (DOCK8) deficiency. *Curr Opin Allergy Clin Immunol* **10**: 515-520.

Takenouchi T, Okuno H, Kosaki R, Ariyasu D, Torii C, Momoshima S, Harada N., Yoshihashi H, Takahashi T, Awazu M, et al. 2012. Microduplication of Xq24 and Hartsfield syndrome with holoprosencephaly, ectrodactyly, and clefting. *Am J Med Genet A* **158A**: 2537-2541.

Talkowski ME, Mullegama SV, Rosenfeld JA, van Bon BW, Shen Y, Repnikova EA, Gastier-Foster J, Thrush DL, Kathiresan S, Ruderfer DM, et al. 2011. Assessment of 2q23.1 microdeletion syndrome implicates MBD5 as a single causal locus of intellectual disability, epilepsy, and autism spectrum disorder. *Am J Hum Genet* **89**: 551-563.

Tan MH, Mester J, Peterson C, Yang Y, Chen JL, Rybicki LA, et al. 2011. A clinical scoring system for selection of patients for PTEN mutation testing is proposed on the basis of a prospective study of 3042 probands. *Am J Hum Genet* **88**: 42-56.

Tan CA, Topper S, Ward Melver C, Stein J, Reeder A, Arndt K, Das S. 2014. The first case of CDK5RAP2-related primary microcephaly in a non-consanguineous patient identified by next generation sequencing. *Brain Dev* **36**: 351-355.

Tan EH, Yusoff AA, Abdullah JM, Razak SA. 2012. Generalized epilepsy with febrile seizure plus (GEFS+) spectrum: novel de novo mutation of SCN1A detected in a Malaysian patient. *J Pediatr Neurosci* **7**: 123-125.

Tanaka H, Ito J, Cho K, Mikawa M. 1993. Hirschsprung disease, unusual face, mental retardation, epilepsy, and congenital heart disease: Goldberg-Shprintzen syndrome. *Pediatr Neurol* **9**: 479-481.

Tarpey PS, Raymond FL, Nguyen LS, Rodriguez J, Hackett A, Vandeleur L, Smith R, Shoubridge C, Edkins S, Stevens S, et al. 2007. Mutations in UPF3B, a member of the nonsense-mediated mRNA decay complex, cause syndromic and nonsyndromic mental retardation. *Nat Genet* **39**: 1127-1133.

Tarpey PS, Raymond FL, O’Meara S, Edkins S, Teague J, Butler A, et al. 2007. Mutations in CUL4B, which encodes a ubiquitin E3 ligase subunit, cause an X-linked mental retardation syndrome associated with aggressive outbursts, seizures, relative macrocephaly, central obesity, hypogonadism, pes cavus, and tremor. *Am J Hum Genet* **80**: 345-352.

Tasdemir S, Sahin I, Cayır A, Doneray H, Solomon BD, Muenke M, Yuce I, Tatar A. 2014. Holoprosencephaly: ZIC2 mutation in a case with panhypopituitarism. *J Pediatr Endocrinol Metab* ***27***: 777-781.

Thomas PK, Hoffbrand AV, Smith IS. 1982. Neurological involvement in hereditary transcobalamin II deficiency. *J Neurol Neurosurg Psychiatry* **45**: 74-77.

Timal S, Hoischen A, Lehle L, Adamowicz M, Huijben K, Sykut-Cegielska J, et al. 2012. Gene identification in the congenital disorders of glycosylation type I by whole-exome sequencing. *Hum Mol Genet* **21**: 4151-4161.

Tinschert S, Anton-Lamprecht I, Albrecht-Nebe H, Audring H. 1996. Zunich neuroectodermal syndrome: migratory ichthyosiform dermatosis, colobomas, and other abnormalities. *Pediatr Dermatol* **13**: 363-371.

Tischfield MA, Bosley TM, Salih MA, Alorainy IA, Sener EC, Nester MJ, et al. 2005. Homozygous HOXA1 mutations disrupt human brainstem, inner ear, cardiovascular and cognitive development. *Nat Genet* **37**: 1035-1037.

Tohyama J, Akasaka N, Osaka H, Maegaki Y, Kato M, Saito N, et al. 2008. Early onset West syndrome with cerebral hypomyelination and reduced cerebral white matter. *Brain Dev* **30**: 349-355.

Tohyama J, Akasaka N, Saito N, Yoshimura J, Nishiyama K, Kato M. 2007. Megalencephaly and polymicrogyria with polydactyly syndrome. *Pediatr Neurol* **37**: 148-151.

Tory K, Lacoste T, Burglen L, Morinière V, Boddaert N, Macher MA, Llanas B, Nivet H, Bensman A, Niaudet P, et al. 2007. High NPHP1 and NPHP6 mutation rate in patients with Joubert syndrome and nephronophthisis: potential epistatic effect of NPHP6 and AHI1 mutations in patients with NPHP1 mutations. *J Am Soc Nephrol* *18*, 1566-1575.

Touraine RL, Attié-Bitach T, Manceau E, Korsch E, Sarda P, Pingault V, Encha-Razavi F, Pelet A, Augé J, Nivelon-Chevallier A, et al. 2000. Neurological phenotype in Waardenburg syndrome type 4 correlates with novel SOX10 truncating mutations and expression in developing brain. *Am J Hum Genet* **66**: 1496-1503.

Tovetjärn R, Tarnow P, Maltese G, Fischer S, Sahlin PE, Kölby L. 2012. Children with Apert syndrome as adults: a follow-up study of 28 Scandinavian patients. *Plast Reconstr Surg* **130**: 572e-576e.

Tran Mau-Them F, Willems M, Albrecht B, Sanchez E, Puechberty J, Endele S, et al. 2014. Expanding the phenotype of IQSEC2 mutations: truncating mutations in severe intellectual disability. *Eur J Hum Genet* **22**: 289-292.

Trillingsgaard A, ØStergaard JR. 2004. Autism in Angelman syndrome: an exploration of comorbidity. *Autism* **8**: 163-174.

Turner G, Partington M, Kerr B, Mangelsdorf M, Gecz J. 2002. Variable expression of mental retardation, autism, seizures, and dystonic hand movements in two families with an identical ARX gene mutation. *Am J Med Genet* **112**: 405-411.

Tylki-Szymanska A, Gradowska W, Sommer A, Heer A, Walter M, Reinhard C, Omran H, Sass JO, Jurecka A. 2010. Aminoacylase 1 deficiency associated with autistic behavior. *J Inherit Metab Dis* **33**: S211-S214.

Valstar MJ, Bruggenwirth HT, Olmer R, Wevers RA, Verheijen FW, Poorthuis BJ, Halley DJ, Wijburg FA. 2010. Mucopolysaccharidosis type IIIB may predominantly present with an attenuated clinical phenotype. *J Inherit Metab Dis* **33**: 759-767.

van Balkom ID, Shaw A, Vuijk PJ, Franssens M, Hoek HW, Hennekam RC. 2011. Development and behavior in Marshall-Smith syndrome: an exploratory study of cognition, phenotype and autism. *J Intellect Disabil Res* **55**: 973-987.

van Balkom ID, Vujik PJ, Franssens M, Hoek HW, Hennekam RC. 2012. Development, cognition, and behavior in Pitt-Hopkins syndrome. *Dev Med Child Neurol* **54**: 925-931.

van der Crabben SN, Harakalova M, Brilstra EH, van Berkestijin FM, Hofstede FC, van Vught AJ, et al. 2014. Expanding the spectrum of phenotypes associated with germline PIGA mutations: a child with developmental delay, accelerated linear growth, facial dysmorphisms, elevated alkaline phosphatase, and progressive CNS abnormalities. *Am J Med Genet* **164A**: 29-35.

van der Knaap MS, Jakobs C, Hoffmann GF, Duran M, Muntau AC, Schweiter S, 1999. D-2-hydroxyglutaric aciduria: further clinical delineation. *J Inherit Metab Dis* **22**: 404-413.

van der Knaap MS, Valk J. 1989. The reflection of histology in MR imaging of Pelizaeus-Merzbacher disease. *AJNR Am J Neuroradiol* **10**: 99-103.

Vanhanen SL, Puranen J, Autti T, Raininko R, Liewendahl K, Nikkinen P, et al. 2004. Neuroradiological findings (MRS, MRI, SPECT) in infantile neuronal ceroid-lipofuscinosis (infantile CLN1) at different stages of the disease. *Neuropediatrics* **35**: 27-35.

van Kuilenburg AB, Dobritzsch D, Meijer J, Meinsma R, Benoist JF, Assmann B, Schubert S, Hoffmann GF, Duran M, de Vries MC, et al. 2010. Dihydropyrimidinase deficiency: phenotype, genotype and structural consequences in 17 patients. *Biochim. Biophys. Acta* **1802**: 639-648.

van Maldergem L, Hou Q, Kalscheuer V, Rio M, Doco-Fenzy M, Medeira A, de Brouwer AP, Cabrol C, Haas SA, Cacciagli P, et al. 2013. Loss of function of KIAA2022 causes mild to severe intellectual disability with an autism spectrum disorder and impairs neurite outgrowth. *Hum Mol Genet* **22**: 3306-3314.

Varho TT, Alojoki LE, Posti KM, Korhonen TT, Renlund MG, Nyman SR, Sillanpää ML, Aula PP. 2002. Phenotypic spectrum of Salla disease, a free sialic acid storage disorder. *Pediatr Neurol* **26**: 267-273.

Verpoorten N, Claeys KG, Deprez L, Jacobs A, Van Gerwen V, Lagae L, Arts WF, De Meirleir L, Keymolen K, Ceuterick-de Groote C, et al. 2006. Novel frameshift and splice site mutations in the neurotrophic tyrosine kinase receptor type 1 gene (NTRK1) associated with hereditary sensory neuropathy type IV. *Neuromuscul Disord* **16**: 19-25.

Vija L, Archambaud F, Chaumet-Riffaud P. 2012. Bone scan and IGF1 deficiency: case report. *Médecine Nucléaire* **36**: 399-402.

Vulto-van Silfhout AT, Rajamanickam S, Jensik PJ, Vergult S, de Rocker N, Newhall KJ, et al. 2014. Mutations affecting the SAND domain of DEAF1 cause intellectual disability with severe speech impairment and behavioral problems. *Am J Hum Genet* **94**: 649-661.

Wada T, Gibbons RJ. 2003. ATR-X syndrome. In: *Genetics and Genomics of Neurobehavioral Disorders*. (ed. Fish GS). Humana Press: Totowa, NJ, pp. 309-334.

Walker AP, Muscatelli F, Stafford AN, Chelly J, Dahl N, Blomquist HK, Delanghe, J, Willems PJ, Steinmann B, Monaco AP. 1996. Mutations and phenotype in isolated glycerol kinase deficiency. *Am J Hum Genet* **58**: 1205-1211.

Wallace SE, Conta JH, Winder TL, Willer T, Eskuri JM, Haas R, Patterson K, Campbell KP, Moore SA, Gospe SM, Jr. 2014. A novel missense mutation in POMT1 modulates the severe congenital muscular dystrophy phenotype associated with POMT1 nonsense mutations. *Neuromuscul Disord* **24**: 312-320.

Wang H, Bright A, Xin B, Bockoven JR, Paller AS. 2013. Cutaneous dyspigmentation in patients with gangiolside GM3 synthase deficiency. *Am J Med Genet A* **161A**: 875-879.

Wang L, Jin X, Zhao X, Liu D, Hu T, Li W, Jiang L, Dan H, Zeng X, Chen Q. 2014. Focal dermal hypoplasia: updates. *Oral Dis* **20**: 17-24.

Wang D, Kranz-Eble P, De Vivo DC. 2000. Mutational analysis of GLUT1 (SLC2A1) in Glut-1 deficiency syndrome. *Hum Mutat* **16**: 224-231.

Wang P-J, Young C, Liu H-M, Chang Y-C, Shen Y-Z. 1995. Neurophysiologic studies and MRI in Pelizaeus-Merzbacher disease: comparison of classic and connatal forms. *Pediatr Neurol* **12**: 47-53.

Weckhuysen S, Mandelstam S, Suls A, Audenaert D, Deconinck T, Claes LR, et al. 2012. KCNQ2 encephalopathy: emerging phenotype of a neonatal epileptic encephalopathy. *Ann Neurol* **71**: 15-25.

Willemsen MA, IJlst L, Steijlen PM< Rotteveel JJ, de Jong JG, van Domburg PH, et al. 2001. Clinical, biochemical and molecular genetic characteristics of 19 patients with Sjögren-Larsson syndrome. *Brain* **124**: 1426-1437.

Willemsen MH, Vulto-van Silfhout AT, Nillesen WM, Wissink-Lindhout WM, van Bokhoven H, Philip N, Berry-Kravis EM, Kini U, van Ravenswaaij-Arts CM, Delle Chiaie B, et al. 2012. Update on Kleefstra syndrome. *Mol Syndromol* **2**: 202-212.

Williams MS. 2006. Neuropsychological evaluation in Lujan-Fryns syndrome: commentary and clinical report. *Am J Med Genet A* **15**: 2812-2825.

Wilson A, Leclerc D, Saberi F, Campeau E, Hwang HY, Shane B, et al. 1998. Functionally null mutations in patients with the cbIG-variant form of methionine synthase deficiency. *Am J Hum Genet* **63**: 409-414.

Wolthuis DF, van Asbeck E, Mohamed M, Gardeitchik T, Lim-Melia ER, Wevers RA, et al. 2014. Cutis laxa, fat pads and retinopathy due to ALDH18A1 and review of the literature. *Eur J Paediatr Neurol* **18**: 511-515.

Writzl K, Primec ZR, Stražišar BG, Osredkar D, Pečarič-Meglič N, Kranjc BS, et al. 2012. Early onset West syndrome with severe hypomyelination and coloboma-like optic discs in a girl with SPTAN1 mutation. *Epilepsia* **53**: e106-110.

Yang AC, Ng BG, Moore SA, Rush J, Waechter CJ, Raymond KM, et al. 2013. Congenital disorder of glycosylation due to DPM1 mutations presenting with dystroglycanopathy-type congenital muscular dystrophy. *Mol Genet Metab* **110**: 345-351.

Yesil G, Guler S, Yuksel A, Alanay Y. 2014. Report of a patient with Temple-Baraitser syndrome. *Am J Med Genet A* **164A**: 848-851.

Yıldırım Y, Orhan EK, Iseri SA, Serdaroglu-Oflazer P, Kara B, Solakoğlu S, et al. 2011. A frameshift mutation of ERLIN2 in recessive intellectual disability, motor dysfunction and multiple joint contractures. *Hum Mol Genet* **20**: 1886-1892.

Yntema HG, Hamel BC, Smits AP, van Roosmalen T, van den Helm B, Kremer H, Ropers HH, Smeets DF, van Bokhoven H. 1998. Localisation of a gene for non-specific X linked mental retardation (MRX46) to Xq25-q26. *J Med Genet* **35**: 801-805.

Yoneda Y, Saitsu H, Touyama M, Makita Y, Miyamoto A, Hamada K, Kurotaki N, Tomita H, Nishiyama K, Tsurusaki Y, et al. 2012. Missense mutations in the DNA-binding/dimerization domain of NFIX cause Sotos-like features. *J Hum Genet* **57**: 207-211.

Zavadáková P, Fowler B, Suormala T, Novotna Z, Mueller P, Hennermann JB, et al. 2005. cbIE type of homocystinuria due to methionine synthase reductase deficiency: functional correction by minigene expression. *Hum Mutat* **25**: 239-247.

Zafeiriou DI, Ververi A, Salomons GS, Vargiami E, Haas D, Papadopoulou V, Kontopoulos E, Jaokbs C. 2008. L-2-hydroxyglutaric aciduria presenting with severe autistic features. *Brain Dev* **30**: 305-307.

Zaffanello M, Zamboni G, Fontana E, Zoccante L, Tatò L. 2003. A case of partial biotinidase deficiency associated with autism. *Child Neuropsychol* **9**: 184-188.

Zecavati N, Spence SJ. 2009. Neurometabolic disorders and dysfunction in autism spectrum disorders. *Curr Neurol Neurosci Rep* **9**: 129-136.

Zhang X, Ling J, Barcia G, Jing L, Wu J, Barry BJ, et al. 2014. Mutations in QARS, encoding glutaminyl-tRNA synthetase, cause progressive microcephaly, cerebral-cerebellar atrophy, and intractable seizures. *Am J Hum Genet* **94**: 547-558.

Zolotushko J, Flusser H, Markus B, Shelef I, Langer Y, Heverin M, et al. 2011. The desmosterolosis phenotype: spasticity, microcephaly and micrognathia with agenesis of corpus callosum and loss of white matter. *Eur J Hum Genet* **19**: 942-946.

Zschocke J. 2012. HSD10 disease: clinical consequences of mutations in the HSD17B10 gene. *J Inherit Metab Dis* **35**: 81-89.
